# Pragmatic Risk Stratification Method to Identify Emergency Department Presentations for Alternative Care Service Pathways: Registry-Based Retrospective Study Over 5 Years

**DOI:** 10.2196/73758

**Published:** 2025-05-12

**Authors:** John Rong Hao Tay, Yohei Okada, Gayathri Devi Nadarajan, Fahad Javaid Siddiqui, Tomás Barry, Marcus Eng Hock Ong

**Affiliations:** 1 Health Services and Systems Research Programme Duke-NUS Medical School Singapore Singapore; 2 Department of Restorative Dentistry National Dental Centre of Singapore Singapore Singapore; 3 Department of Preventive Services, Graduate School of Medicine Kyoto University Kyoto Japan; 4 Pre-hospital & Emergency Research Centre Duke-NUS Medical School Singapore Singapore; 5 Department of Emergency Medicine Singapore General Hospital Singapore Singapore; 6 School of Medicine University College Dublin Dublin Ireland

**Keywords:** emergency services utilization, health services, triage, electronic health records, clinical decision-making, cluster analysis

## Abstract

**Background:**

Redirecting avoidable presentations to alternative care service pathways (ACSPs) may lead to better resource allocation for prehospital emergency care. Stratifying emergency department (ED) presentations by admission risk using diagnosis codes might be useful in identifying patients suitable for ACSPs.

**Objective:**

We aim to cluster *ICD-10* (*International Statistical Classification of Diseases, Tenth Revision*) diagnosis codes based on hospital admission risk, identify ED presentation characteristics associated with these clusters, and develop an exploratory classification to identify groups potentially suitable for ACSPs.

**Methods:**

Retrospective observational data from a database of all visits to the ED of a tertiary care institution for over 5 years (2016-2020) were analyzed. K-means clustering grouped diagnosis codes according to admission outcomes. Multivariable logistic regression was performed to determine the association of characteristics with cluster membership. *ICD-10* codes were grouped into blocks and analyzed for cumulative coverage to identify dominant groups associated with lower hospital admission risk.

**Results:**

A total of 215,477 ambulatory attendances classified as priority levels 3 (ambulatory) and 4 (nonemergency) under the Patient Acuity Category Scale were selected, with a 17.3% (0.4%) overall admission rate. The mean presentation age was 46.2 (SD 19.4) years. Four clusters with varying hospital admission risks were identified. Cluster 1 (n=131,531, 61%) had the lowest admission rate at 4.7% (0.2%), followed by cluster 2 (n=44,347, 20.6%) at 19.5% (0.4%), cluster 3 (n=27,829, 12.9%) at 47.8% (0.5%), and cluster 4 (n=11,770, 5.5%) with the highest admission rate at 78% (0.4%). The four-cluster solution achieved a silhouette score of 0.65, a Calinski-Harabasz Index of 3649.5, and a Davies-Bouldin Index of 0.46. Compared to clustering based on *ICD-10* blocks, clustering based on individual *ICD-10* codes demonstrated better separation. Mild (odds ratio [OR] 2.55, 95% CI 2.48-2.62), moderate (OR 2.40, 95% CI 2.28-2.51), and severe (OR 3.29, 95% CI 3.13-3.45) Charlson Comorbidity Index scores increased the odds of admission. Tachycardia (OR 1.46, 95% CI 1.43-1.49), hyperthermia (OR 2.32, 95% CI 2.25-2.40), recent surgery (OR 1.31, 95% CI 1.27-1.36), and recent inpatient admission (OR 1.16, 95% CI 1.13-1.18) also increased the odds of higher cluster membership. Among 132 *ICD-10* blocks, 17 blocks accounted for 80% of cluster 1 cases, including musculoskeletal or connective tissue disorders and head or lower limbs injuries. Higher-risk categories included respiratory tract infections such as influenza and pneumonia, and infections of the skin and subcutaneous tissue.

**Conclusions:**

Most ambulatory presentations at the ED were categorized into low-risk clusters with a minimal likelihood of hospital admission. Stratifying *ICD-10* diagnosis codes by admission outcomes and ranking them based on frequency provides a structured approach to potentially stratify admission risk.

## Introduction

The emergency care system is a critical part of health care that provides accessible and time-sensitive care for acute illnesses and injuries. However, worldwide, it faces increasing challenges due to growing demand, driven in many cases by aging populations and the complexity of care [1]. Suboptimal usage of prehospital emergency care (PEC) refers to the activation of the system for conditions that may otherwise be treated by alternative care service pathways (ACSPs), without the need for urgent transport to the emergency department (ED). Worldwide, the use of ED services is rising, with avoidable visits estimated to comprise 20%-40% of all ED attendances [2-4]. Such nonurgent visits contribute to ED overcrowding, prolonged waiting times, increased risk of medication errors, and poorer health outcomes [5-8]. Redirecting ED visits to ACSPs may lead to better allocation of resources within PEC, ensuring that the ED is reserved for urgent cases. This approach reduces system-wide stress and delays, while low-acuity patients can be managed safely in appropriate care settings [9-11].

One approach to identifying ED presentations suitable for ACSPs is to stratify them based on their risk of hospital admission. However, accurately distinguishing suitable cases for ACSPs from those requiring urgent care is challenging due to variability in presentation and clinical complexity. A potential solution is to use diagnosis codes, which provide a clear and comprehensive reflection of a patient’s care needs compared to triage information. Using diagnosis codes provides a framework to understand population-level needs that may inform emergency care service delivery. Although the *ICD-10* (*International Statistical Classification of Diseases, Tenth Revision*) coding system provides a detailed classification with over 68,000 unique codes, which are organized into chapters and subsections, its high level of granularity poses challenges for practical applications such as admission risk stratification [12]. Aggregating these codes into meaningful categories that retain predictive relevance while minimizing diagnostic oversimplification is a challenge [13].

Clustering methods have previously been applied to various ED cohorts, primarily focusing on demographic and comorbidity data [14,15]. However, the variables used in these studies to define cluster characteristics were selected based on traits deemed clinically relevant, without a clear or quantitative explanation. Similarly, further research has clustered patients based on the co-occurrence of diagnoses, using diagnosis codes to identify patterns of multimorbidity in the ED [12]. While informative at a systems level, this approach did not incorporate hospital admission risk. To the authors’ best knowledge, there are no previous studies that clustered ED patients according to admission risk based on diagnosis codes.

Therefore, this study aimed to cluster *ICD-10* diagnosis codes based on hospital admission risk and identify ED presentation characteristics associated with these clusters; and to develop an exploratory classification to identify groups potentially suitable for ACSPs. As a hypothesis-generating study, it does not aim to establish a definitive classification but provides a foundation for ongoing efforts in Singapore to identify patient groups most amenable to ACSPs. These findings contribute to a broader initiative to reduce avoidable ED presentations and expand ACSPs.

## Methods

### Study Design

This was a retrospective single-center observational study, following the RECORD (Reporting of Studies Conducted Using Observational Routinely-Collected Data) guidelines [16]. Ethics approval from the National University of Singapore institutional review board (NUS-IRB-2025-60) was obtained.

### Study Setting and Population

Singapore is a dense urban city-state with a population of approximately 6 million and a land area of 734 km². Singapore’s health care system comprises a mix of public and private providers, with the public sector managing most health care needs [17]. While the annual population growth rate of Singapore is approximately 1.1%, ED attendance has risen disproportionately, increasing around 5.6% annually between 2005 and 2016 [18,19]. This study’s hospital is a tertiary care institution and the largest of Singapore’s 10 acute care public hospitals, accounting for one-fifth of total acute hospital beds nationwide. The SingHealth (Singapore Health Services) cluster primarily serves the eastern region of Singapore, of which this hospital is a part. This study’s hospital serves a local population of more than 1 million people, with the ED managing approximately 130,000 visits, generating 40,000 inpatient admissions each year [20].

### Study Protocol

This study used an established ED database, which includes deidentified data extracted from the SingHealth electronic health intelligence system, a comprehensive data integration platform within the SingHealth cluster. This platform consolidates data from multiple hospital systems, including clinical records, ED registrations, admissions, as well as operations and finance data sources. To maintain patient confidentiality, all data were deidentified by masking details of their ED case number, admission case number, and patient identification number. Electronic health records from other public and private hospitals were not available.

Data from January 2016 to December 2020 were extracted, during which the SNOMED-CT (Systematized Nomenclature of Medicine Clinical Terms) was exclusively used for assigning diagnosis codes. SNOMED-CT provides a comprehensive classification that is useful for clinical care and documentation. However, it is a concept model, which represents clinical ideas such as diseases, symptoms, and procedures; and has a polyhierarchical structure, where a single concept can belong to multiple categories, presenting challenges for health services reporting and research [21]. In contrast, the *ICD-10* system offers a standardized and hierarchical classification framework better suited for such analyses [22,23]. Thus, SNOMED-CT codes were converted to the *ICD-10* system using the *ICD-10-CM* (*International Classification of Diseases, Tenth Revision, Clinical Modification*) Official Guidelines for Coding and Reporting by the National Library of Medicine [22]. When exact matches were unavailable or multiple mappings existed, manual review and prioritization were applied per *ICD-10-CM* coding guidelines.

This study included ED records for patients of all ages classified as priority levels 3 (ambulatory) and 4 (nonemergency) under the Patient Acuity Category Scale [24], representing ambulatory patients requiring relatively “stable” ED care [11]. Exclusion criteria were records for priority level 1 (resuscitation) and 2 (critical care) patients, who required critical or urgent care, as well as those with records outside the timeframe of interest (2015-2020).

### Outcome

The primary outcome was hospital admission, defined as a binary variable (yes/no), based on the final disposition of each presentation to the ED.

### Cluster Analysis

K-means clustering, an unsupervised machine learning approach, was used to group *ICD-10* codes according to hospital admission outcomes. Each presentation was categorized by its primary diagnosis, with only one diagnosis analyzed per presentation. Admission risk was calculated for each *ICD-10* code and grouped into clusters. This approach was chosen to identify natural groupings in hospital admission probabilities directly from the data, rather than relying on predefined categories, such as dividing admission probabilities into quartiles. A clustering approach may reveal clinically relevant thresholds for stratifying presentations according to their likelihood of hospital admission [25].

This involved random initialization of cluster centroids, followed by the assignment of each presentation to the nearest cluster based on its distance to the centroids. The cluster centroids were then recomputed as the mean position of all presentations within each cluster. This process of reassigning presentations to clusters based on the updated centroids and recalculating the centroids was repeated iteratively until convergence, where the centroids stabilized and no longer changed significantly [26]. To reduce the potential influence of outliers, a cutoff was applied to exclude *ICD-10* codes with fewer than 15 presentations per group. This threshold was chosen to maintain the reliability of the analyses while ensuring that excluded data accounted for less than 1% of the cumulative sample size [26-28].

The optimal number of clusters for K-means clustering was assessed using the gap statistic and an elbow plot, which evaluates within-cluster variance to identify the point of diminishing returns in adding clusters. The clustering performance was further evaluated using overall Silhouette scores, the Calinski-Harabasz Index, and the Davies-Bouldin Index to evaluate how well separated the clusters were [29,30]. Silhouette scores assess how well data points fit within their cluster compared to the other clusters. Values closer to 1 indicate a higher degree of separation, while values near 0 indicate that data points are near the boundary between clusters, and values approaching –1 indicate misclassification. This was visualized through Silhouette plots [31]. The Calinski-Harabasz Index measures the ratio of intercluster dispersion to within-cluster dispersion, with a higher score indicating better separation [32]. The Davies-Bouldin Index calculates the average of similarity between clusters, with a lower score showing higher cluster separation and compactness [33].

### Statistical Analyses

For descriptive analysis, Charlson Comorbidity Index (CCI) was categorized into none, mild (1-2), moderate (3-4), and severe (≥5) [34]; systolic blood pressure into hypotension (<90 mm Hg), normal (90-130 mm Hg), and hypertension (>130 mm Hg); pulse rate into bradycardia (<60 bpm), normal (60-90 bpm), and tachycardia (>90 bpm); and temperature into hypothermia (<35.5 °C), normal (35.5-37.5 °C), and hyperthermia (>37.5 °C). Categorical and binary variables were summarized as numbers and percentages, while continuous variables were reported as means with SDs. Missing data were assessed across variables to determine the need for imputation.

To determine the association of ED presentation characteristics with cluster membership, multivariable ordinal logistic regression was performed, using cluster assignment as the outcome variable. Variables in the regression model included age, sex, ethnicity, systolic blood pressure, pulse rate, temperature, CCI, past ED visits, inpatient admissions, surgeries, intensive care unit admissions, high dependency admissions, intermediate care area admissions, and infectious disease admissions within the last 6 months. CCI was determined based on 17 predefined comorbidities from the last 5 years of hospital discharge records [35,36].

**Figure 1:**
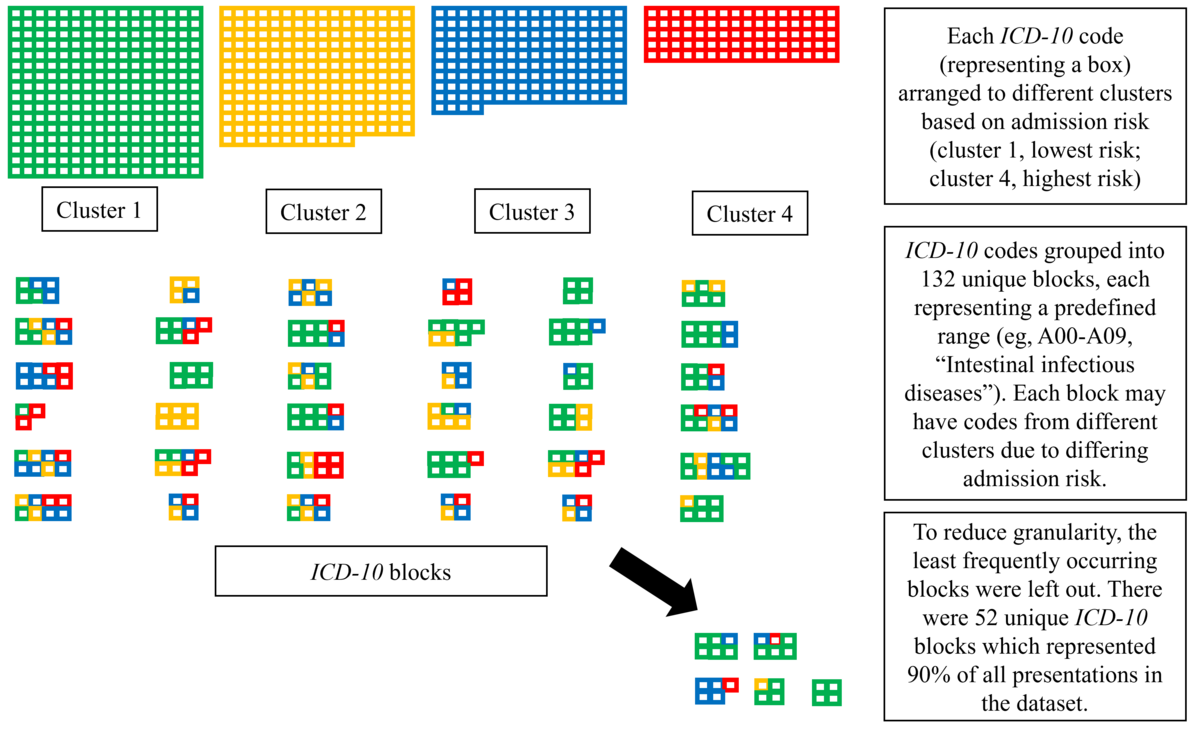
Classification of ICD-10 codes by admission risk and simplification through grouping. ICD-10: International Classification of Diseases-10.

*ICD-10* codes were categorized based on their first 3 characters (eg, A01), and grouped into standardized blocks based on the World Health Organization’s classification system to create clinically meaningful categories [37]. Each block represented a predefined range of *ICD-10* codes. For example, J00-J06 and L50-54 represented “acute respiratory infections” and “urticaria and erythema,” respectively. Consequently, individual codes within the same block could belong to different clusters. To further reduce dimensionality, an iterative process was used to identify the most frequently occurring blocks. Blocks were ranked by their frequency across the dataset to calculate the cumulative coverage achieved at different thresholds. Heatmaps were generated to visualize the proportional distribution of these blocks across clusters. Cumulative coverage thresholds (eg, 60%, 70%, 80%, 90%, or 100%) were applied, illustrating how block-level distributions evolved as lower-frequency categories were excluded (Figure 1). This approach allowed for the identification of dominant *ICD-10* blocks within clusters, offering a concise yet comprehensive representation of the data. A sensitivity analysis was conducted using a more inclusive threshold of *ICD-10* codes with at least 10 presentations per group, to assess whether the number and identity of *ICD-10* blocks changed when rarer presentations were included in the analysis. Furthermore, clustering was performed at the level of *ICD-10* blocks, and clustering performance was evaluated using overall Silhouette scores, the Calinski-Harabasz Index, and the Davies-Bouldin Index. As this study used secondary data, no additional steps to blind the covariates were applicable. Data wrangling and analyses were conducted using R (version 4.3.2, R Core Team). We calculated 95% CIs to provide estimates of the precision of the observed estimates.

### Ethical Considerations

This study was approved by the National University of Singapore institutional review board (NUS-IRB-2025-60). Waiver of consent was granted during the development of the ED database from the hospital’s electronic health records (CIRB Ref: 2021/2122). All data were deidentified, and extracted from the SingHealth electronic health intelligence system, a comprehensive data integration platform within the SingHealth cluster.

## Results

### ED Presentation Characteristics

**Figure 2:**
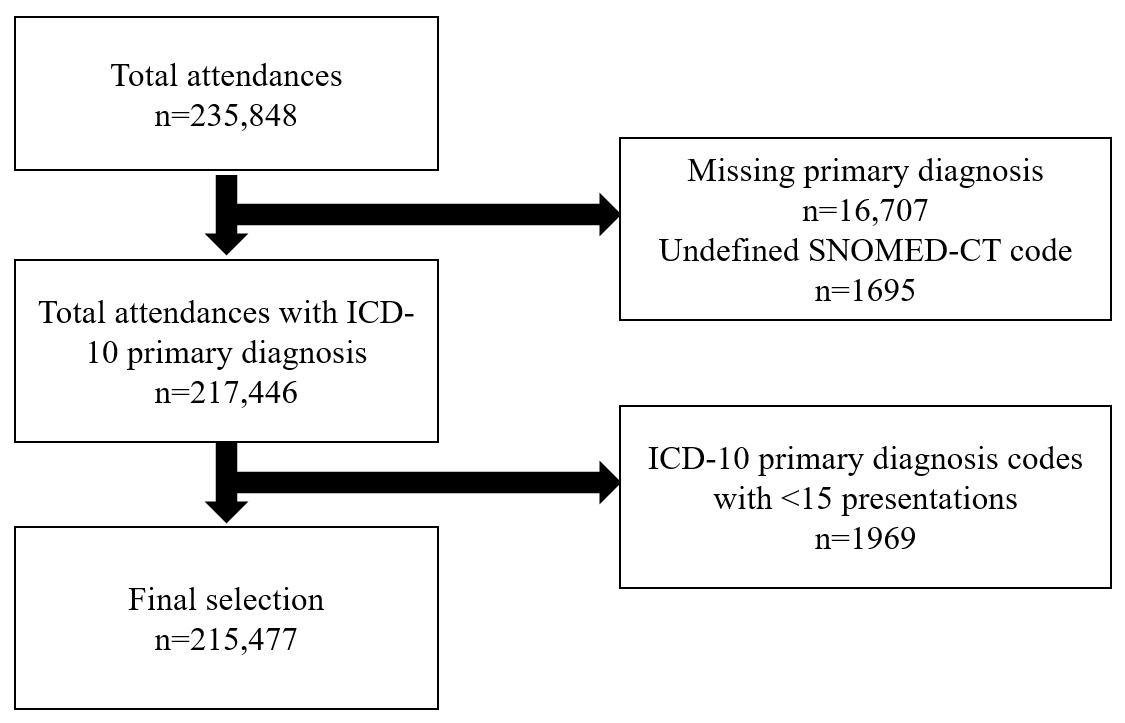
Flowchart of ED attendance selection at a tertiary care hospital in Singapore (2016-2020). ED: emergency department; ICD-10: International Statistical Classification of Diseases, Tenth Revision; SNOMED-CT: Systematized Nomenclature of Medicine Clinical Terms.

A total of 215,477 ED presentations were included in this study (Multimedia Appendix 1). Further, 16,707 (7.1%) presentations were excluded as they were missing a primary diagnosis code. Additionally, 1695 (0.7%) further presentations were excluded as they were diagnosed with an ill-defined SNOMED-CT diagnosis that did not permit mapping to an *ICD-10* code (Table S1 in Multimedia Appendix 2). An additional 1969 presentations were excluded due to low frequency (n<15) of the same *ICD-10* primary diagnosis (Figure 2 and Figure S5 in Multimedia Appendix 2). Of the 215,477 ED presentations, 29,255 (13.6%) cases had a secondary diagnosis. Secondary diagnoses were not analyzed further due to their relatively low number and to maintain analytical consistency across presentations. Missing data for vital signs constituted around 1% of the data and was previously imputed with the mean or median value during database development [35,38]. Among the included presentations, there was no missing data across the analyzed variables, except for ethnicity, which had one missing value. Given the minimal extent of missingness, multiple imputation was deemed unnecessary, and the ethnicity value was imputed using the modal category (Chinese).

The mean age of ED presentation was 46.2 (SD 19.4) years. Most presentations (176,500/215,477, 81.9%) had no comorbidities, while 11% (23,631/215,477) had a mild CCI score of 1-2 (Table 1). The overall hospital admission rate for the included presentations was 17.3% (SD 0.4%; 27,300/215,477), ranging from 4.7% (SD 0.2%; 6172/131,531) for the lower admission risk cluster (cluster 1) to 78% (SD, 0.4%; 9184/11,770) for the highest admission risk cluster (cluster 4).

**Table 1:** ED^a^ presentation characteristics at a tertiary care hospital in Singapore (2016-2020).

Characteristics			Overall	Cluster 1	Cluster 2	Cluster 3	Cluster 4
Size, n (%)			215,477 (100)	131,531 (61)	44,347 (20.6)	27,829 (12.9)	11,770 (5.5)
Admission rate, % (SD)			17.3 (0.4)	4.7 (0.2)	19.5 (0.4)	47.8 (0.5)	78 (0.4)
**Sex, n (%)**							
	Female		100,457 (46.6)	59,870 (45.5)	22,070 (49.8)	13,093 (47.1)	5424 (46.1)
	Male		115,020 (53.4)	71,661 (54.5)	22,277 (50.2)	14,736 (53)	6346 (53.9)
	Age, mean (SD)		46.2 (19.4)	44.8 (19.2)	45.4 (19.4)	49.2 (19.2)	57.1 (18.2)
**Ethnicity** **, n (%)**							
	Chinese		125,780 (58.4)	74,771 (56.9)	26,273 (59.2)	16,805 (60.4)	7931 (67.4)
	Indian		33,468 (15.5)	21,455 (16.3)	6433 (14.5)	4255 (15.3)	1325 (11.3)
	Malay		26,330 (12.2)	16,002 (12.2)	5590 (12.6)	3369 (12.1)	1369 (11.6)
	Others		29,899 (13.9)	19,303 (14.7)	6051 (13.6)	3400 (12.2)	1145 (9.7)
**Charlson Comorbidity Index, n (%)**							
	None		176,500 (81.9)	115,679 (88)	36,214 (81.7)	19,070 (68.5)	5537 (47)
	Mild		23,631 (11)	10,716 (8.2)	5004 (11.3)	5031 (18.1)	2880 (24.5)
	Moderate		8112 (3.8)	3088 (2.4)	1685 (3.8)	1815 (6.5)	1524 (13)
	Severe		7234 (3.4)	2048 (1.6)	1444 (3.3)	1913 (6.9)	1829 (15.5)
**Systolic blood pressure, n (%)**							
	Normal		125,893 (58.4)	79,655 (60.6)	25,565 (57.7)	14,971 (53.8)	5702 (48.5)
	Hypotension		298 (0.1)	163 (0.1)	58 (0.1)	57 (0.2)	20 (0.2)
	Hypertension		89,286 (41.4)	51,713 (39.3)	18,724 (42.2)	12,801 (46)	6048 (51.4)
**Pulse rate, n (%)**							
	Normal		150,361 (69.8)	96,421 (73.3)	30,565 (68.9)	16,470 (59.2)	6905 (58.7)
	Bradycardia		7649 (3.6)	4859 (3.7)	1515 (3.4)	866 (3.1)	409 (3.5)
	Tachycardia		57,467 (26.7)	30,251 (23)	12,267 (27.7)	10,493 (37.7)	4456 (37.9)
**Temperature**							
	Normal, n (%)		193,566 (89.8)	121,729 (92.6)	40,389 (91.1)	22,152 (79.6)	9296 (79)
	Hypothermia, n (%)		4382 (2)	2602 (2)	1032 (2.3)	479 (1.7)	269 (2.3)
	Hyperthermia, n (%)		17,529 (8.1)	7200 (5.5)	2926 (6.6)	5198 (18.7)	2205 (18.7)
	Number of previous ED visits, mean (SD)		0.6 (1.9)	0.7 (2)	0.6 (1.9)	0.5 (1.3)	0.6 (1.3)
	Number of previous inpatient visits, mean (SD)		0.2 (0.6)	0.1 (0.6)	0.2 (0.7)	0.2 (0.6)	0.3 (0.8)
	Number of recent surgeries, mean (SD)		0 (0.3)	0 (0.2)	0 (0.3)	0.1 (0.4)	0.1 (0.6)
	Number of previous ICU^b^ admissions, mean (SD)		0 (0.1)	0 (0.1)	0 (0.1)	0 (0.1)	0 (0.1)
	Previous HD^c^ admissions, mean (SD)		0 (0.2)	0 (0.1)	0 (0.2)	0 (0.2)	0 (0.2)
	Previous ICA^d^ admissions, mean (SD)	0 (0.1)		0 (0.1)	0 (0.1)	0 (0.1)	0 (0.2)
	Previous infectious disease admissions, mean (SD)	0 (0.4)		0 (0.3)	0 (0.4)	0 (0.5)	0.1 (0.7)

^a^ED: emergency department.

^b^ICU: intensive care unit.

^c^HD: high dependency.

^d^ICA: intermediate care area.

### Cluster Analysis

**Figure 3:**
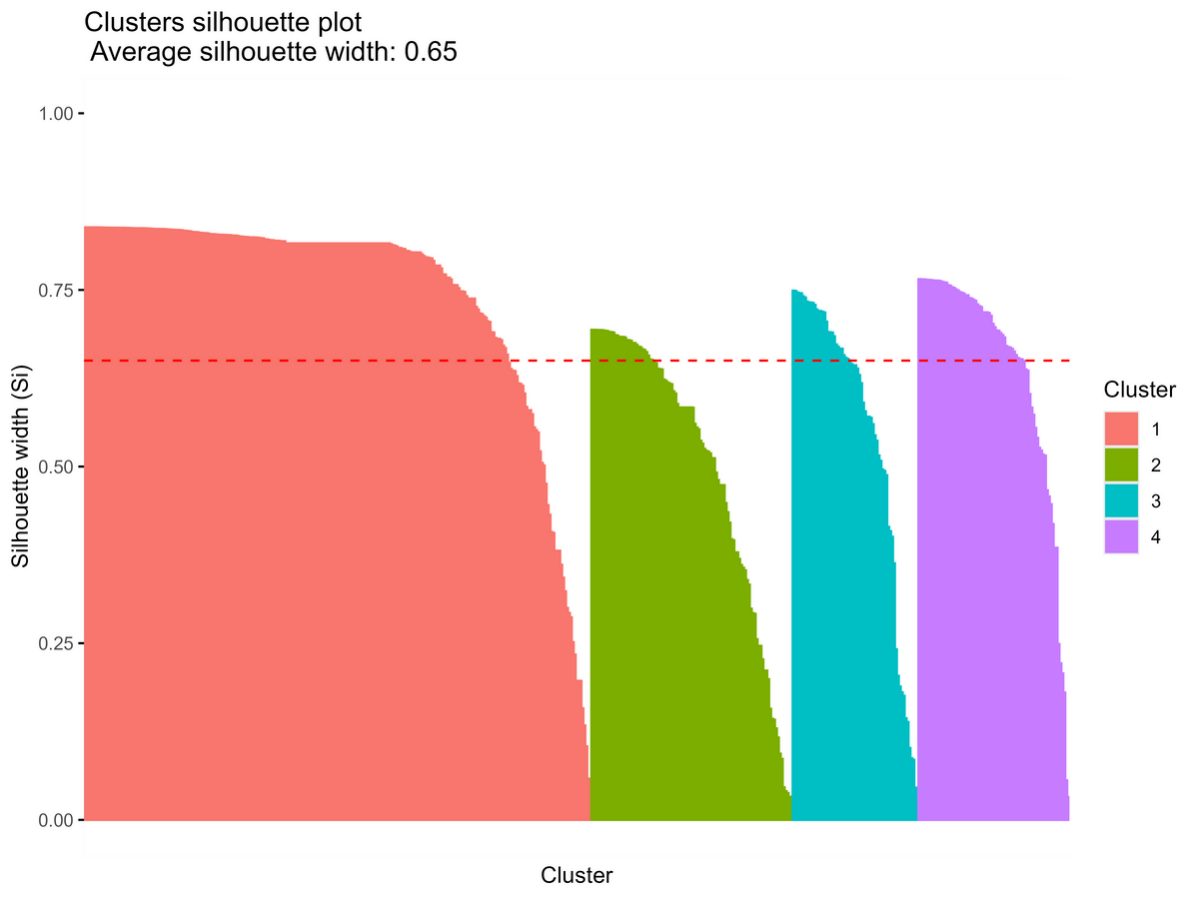
Silhouette plot using K-means clustering. All clusters have silhouette scores above the average, indicating that each data point is closer to its own cluster center than to any other cluster. Cluster 1 has a larger size and a higher average silhouette score, suggesting it is more tightly grouped and distinct compared to the other clusters, although resulting in some imbalance in cluster sizes.

A total of 510 unique *ICD-10* codes were identified across all presentations. Although the elbow plot and gap statistic (Figures S1 and S2 in Multimedia Appendix 2) indicated that 2 clusters were the most parsimonious solution, a 4-cluster solution was ultimately selected to capture more clinically meaningful distinctions between groups. The 4-cluster solution had the highest gap value and achieved a moderately high silhouette score of 0.65, indicating well-separated and compact clusters (Figure 3). The Calinski-Harabasz Index for a 4-cluster solution was 3649.52, which was the highest when comparing solutions with 2-4 clusters. The Davies-Bouldin Index (0.46 for 4 clusters) also supported this choice, as it indicated relatively low within-cluster dispersion and high intercluster separation. While increasing the cluster number yielded higher Calinski-Harabasz values, the 4-cluster solution struck a balance between model complexity and interpretability (Table S2 in Multimedia Appendix 2). The resulting clusters comprised the following: cluster 1 with 262 *ICD-10* codes, cluster 2 with 104, cluster 3 with 66, and cluster 4 with 78 codes. The individual code prefixes in each cluster, with their associated admission risks, are reported in Table S3 in Multimedia Appendix 2. Sensitivity analysis was performed using *ICD-10* blocks (n=132; Table S5 in Multimedia Appendix 2) as a broader operationalization of diagnostic groupings for clustering. Clustering analysis suggested that a 2-cluster solution was the most parsimonious, while a 3-cluster solution offered more clinically meaningful distinctions (Figures S3-S5 in Multimedia Appendix 2). For the 2-cluster solution, the silhouette score, Calinski-Harabasz Index, and Davies-Bouldin Index were 0.71, 493.04, and 0.42, respectively; for 3 clusters, the values were 0.66, 616.12, and 0.46, respectively (Table S3 in Multimedia Appendix 2). Compared to clustering based on individual *ICD-10* codes, the block-based approach demonstrated substantially weaker separation, as reflected in the lower Calinski-Harabasz Index scores. These findings supported the use of raw *ICD-10* codes over broader *ICD-10* blocks.

### Association Between ED Presentation Characteristics and Cluster Membership

**Figure 4:**
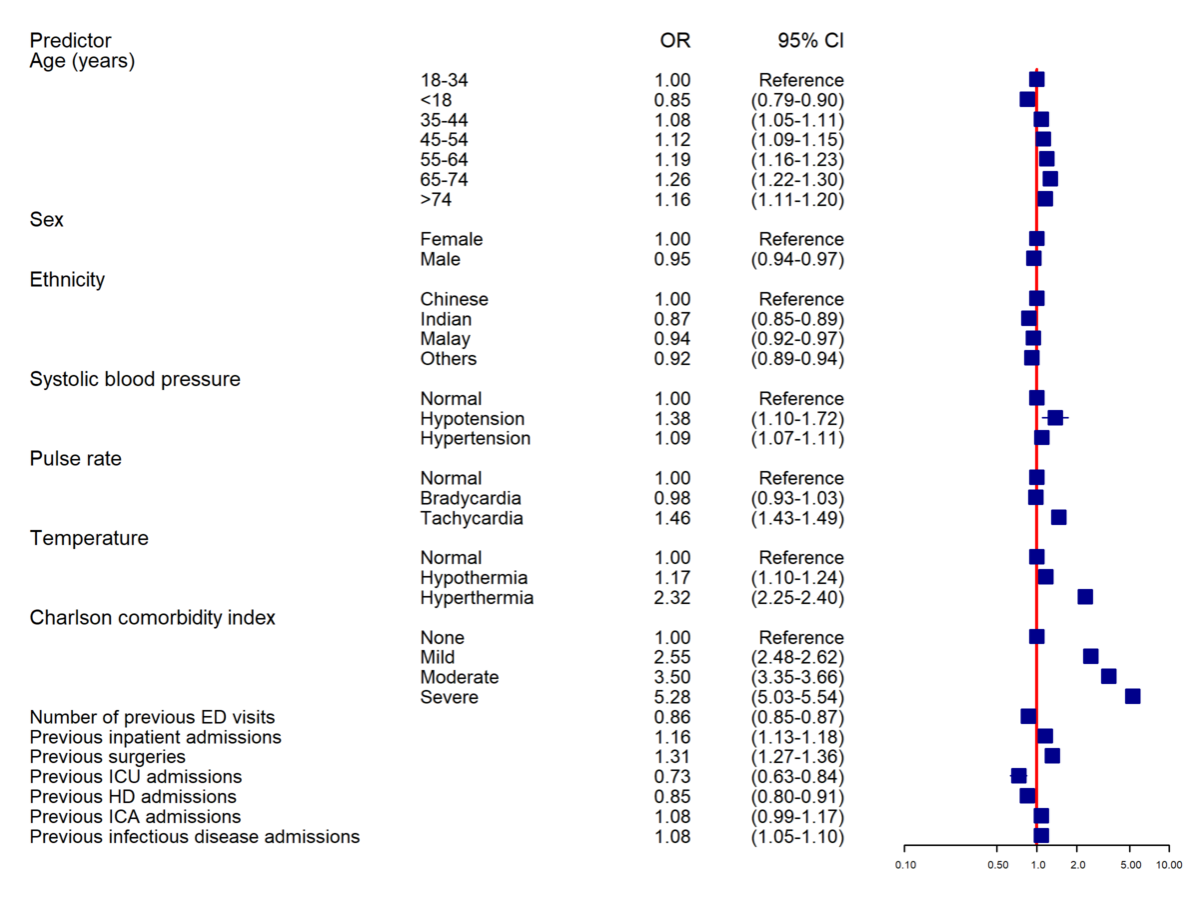
Ordinal logistic regression results: odds ratios (ORs) for cluster membership across predictors among ED presentations at a tertiary care hospital in Singapore (2016-2020). ED: emergency department; HD: high dependency; ICA: intermediate care area; ICU: intensive care unit.

The results of the ordinal logistic regression (Figure 4) indicated that the CCI was strongly associated with cluster membership, with mild (odds ratio [OR] 2.55, 95% CI 2.48-2.62), moderate (OR 3.50, 95% CI 3.35-3.66), and high (OR 5.28, 95% CI 5.03-5.54) comorbidity burden having increased odds of being admitted. Tachycardia (OR 1.46, 95% CI 1.43-1.49) and hyperthermia (OR 2.32, 95% CI 2.25-2.40) were also significantly associated with higher cluster membership. Variables such as recent surgery (OR 1.31, 95% CI 1.27-1.36) and inpatient admission (OR 1.16, 95% CI 1.13-1.18) increased the odds of being in a higher-risk cluster, while recent past ED visits (OR 0.86, 95% CI 0.85-0.87), intensive care unit admission (OR 0.85, 95% CI 0.80-0.91), and high dependency admission (OR 0.85, 95% CI 0.80-0.91) were associated with slightly decreased odds of presenting with severe conditions requiring admission.

### Identification of Dominant ICD-10 Blocks Across Clusters

**Figure 5:**
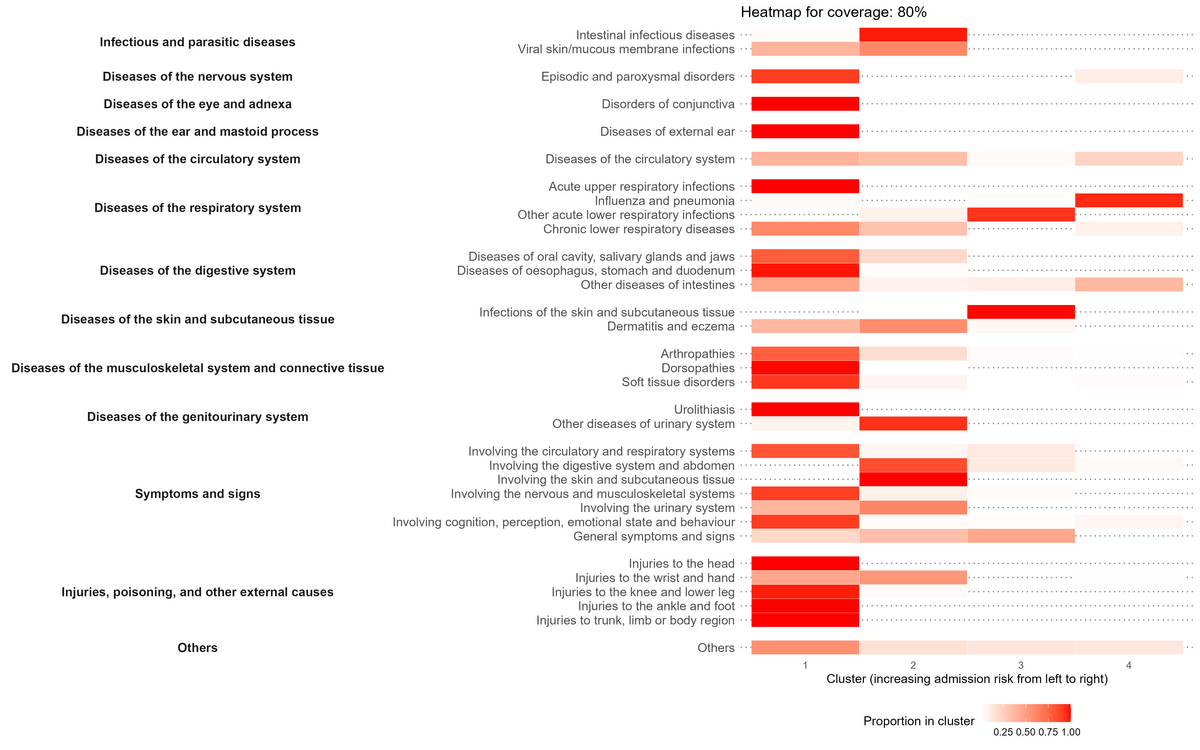
Heatmap of the top 80% coverage of unique ICD-10 blocks, according to cluster membership. Blocks are grouped according to broader ICD-10 categories (highlighted in bold). For each block, a higher proportion of individual codes belonging to the same cluster is indicated by a deeper red.

A total of 132 unique *ICD-10* blocks accounted for all *ICD-10* codes within the sample (Table S4 in Multimedia Appendix 2). When accounting for 90% of ED presentations, the dataset included 52 unique *ICD-10* blocks. Reducing the coverage to 80% decreased the number of unique blocks to 33, while further reductions resulted in 22 blocks for 70% coverage and 15 blocks for 60% coverage. The number of *ICD-10* blocks represented in cluster 4 was the lowest compared to cluster 1, with a decrease in the number of blocks across clusters at all coverage levels (Figure 5 and Figures S3-S5 in Multimedia Appendix 2). Sensitivity analyses included *ICD-10* codes with at least 10 presentations, resulting in the addition of 4 unique *ICD-10* blocks (Tables S6 and S7 in Multimedia Appendix 2). However, the number of unique blocks accounting for 60% to 90% of ED presentations remained unchanged. When analyzing *ICD-10* blocks with over 75% representation in a specific cluster, there were 55 *ICD-10* blocks in cluster 1, accounting for 116,183 (53.9%) presentations; and 18 *ICD-10* blocks in cluster 2, accounting for 24,056 (11.2%) presentations (Table S4 in Multimedia Appendix 2). When covering 80% of presentations, *ICD-10* blocks with more than 75% representation in the lowest-risk cluster (cluster 1) included 17 conditions (eg, external injuries to the body or soft tissue disorders), representing 94,330 presentations (43.8% of the total sample; Table 2). In the higher-risk clusters, this included those associated with systemic or infectious conditions, that is, influenza and pneumonia (*ICD* [*International Classification of Diseases*] J09-J18; cluster 4), other acute lower respiratory infections (*ICD* J20-J22), infections of the skin and subcutaneous tissue (*ICD* L00-L08; cluster 3), and intestinal infectious diseases (A00-A09; cluster 2).

**Table 2:** ICD-10^a^ blocks (n=21) with ≥75% representation in clusters 1 and 2.

Cluster and *ICD^b^* code grouping		*ICD-10* block
**Cluster 1**		
	G40-G47	Episodic and paroxysmal disorders
	H10-H13	Disorders of the conjunctiva
	H60-H62	Diseases of the external ear
	J00-J06	Acute upper respiratory infections
	K00-K14	Diseases of the oral cavity, salivary glands, and jaws
	K20-K31	Diseases of the esophagus, stomach and duodenum
	M00-M25	Arthropathies
	M40-M54	Dorsopathies
	M60-M79	Soft tissue disorders
	N20-N23	Urolithiasis
	R00-R09	Symptoms and signs involving the circulatory and respiratory systems
	R25-R29	Symptoms and signs involving the nervous and musculoskeletal systems
	R40-R46	Symptoms and signs involving cognition, perception, emotional state, and behavior
	S00-S09	Injuries to the head
	S80-S89	Injuries to the knee and lower leg
	S90-S99	Injuries to the ankle and foot
	T08-T14	Injuries to an unspecified part of the trunk, limb, or body region
**Cluster 2**		
	A00-A09	Intestinal infectious diseases
	N30-N39	Other diseases of the urinary system
	R10-R19	Symptoms and signs involving the digestive system and abdomen
	R20-R23	Symptoms and signs involving the skin and subcutaneous tissue

^a^ICD-10: International Statistical Classification of Diseases, Tenth Revision.

^b^ICD: International Classification of Diseases.

## Discussion

### Principal Findings

This study demonstrated the utility of clustering *ICD-10* codes as a novel method for stratifying admission outcomes in EDs among ambulatory patients. By consolidating diagnosis codes into clinically meaningful categories, this study sought to overcome the challenges of granular coding. A 4-cluster solution provided a balance between statistical robustness and clinical interpretability to identify meaningful distinctions in hospital admission risks. Cluster 1 represented presentations that require minimal intervention or monitoring, such as musculoskeletal conditions (eg, dorsalgia or spondylosis) and skin conditions (eg, urticaria). However, cluster 4 identified higher risk presentations, despite their stable and ambulatory classification at triage. Although this cluster consists of a minority of the total sample, its high admission risk underscores the need for a triage process with greater sensitivity for high-risk presentations, even at the expense of some false positives. The gradient of risk also showed that admission risk patterns were nonlinear in distribution, with cluster 1 having 4.7% risk, and cluster 2 increasing to nearly 20% admission risk, and cluster 4 characterized by a 78% admission risk. Cluster 1 had the largest cluster size, reflecting a broader range of conditions and greater heterogeneity. However, this does not compromise the clinical utility of the classification system. All *ICD-10* diagnoses were retained and systematically grouped into standardized *ICD-10* blocks, ensuring that no subgroups were omitted from the analysis.

Logistic regression analysis identified key predictors of cluster membership, including CCI, vital sign abnormalities (hyperthermia or tachycardia), and recent surgical history. Presentations with moderate and severe comorbidities were significantly more likely to belong to higher-risk clusters, which is consistent with prior studies that emphasize the predictive value of CCI for hospitalization and poorer outcomes [39-41]. Our study’s findings also align with other studies that show that vital sign abnormalities, such as hyperthermia and tachycardia, were associated with higher admission risks [42]. In contrast to other studies that have linked frequent ED visits with higher admission risk [43,44], our analysis did not find a strong association between prior ED attendance and being in a higher-risk cluster. This may be due to the inclusion of lower acuity (P3 and P4) cases in our cohort, where frequent ED use may not necessarily indicate severe or worsening health conditions. Perhaps surprisingly, previous ICU and high dependency attendances in the last 6 months were associated with lower odds of being in a higher admission risk cluster. This phenomenon may be explained by the “depletion of susceptibles” effect, where a harmful exposure appears protective due to selective survival [45]. It is possible that individuals at highest risk did not survive over time, leaving behind a subset of relatively healthier ICU and high dependency survivors who are less likely to require critical care upon representation. Another possible explanation is that although these patients have increased generalized vulnerability following hospitalization [46], they may receive closer monitoring through structured care pathways and multidisciplinary follow-up systems [47]. This could reduce the likelihood of delayed presentation and result in a lower probability of presenting with more acute conditions at the ED [48,49].

A total of 132 unique *ICD-10* blocks were identified within the sample, but this number may be too granular to screen for ACSPs. However, when coverage was reduced to 90% of ED presentations, the number of *ICD-10* blocks decreased to a more manageable 52, and further reduction to 80% coverage reduced it to 33 blocks. At the 80% cutoff, the lowest-risk cluster (cluster 1) consisted of 27 *ICD-10* blocks of any proportion, with 17 blocks representing ≥75% of the cases in this group, accounting for 43.8% (n=94,330) of the total sample. These predominantly included conditions such as musculoskeletal or connective tissue disorders (eg, arthropathies, soft tissue disorders, or dorsopathies), episodic and paroxysmal disorders, upper respiratory infections, disorders of the ear and conjunctiva, and injuries primarily to the head and lower limbs. *ICD-10* blocks in cluster 2 primarily included presentations with gastrointestinal, urinary, and skin-related symptoms. Common diagnoses included infectious gastroenteritis (A09), abdominal and pelvic pain (R10), nausea and vomiting (R11), and localized skin rash and swelling (R21 or R22). The large proportion of low-risk presentations in cluster 1, along with identifying presentations with high-representation *ICD-10* blocks in lower admission risk clusters, suggests that some of these cases could be triaged earlier or even virtually via telemedicine. This offers opportunities to redirect patients to ACSPs, reducing unnecessary ED visits and optimizing resource allocation [19,50].

The strength of this study lies in identifying categories of diagnoses that can be considered for ACSPs within a worldwide recognized and highly granular classification system. Understanding the distribution of lower-risk conditions enables optimizing resource allocation such as establishing urgent care centers and primary care centers to be equipped to manage conditions such as musculoskeletal conditions such as arthropathies and dorsopathies, and minor external injuries to the upper and lower limbs, which were predominantly grouped into cluster 1 with the lowest hospital admission risk. It is important to distinguish by identifying nonurgent cases from addressing ED overcrowding, which is influenced by more complex factors such as overburdened inpatient facilities, staffing shortages, and aging populations [3,51]. Rather, the findings suggest that certain conditions could be potentially treated outside the ED, which is intended for rapid, unscheduled care of urgent cases. Managing stable cases in primary care can also foster stronger patient-provider relationships [52].

This study has several limitations. First, cluster analysis lacks a perfectly objective method for determining the number of clusters. While statistical criteria such as the gap statistic and elbow plot can guide the process, clusters must also have a balance between parsimony and clinical interpretability [53]. Additionally, admission risk was determined solely based on *ICD-10* diagnoses, without accounting for other potential confounders such as access to outpatient specialty care, or social determinants of health such as deprivation index and population dependency ratio [54], which were unavailable in this dataset. Electronic health data from hospital systems outside the SingHealth cluster were not available for this study, and may have resulted in incomplete information on prior admissions. However, information on patients’ comorbidities would typically be accessible on the National Electronic Health Record, which consolidates data across all public clusters and private health care providers, allowing clinicians to review relevant medical history at the ED, minimizing information gaps. *ICD-10* diagnoses may also not reflect precise clinical diagnoses, with conditions such as heart failure being inaccurately coded due to overlap in symptoms and variability in documentation practices [55]. Furthermore, due to the lack of access to unstructured data, such as discharge summaries or case notes, it was not possible to further investigate the decision-making process in assigning diagnosis codes or the decisions behind admitting patients [56]. The COVID-19 pandemic may also have influenced ED use patterns with shifts in case mix, such as an increase in respiratory presentations [57]. However, as our study focuses on admission risk stratification and captures a diverse range of *ICD-10* codes, the findings remain relevant across different periods. Lastly, this is a single-center study conducted at a tertiary academic center, which may have received patients who are demographically different from the broader population. While differences may be limited given Singapore’s status as a small city-state, variations in clinical care practices and hospital infrastructure could still affect the generalizability of the findings. Nonetheless, the results should be externally validated in diverse health care systems for broader applicability.

EDs worldwide use various triage systems to prioritize patient care based on urgency. These include the Manchester Triage System and the Emergency Severity Index, both of which use criteria such as presenting symptoms, vital signs, and anticipated resource needs to classify patients into 5 levels of acuity [58]. In Singapore, public hospitals use the Patient Acuity Category Scale, a symptom-based approach that incorporates presenting complaints and objective assessments such as vital signs and the Glasgow Coma Scale to rapidly identify and categorize patients into 4 priority levels. This study introduces a clustering methodology that may complement existing triage systems by stratifying ED presentations according to admission risk. Notably, this study’s findings reveal that a small but significant proportion of cases initially classified as minor or nonemergencies (P3/P4) were at high risk of admission. This suggests that standard triage protocols may benefit from incorporating additional predictors such as patient history, particularly recent surgery and prior inpatient admissions, both identified as indicators of higher admission risk in this study. Integrating these may enhance the predictive accuracy of triage assessments, leading to optimized patient care within the ED.

Future studies could explore ways to refine the use of *ICD* codes for decision support in triaging cases for ACSPs at a population health level. One ongoing challenge in triaging patients is that there is limited concordance between presenting complaints and actual clinical diagnosis, highlighting the need for more comprehensive data integration [59]. In this study, diagnosis codes were assigned based on their final disposition, suggesting that triaging of nonurgent cases suitable for alternative care needs to be identified earlier in the PEC pathway.

At the same time, the triage system must maintain high sensitivity, as this study’s findings indicate that a minority of ED attendances were at high risk of admission despite being classified as minor or nonemergency (P3/P4) cases. Integrating predictive modelling with patient history, comorbidities, and real-time clinical data may improve triage accuracy further upstream to stratify patient acuity. This includes expanding data collection efforts and developing ED administration tools to integrate clinically relevant predictors into the triage process.

These findings serve as an initial step in identifying potential patient groups suitable for ACSPs. We intend to follow up with a Delphi process to establish expert consensus on defining thresholds to determine which lower admission risk groups can be safely redirected. This will involve engaging key stakeholders such as ED physicians, primary care physicians, urgent care providers, and health care administrators to identify practical implementation strategies. The goal will be to design and pilot interventional ACSP models for implementation and scaling.

### Conclusion

This study is a first step toward demonstrating the potential utility of clustering *ICD-10* codes as a novel approach for stratifying admission outcomes in the ED among ambulatory patients. Admission risk patterns were nonlinear, and applying thresholds to group *ICD-10* blocks based on the proportion of ED presentations they represent may provide a balance by maintaining adequate representation of admission risk groups without losing too much granularity. Future work should focus on more accurately identifying these cases suitable for referral to ACSPs.

## Data Availability

The data that support the findings of this study are available from the Pre-hospital & Emergency Research Centre, Duke-NUS Medical School. Restrictions apply to the availability of these data, which were used under the license for this study. Data are available from the authors upon reasonable request, with the permission of Duke-NUS Medical School.

## Appendix

Multimedia Appendix 1 Visual abstract.

Multimedia Appendix 2 Additional material.

Multimedia Appendix 3 Transcript.

## Abbreviations

ACSP: alternative care service pathway
CCI: Charlson Comorbidity Index
ED: emergency department
ICD: International Classification of Diseases
ICD-10: International Statistical Classification of Diseases, Tenth Revision
ICD-10-CM: International Classification of Diseases, Tenth Revision, Clinical Modification
OR: odds ratio
PEC: prehospital emergency care
RECORD: Reporting of Studies Conducted Using Observational Routinely-Collected Data
SingHealth: Singapore Health Services
SNOMED-CT: Systematized Nomenclature of Medicine Clinical Terms
