# Supplementary material for: Pragmatic Risk Stratification Method to Identify Emergency Department Presentations for Alternative Care Service Pathways: Registry-Based Retrospective Study Over 5 Years
Source: J Med Internet Res. 2025 May 12;27:e73758. doi: 10.2196/73758 (PMC12107196; doi:10.2196/73758)
Supplement: Multimedia Appendix 1 [file jmir_v27i1e73758_app1.pdf]

Grouping Diagnostic codes in Emergency Department by Clustering approach

Background

Non-urgent visits to the emergency department (ED) lead to overcrowding, and redirecting non-urgent cases to alternative care services (e.g., primary care) may lead to better allocation of resources

Aim

To cluster diagnosis codes based on hospital admission risk to develop an exploratory classification of groups potentially suitable for alternative care services

Method

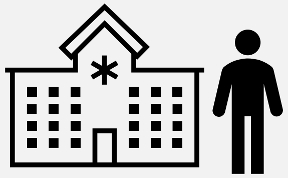

A retrospective study
All visits to the ED of a tertiary care hospital with lower priority
(n =215,477)

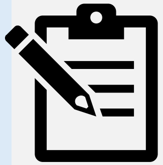

ICD-10 Diagnostic codes in ED

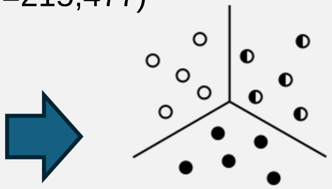

K-means Clustering

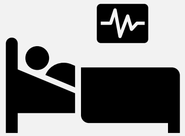

Evaluate the membership and admission risk of the groups

Results

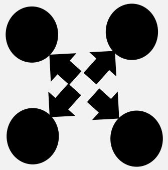

Each diagnosis code arranged according to 4 different clusters

Cluster 1
Lowest admission risk (4.7%)
Characterized by minor injuries, soft tissue disorders, etc.

Cluster 4
Highest admission risk (78%)
Characterized by pneumonia, infections of the skin and subcutaneous tissue.

Summary

This study demonstrates the potential of clustering diagnosis codes to stratify admission risk among ambulatory patients, highlighting non-linear risk patterns and the need for balanced grouping approaches

Tay J, Okada Y, Nadarajan G, Siddiqui F, Barry T, Ong M. Pragmatic risk stratification method to identify emergency department presentations for alternative care service pathways: a register-based retrospective study over 5 years
