# Supplementary material for: Pragmatic Risk Stratification Method to Identify Emergency Department Presentations for Alternative Care Service Pathways: Registry-Based Retrospective Study Over 5 Years
Source: J Med Internet Res. 2025 May 12;27:e73758. doi: 10.2196/73758 (PMC12107196; doi:10.2196/73758)
Supplement: Multimedia Appendix 2 [file jmir_v27i1e73758_app2.docx]

# **Content of the Additional Material**

Page 2 **Figure S1.** Elbow plot for K-means clustering.

Page 2 **Figure S2.** Gap statistic using K-means clustering.

Page 3 **Figure S3.** Silhouette plot for K-means clustering based on admission risk grouped by ICD-10 blocks.

Page 3 **Figure S4.** Elbow plot for K-means clustering based on admission risk grouped by ICD-10 blocks.

Page 4 **Figure S5.** Gap statistic using K-means clustering based on admission risk grouped by ICD-10 blocks.

Page 4 **Figure S6.** Heatmap of unique ICD-10 blocks by cluster: Top 90% Coverage.

Page 5 **Figure S7.** Heatmap of unique ICD-10 blocks by cluster: Top 70% Coverage.

Page 5 **Figure S8.** Heatmap of unique ICD-10 blocks by cluster: Top 60% Coverage.

Page 6-12 **Table S1.** SNOMED CT codes that could not be mapped to ICD-10 codes.

Page 13 **Table S2.** Cluster evaluation metrics (2-10 clusters).

Page 13 **Table S3.** Cluster evaluation metrics (2-10 clusters), grouped by ICD-10 blocks.

Page 14-30 **Table S4.** ICD-code prefixes according to cluster identity.

Page 31-51 **Table S5.** ICD-code prefixes stratified by ICD-code blocks.

Page 52-65 **Table S6.** Excluded ICD-codes.

Page 66-67 **Table S7.** ED presentation characteristics, including ICD-10 codes with at least 10 presentations.

Page 67 **Table S8.** Number ICD-10 blocks according to different cumulative thresholds, when including ICD-10 codes with at least 10 presentations.

**Figure S1.** Elbow plot for K-means clustering.


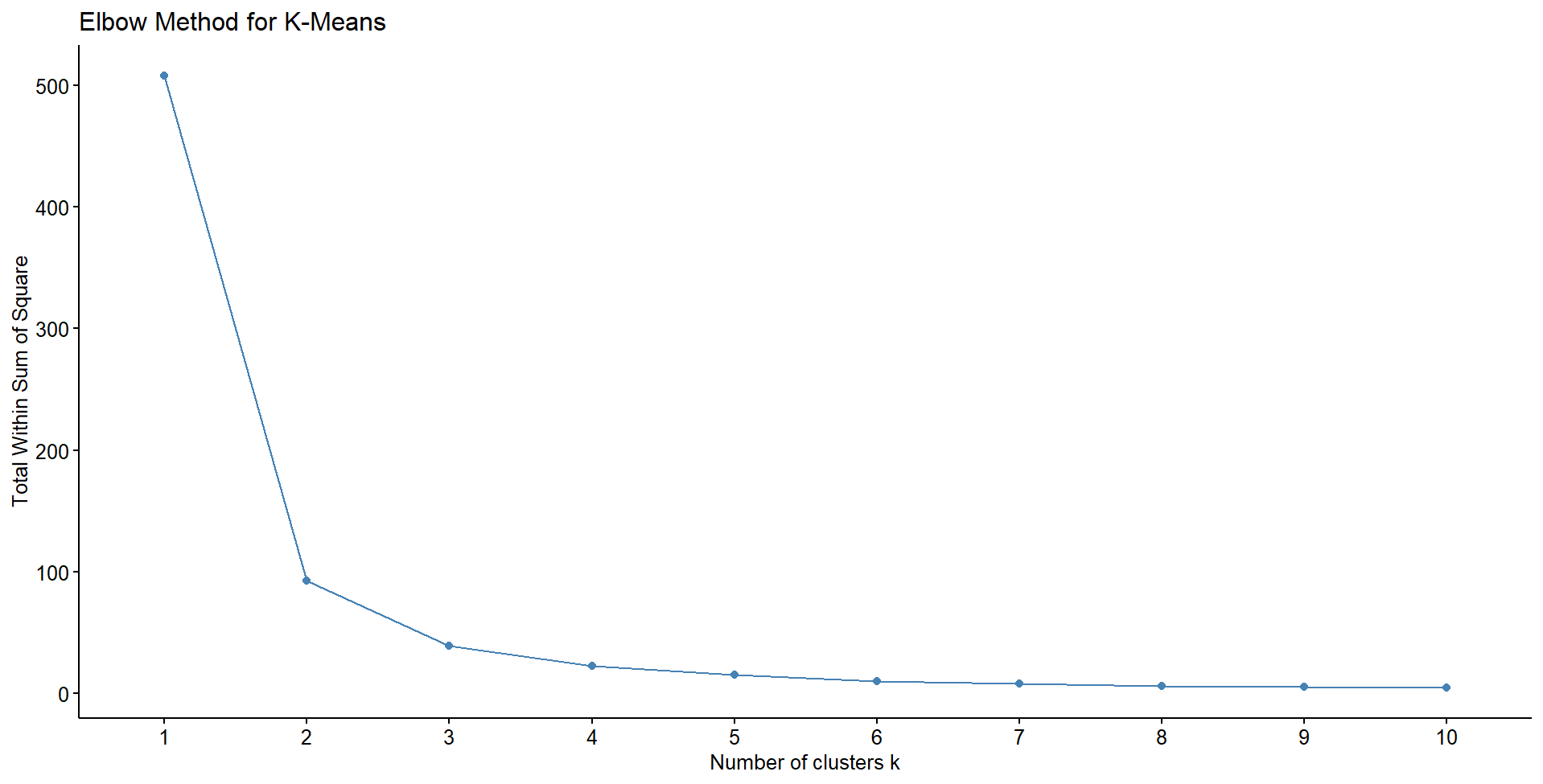


**Figure S2.** Gap statistic using K-means clustering.


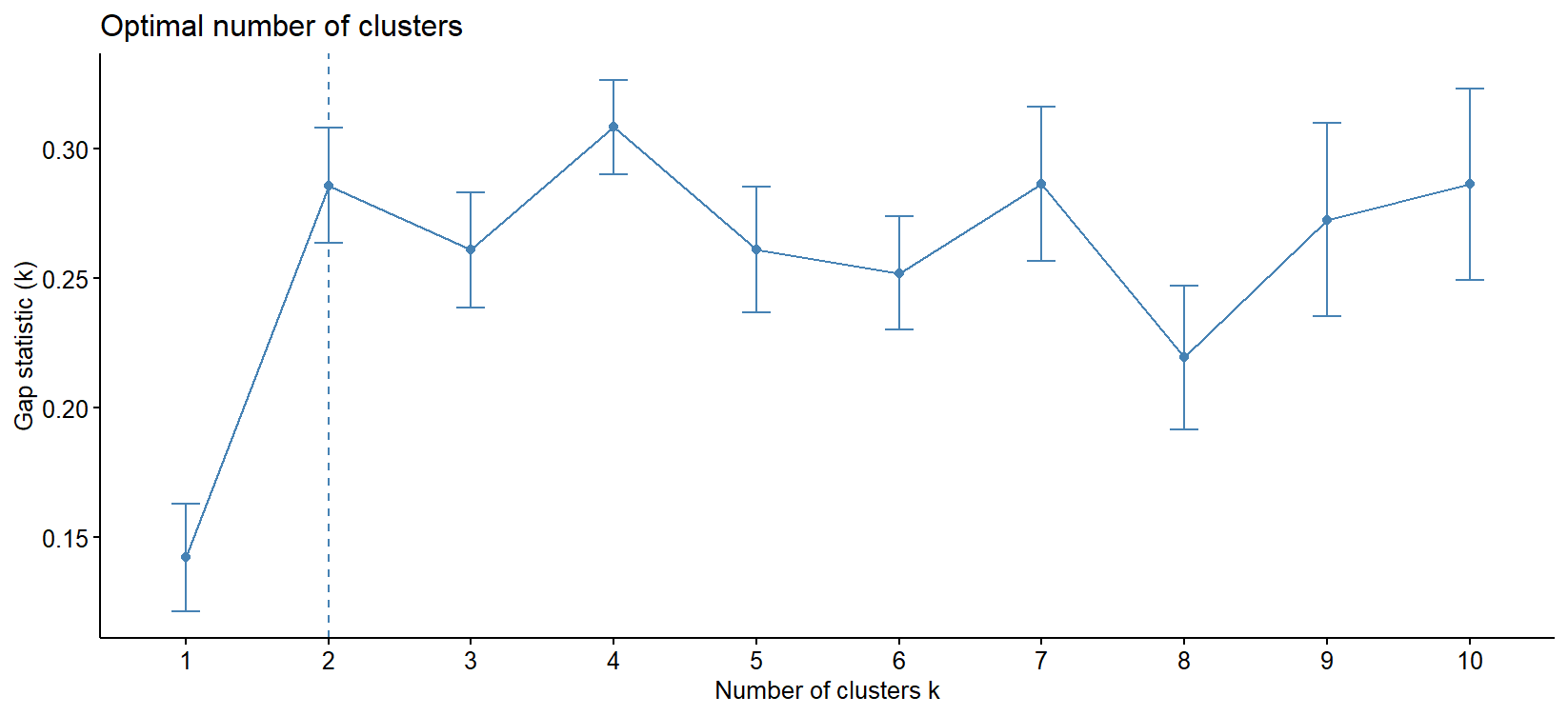


**Figure S3.** Silhouette plot for K-means clustering based on admission risk grouped by ICD-10 blocks.

**
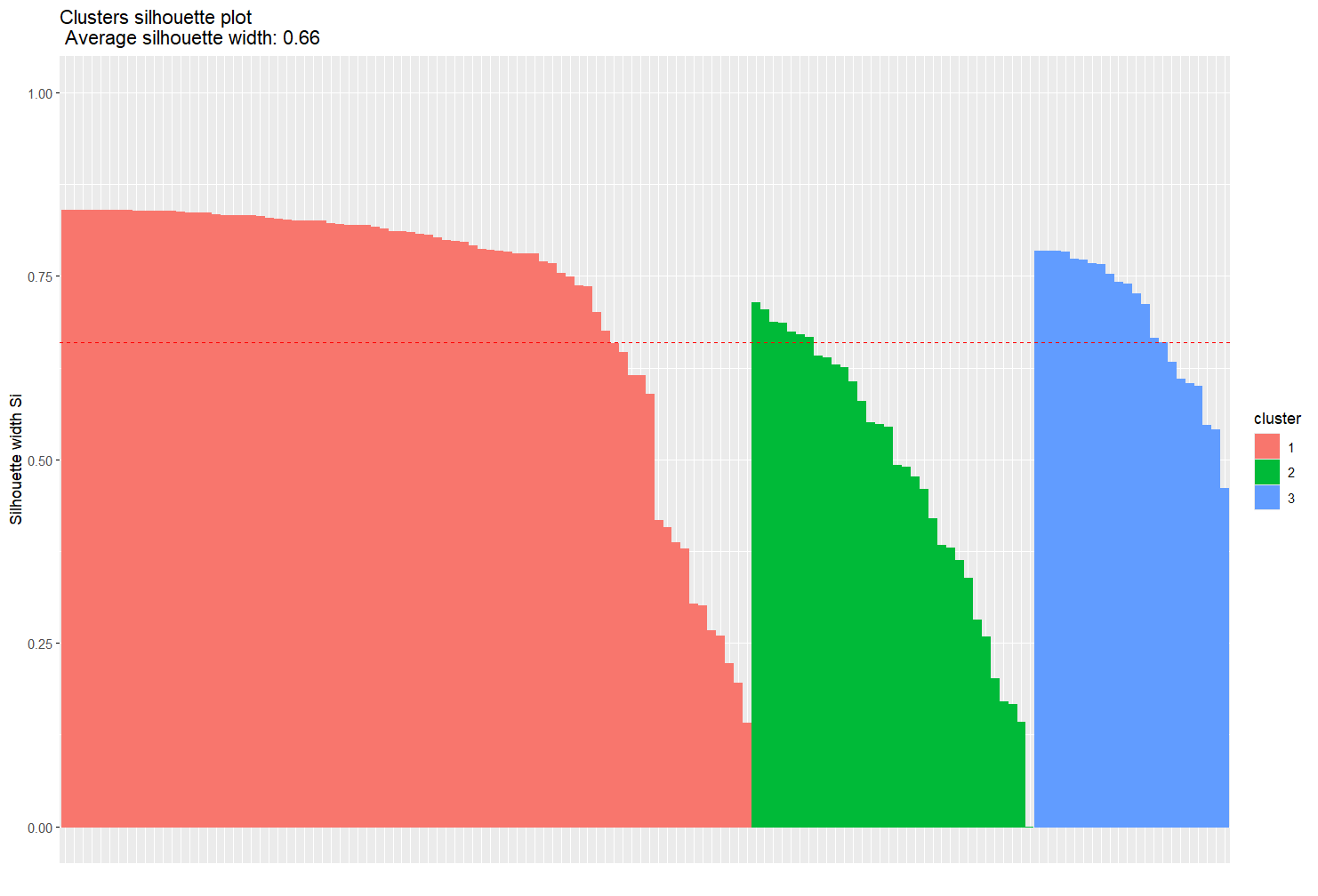
**

**Figure S4.** Elbow plot for K-means clustering based on admission risk grouped by ICD-10 blocks.

**
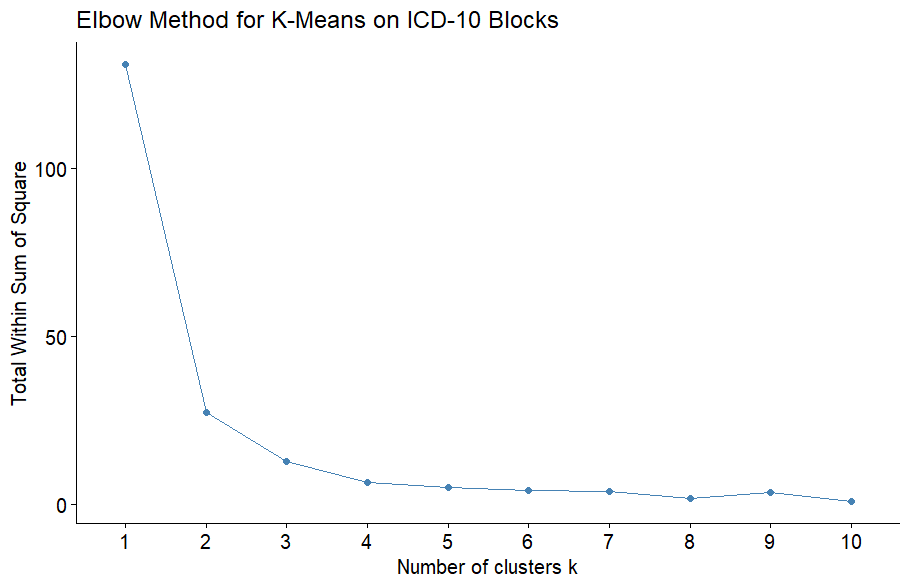
**

**Figure S5.** Gap statistic using K-means clustering based on admission risk grouped by ICD-10 blocks.

**
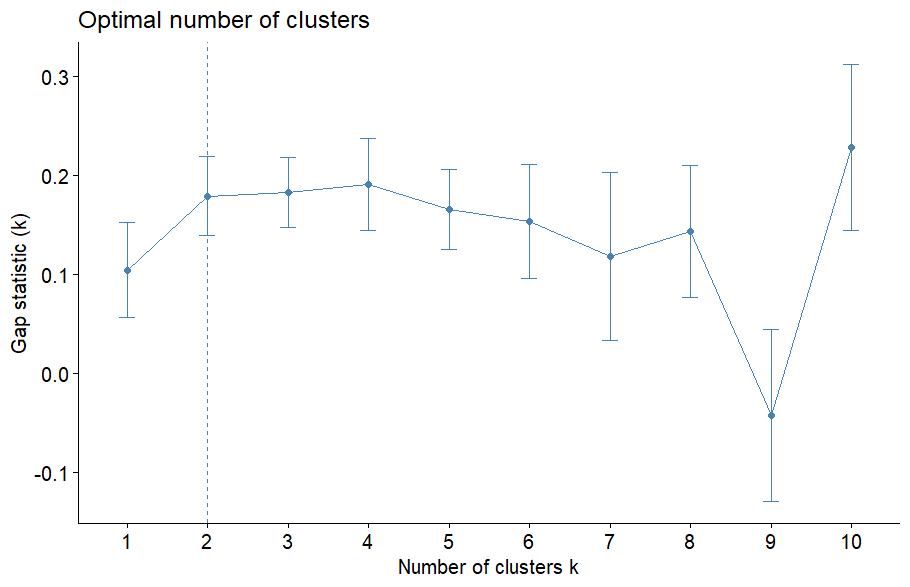
**

**Figure S6.** Heatmap of unique ICD-10 blocks by cluster: Top 90% Coverage.

**
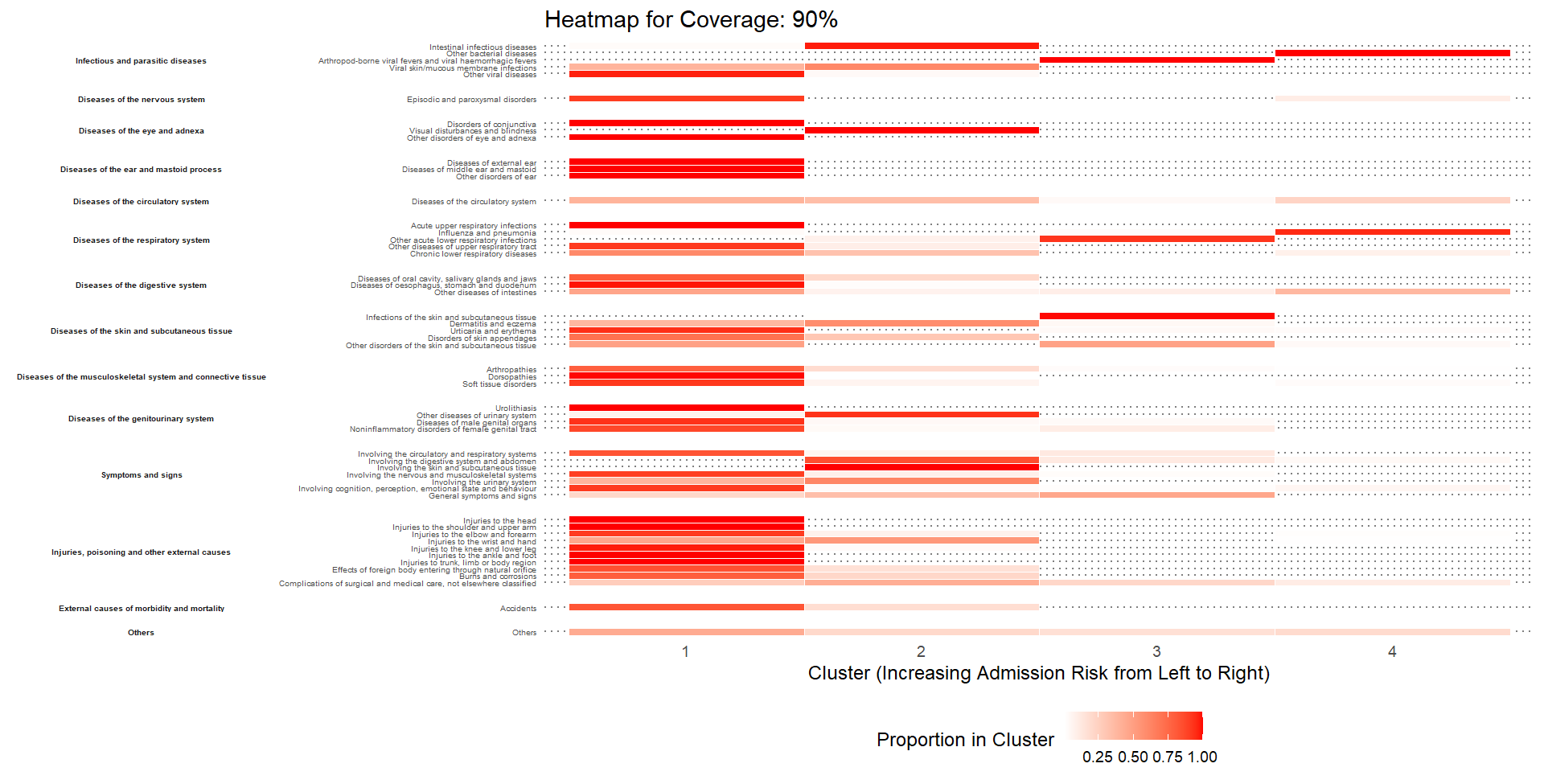
**

**Figure S7.** Heatmap of unique ICD-10 blocks by cluster: Top 70% Coverage.

**
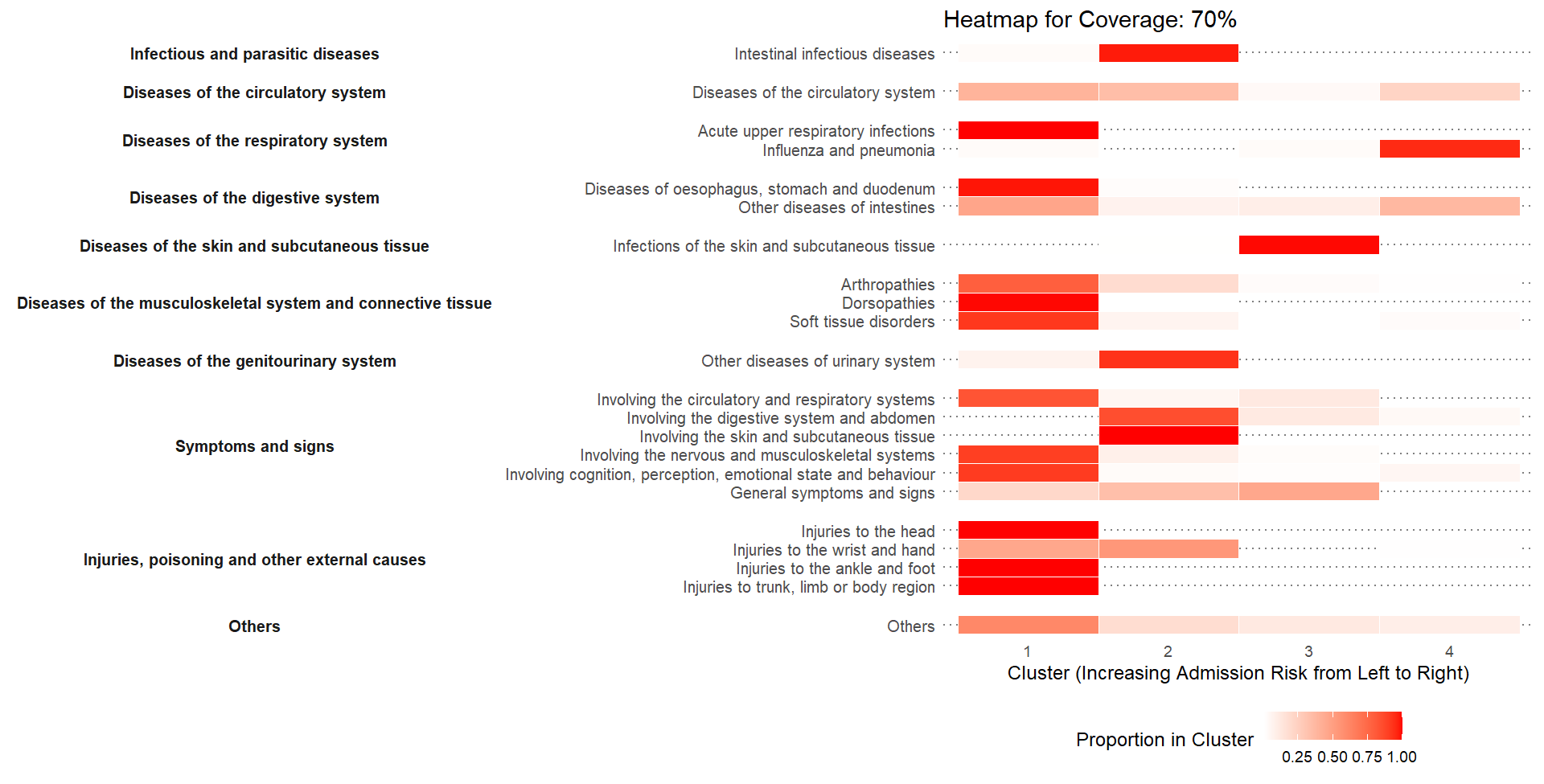
**

**Figure S8.** Heatmap of unique ICD-10 blocks by cluster: Top 60% Coverage.


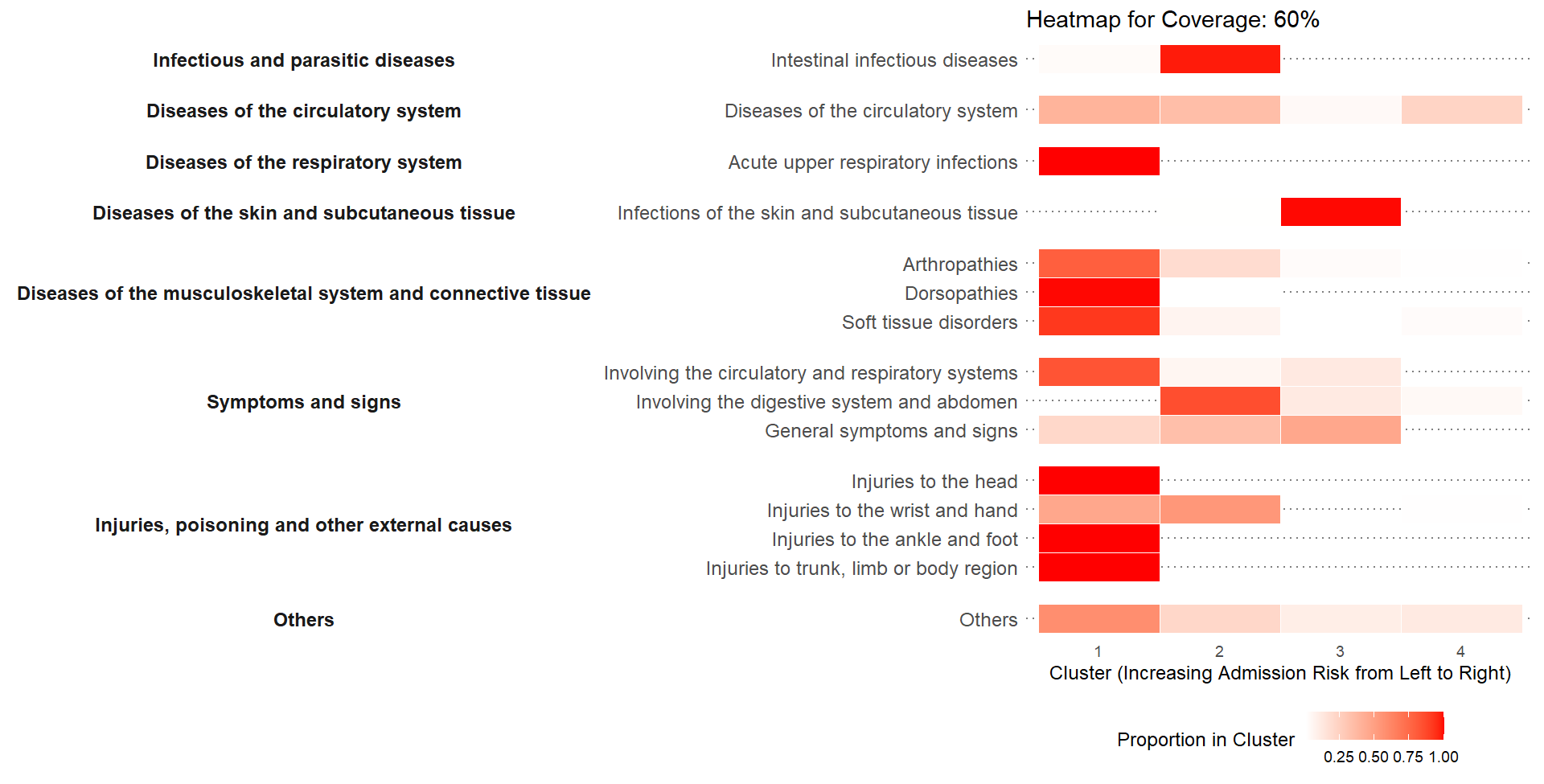


**Table S1.** SNOMED CT codes that could not be mapped to ICD-10 codes.

| SNOMED CT Diagnosis | Count | Frequency (%) |
| --- | --- | --- |
| Lump | 216 | 12.7 |
| Transaminitis | 160 | 9.4 |
| Acute kidney injury | 147 | 8.7 |
| Abrasion and/or friction burn of multiple sites | 88 | 5.2 |
| Cyst | 72 | 4.2 |
| Eye symptom | 58 | 3.4 |
| Past medical history | 58 | 3.4 |
| Foreign body - finger | 54 | 3.2 |
| Mass | 44 | 2.6 |
| Varicella-zoster virus infection | 38 | 2.2 |
| Normal pregnancy | 34 | 2.0 |
| Abrasion and/or friction burn of multiple sites | 30 | 1.8 |
| First pregnancy | 26 | 1.5 |
| Wound seroma | 26 | 1.5 |
| Contraception | 25 | 1.5 |
| Tear meniscus | 23 | 1.4 |
| Ingestion of foreign material | 22 | 1.3 |
| Vision disorder | 19 | 1.1 |
| Compression fracture | 18 | 1.1 |
| Fall risk | 17 | 1.0 |
| Abrasion and/or friction burn | 16 | 0.9 |
| Vaccination required | 15 | 0.9 |
| Vasovagal symptom | 15 | 0.9 |
| Past medical history | 12 | 0.7 |
| Stoma finding | 12 | 0.7 |
| Well child | 12 | 0.7 |
| Liver enzymes abnormal | 11 | 0.6 |
| Poor hypertension control | 11 | 0.6 |
| Closed fracture of one or more phalanges of hand | 10 | 0.6 |
| Raised transaminase | 10 | 0.6 |
| Suspected malignancy | 9 | 0.5 |
| Warfarin monitoring status | 9 | 0.5 |
| Diabetic ketoacidosis | 8 | 0.5 |
| Disease caused by 2019 novel coronavirus | 8 | 0.5 |
| Disorder of vision | 8 | 0.5 |
| Newly diagnosed diabetes | 8 | 0.5 |
| No carer | 8 | 0.5 |
| Old healed fracture of bone | 8 | 0.5 |
| Physical health problems | 8 | 0.5 |
| Disorder of stoma | 7 | 0.4 |
| Functional disorder | 7 | 0.4 |
| International Normal Ratio abnormal | 7 | 0.4 |
| Laboratory test result abnormal | 7 | 0.4 |
| Chronic kidney disease | 6 | 0.4 |
| Head and neck mass | 6 | 0.4 |
| Normal behaviour | 6 | 0.4 |
| Suspected disease caused by 2019 novel coronavirus | 6 | 0.4 |
| Vague symptoms | 6 | 0.4 |
| Breast finding | 5 | 0.3 |
| Burns classified according to percentage of body surface involved | 5 | 0.3 |
| Complication | 5 | 0.3 |
| Family social history | 5 | 0.3 |
| Hearing disorder | 5 | 0.3 |
| Middle East respiratory syndrome coronavirus (MERS-CoV) | 5 | 0.3 |
| Oral contraception | 5 | 0.3 |
| Rotator cuff shoulder syndrome and allied disorders | 5 | 0.3 |
| Back injury | 4 | 0.2 |
| Cellulitis | 4 | 0.2 |
| Elevated transaminase | 4 | 0.2 |
| General problem and/or complaint | 4 | 0.2 |
| Genital finding | 4 | 0.2 |
| Mucositis | 4 | 0.2 |
| Normal behavior | 4 | 0.2 |
| Oral contraceptive | 4 | 0.2 |
| Sprain shoulder and upper arm | 4 | 0.2 |
| Superficial partial thickness burn | 4 | 0.2 |
| Symptom of lower limb | 4 | 0.2 |
| Symptom of skin and integumentary tissue | 4 | 0.2 |
| Complaint of lower limb symptom | 3 | 0.2 |
| Carbuncle/boil | 3 | 0.2 |
| Disease suspected | 3 | 0.2 |
| Disorder of head | 3 | 0.2 |
| Hematoma | 3 | 0.2 |
| Penile foreign body | 3 | 0.2 |
| Piles - obstetric | 3 | 0.2 |
| Pregnancy test indeterminate | 3 | 0.2 |
| Problem, abnormal examination | 3 | 0.2 |
| Sprain of knee and leg | 3 | 0.2 |
| Sprain of shoulder and upper arm | 3 | 0.2 |
| Symptom | 3 | 0.2 |
| Traumatic or non-traumatic rupture of tendon | 3 | 0.2 |
| Urine odour | 3 | 0.2 |
| Abdominal infection | 2 | 0.1 |
| Abnormal blood pressure | 2 | 0.1 |
| At risk for falls | 2 | 0.1 |
| At risk of falls | 2 | 0.1 |
| Bursitis - ankle/foot | 2 | 0.1 |
| Cannot describe symptoms | 2 | 0.1 |
| Cervical, vaginal and vulval inflammatory diseases | 2 | 0.1 |
| Change in voice | 2 | 0.1 |
| Disorder of hand | 2 | 0.1 |
| Diverticulitis | 2 | 0.1 |
| Epicondylitis | 2 | 0.1 |
| Feels the cold | 2 | 0.1 |
| Fertility problem | 2 | 0.1 |
| Functional finding | 2 | 0.1 |
| Infrequent urination | 2 | 0.1 |
| Intolerant of cold | 2 | 0.1 |
| Latent tuberculosis | 2 | 0.1 |
| Mass in head or neck | 2 | 0.1 |
| Menopause symptoms present | 2 | 0.1 |
| Musculoskeletal disorder of the neck | 2 | 0.1 |
| Normal social conduct | 2 | 0.1 |
| Open fracture of one or more phalanges of hand | 2 | 0.1 |
| Pelvic abscess | 2 | 0.1 |
| Pill - oral contraception | 2 | 0.1 |
| Problem related to psychosocial circumstance | 2 | 0.1 |
| Radiology result abnormal | 2 | 0.1 |
| Sensitive to cold | 2 | 0.1 |
| Substance abuse | 2 | 0.1 |
| Suspected infectious disease | 2 | 0.1 |
| Suspected neurological disease | 2 | 0.1 |
| Urine finding | 2 | 0.1 |
| Abnormal glucose level | 1 | 0.1 |
| Abrasion | 1 | 0.1 |
| Abrasion and/or friction burn of forearm with infection | 1 | 0.1 |
| Abrasion and/or friction burn of multiple sites, infected | 1 | 0.1 |
| Abrasion and/or friction burn of hand, infected | 1 | 0.1 |
| Abrasion and/or friction burn of lower limb, infected | 1 | 0.1 |
| Abrasion or friction burn of foot and toe, infected | 1 | 0.1 |
| Abrasion or friction burn of hand, infected | 1 | 0.1 |
| Abscess | 1 | 0.1 |
| Acute bacterial otitis externa | 1 | 0.1 |
| Acute lymphoblastic leukemia | 1 | 0.1 |
| Acute musculoskeletal disease | 1 | 0.1 |
| Allergy | 1 | 0.1 |
| Bartter's syndrome | 1 | 0.1 |
| Birth control | 1 | 0.1 |
| Blindness and/or vision impairment level | 1 | 0.1 |
| Blood transfusion observation | 1 | 0.1 |
| Breast observations | 1 | 0.1 |
| C-reactive protein abnormal | 1 | 0.1 |
| Caffeine-related disorder | 1 | 0.1 |
| Cataract | 1 | 0.1 |
| Chronic schizophrenia | 1 | 0.1 |
| Closed compression fracture | 1 | 0.1 |
| Closed contusion | 1 | 0.1 |
| Closed fracture pelvis, coccyx | 1 | 0.1 |
| Cold sensitivity | 1 | 0.1 |
| Complication associated with orthopaedic device | 1 | 0.1 |
| Concussive injury | 1 | 0.1 |
| Digital nerve injury | 1 | 0.1 |
| Discussed with carer | 1 | 0.1 |
| Disorder of foot | 1 | 0.1 |
| Disorder of hair | 1 | 0.1 |
| Ear, Nose, Throat (ENT) Symptoms | 1 | 0.1 |
| Enzyme level - finding | 1 | 0.1 |
| Foreign body in penis | 1 | 0.1 |
| Fracture sacrum/coccyx - no cord lesion | 1 | 0.1 |
| Fracture, open | 1 | 0.1 |
| Functional disturbance | 1 | 0.1 |
| Functional state after urinary stone passed | 1 | 0.1 |
| Fundic gland polyp | 1 | 0.1 |
| Granulomatous disease | 1 | 0.1 |
| Grief | 1 | 0.1 |
| Hepatocellular carcinoma | 1 | 0.1 |
| Hypercoagulable state | 1 | 0.1 |
| Hyperosmolar hyperglycaemia state | 1 | 0.1 |
| International normal ratio normal | 1 | 0.1 |
| Illness | 1 | 0.1 |
| Infected abrasion | 1 | 0.1 |
| Infection of abdomen | 1 | 0.1 |
| Injury of digital nerve | 1 | 0.1 |
| Laceration | 1 | 0.1 |
| Leucoplakia | 1 | 0.1 |
| Liver cell carcinoma | 1 | 0.1 |
| Lower limb length difference | 1 | 0.1 |
| Mass of body structure | 1 | 0.1 |
| Motor vehicle accident | 1 | 0.1 |
| Multiple open wounds | 1 | 0.1 |
| Nasal dryness | 1 | 0.1 |
| Norovirus Genogroup I and Genogroup II detected | 1 | 0.1 |
| On examination - cushingoid facies | 1 | 0.1 |
| Oral contraceptive | 1 | 0.1 |
| Open wound of hip and thigh | 1 | 0.1 |
| Osteophyte | 1 | 0.1 |
| Postprocedural state finding | 1 | 0.1 |
| Procidentia | 1 | 0.1 |
| Serum tumour marker level | 1 | 0.1 |
| Skin irritation | 1 | 0.1 |
| Social and personal history finding | 1 | 0.1 |
| Stoma observations | 1 | 0.1 |
| Support system deficit | 1 | 0.1 |
| Suspected Severe Acute Respiratory Syndrome (SARS) | 1 | 0.1 |
| Teen-age | 1 | 0.1 |

Abbreviations: SNOMED CT, Systematized Nomenclature of Medicine Clinical Terms.

**Table S2.** Cluster evaluation metrics (2-10 clusters).

| **Number of Clusters** | **Average Silhouette Score** | **Calinski-Harabasz Index** | **Davies-Bouldin Index** |
| --- | --- | --- | --- |
| 2 | 0.75 | 2,271.33 | 0.39 |
| 3 | 0.71 | 3,008.93 | 0.43 |
| 4 | 0.65 | 3,649.52 | 0.47 |
| 5 | 0.62 | 4,117.38 | 0.51 |
| 6 | 0.62 | 4,949.25 | 0.50 |
| 7 | 0.58 | 5,333.60 | 0.52 |
| 8 | 0.58 | 6,022.26 | 0.51 |
| 9 | 0.58 | 7,027.11 | 0.49 |
| 10 | 0.58 | 7,927.43 | 0.49 |

**Table S3.** Cluster evaluation metrics (2-10 clusters), grouped by ICD-10 blocks.

| **Cluster Evaluation Metrics** | | | |
| --- | --- | --- | --- |
| **Number of Clusters** | **Average Silhouette Score** | **Calinski-Harabasz Index** | **Davies-Bouldin Index** |
| 2 | 0.71 | 493.04 | 0.42 |
| 3 | 0.66 | 616.12 | 0.46 |
| 4 | 0.64 | 805.82 | 0.45 |
| 5 | 0.64 | 846.23 | 0.48 |
| 6 | 0.57 | 988.49 | 0.51 |
| 7 | 0.58 | 1,229.16 | 0.49 |
| 8 | 0.57 | 1,384.24 | 0.48 |
| 9 | 0.56 | 1,485.76 | 0.49 |
| 10 | 0.56 | 1,465.68 | 0.47 |

**Table S4.** ICD-code prefixes according to cluster identity.

| Cluster | ICD-10 Code Prefix | ICD-10 Code Description | Sample Size (N,%) |  | Admission Rate (%) | SD |
| --- | --- | --- | --- | --- | --- | --- |
| 1 | J06 | Acute upper respiratory infections of multiple and unspecified sites | 16160 (12.3%) |  | 7.1% (6.7%, 7.5%) | 0.26 |
| 1 | M54 | Dorsalgia | 10087 (7.7%) |  | 3.2% (2.8%, 3.5%) | 0.17 |
| 1 | R07 | Pain in throat and chest | 8362 (6.4%) |  | 1.9% (1.6%, 2.2%) | 0.14 |
| 1 | T14 | Injury of unspecified body region | 7406 (5.6%) |  | 5.9% (5.4%, 6.4%) | 0.24 |
| 1 | M25 | Other joint disorder, not elsewhere classified | 4653 (3.5%) |  | 7.4% (6.7%, 8.2%) | 0.26 |
| 1 | M79 | Other and unspecified soft tissue disorders, not elsewhere classified | 3803 (2.9%) |  | 7.8% (7.0%, 8.7%) | 0.27 |
| 1 | S09 | Other and unspecified injuries of head | 3758 (2.9%) |  | 1.8% (1.4%, 2.2%) | 0.13 |
| 1 | R42 | Dizziness and giddiness | 3502 (2.7%) |  | 9.3% (8.3%, 10.2%) | 0.29 |
| 1 | K29 | Gastritis and duodenitis | 2350 (1.8%) |  | 8.1% (7.0%, 9.2%) | 0.27 |
| 1 | R29 | Other symptoms and signs involving the nervous and musculoskeletal systems | 2238 (1.7%) |  | 7.0% (6.0%, 8.1%) | 0.26 |
| 1 | N23 | Unspecified renal colic | 2083 (1.6%) |  | 2.5% (1.8%, 3.2%) | 0.16 |
| 1 | J03 | Acute tonsillitis | 1600 (1.2%) |  | 5.6% (4.4%, 6.7%) | 0.23 |
| 1 | I10 | Essential (primary) hypertension | 1597 (1.2%) |  | 6.5% (5.3%, 7.7%) | 0.25 |
| 1 | S93 | Dislocation and sprain of joints and ligaments at ankle, foot and toe level | 1596 (1.2%) |  | 0.2% (0.0%, 0.4%) | 0.04 |
| 1 | K59 | Other functional intestinal disorders | 1581 (1.2%) |  | 8.3% (6.9%, 9.6%) | 0.28 |
| 1 | S01 | Open wound of head | 1558 (1.2%) |  | 0.6% (0.2%, 1.0%) | 0.08 |
| 1 | S92 | Fracture of foot and toe, except ankle | 1515 (1.2%) |  | 3.4% (2.5%, 4.3%) | 0.18 |
| 1 | R52 | Pain, unspecified | 1456 (1.1%) |  | 2.8% (2.0%, 3.7%) | 0.17 |
| 1 | R05 | Cough | 1333 (1.0%) |  | 11.8% (10.0%, 13.5%) | 0.32 |
| 1 | S62 | Fracture at wrist and hand level | 1265 (1.0%) |  | 4.7% (3.6%, 5.9%) | 0.21 |
| 1 | M47 | Spondylosis | 1231 (0.9%) |  | 3.1% (2.1%, 4.1%) | 0.17 |
| 1 | H60 | Otitis externa | 1208 (0.9%) |  | 1.2% (0.6%, 1.8%) | 0.11 |
| 1 | H10 | Conjunctivitis | 1205 (0.9%) |  | 0.2% (0.0%, 0.5%) | 0.05 |
| 1 | R68 | Other general symptoms and signs | 1161 (0.9%) |  | 3.4% (2.4%, 4.5%) | 0.18 |
| 1 | L50 | Urticaria | 1139 (0.9%) |  | 3.1% (2.1%, 4.1%) | 0.17 |
| 1 | J45 | Asthma | 1073 (0.8%) |  | 8.6% (6.9%, 10.2%) | 0.28 |
| 1 | H57 | Other disorders of eye and adnexa | 1059 (0.8%) |  | 3.8% (2.6%, 4.9%) | 0.19 |
| 1 | S00 | Superficial injury of head | 1059 (0.8%) |  | 0.6% (0.1%, 1.0%) | 0.08 |
| 1 | K08 | Other disorders of teeth and supporting structures | 949 (0.7%) |  | 0.8% (0.3%, 1.4%) | 0.09 |
| 1 | S52 | Fracture of forearm | 946 (0.7%) |  | 4.8% (3.4%, 6.1%) | 0.21 |
| 1 | T17 | Foreign body in respiratory tract | 918 (0.7%) |  | 11.1% (9.1%, 13.1%) | 0.31 |
| 1 | G43 | Migraine | 879 (0.7%) |  | 7.2% (5.5%, 8.9%) | 0.26 |
| 1 | J02 | Acute pharyngitis | 848 (0.6%) |  | 3.9% (2.6%, 5.2%) | 0.19 |
| 1 | M19 | Other and unspecified osteoarthritis | 828 (0.6%) |  | 4.6% (3.2%, 6.0%) | 0.21 |
| 1 | B34 | Viral infection of unspecified site | 813 (0.6%) |  | 8.6% (6.7%, 10.5%) | 0.28 |
| 1 | Y09 | Assault by unspecified means | 771 (0.6%) |  | 0.5% (0.0%, 1.0%) | 0.07 |
| 1 | K21 | Gastro-esophageal reflux disease | 761 (0.6%) |  | 5.0% (3.4%, 6.5%) | 0.22 |
| 1 | S82 | Fracture of lower leg, including ankle | 752 (0.6%) |  | 11.6% (9.3%, 13.9%) | 0.32 |
| 1 | H66 | Suppurative and unspecified otitis media | 747 (0.6%) |  | 1.3% (0.5%, 2.2%) | 0.12 |
| 1 | M17 | Osteoarthritis of knee | 701 (0.5%) |  | 3.9% (2.4%, 5.3%) | 0.19 |
| 1 | S83 | Dislocation and sprain of joints and ligaments of knee | 690 (0.5%) |  | 0.3% (0.0%, 0.7%) | 0.05 |
| 1 | H11 | Other disorders of conjunctiva | 685 (0.5%) |  | 0.1% (0.0%, 0.4%) | 0.04 |
| 1 | S90 | Superficial injury of ankle, foot and toes | 655 (0.5%) |  | 0.9% (0.2%, 1.6%) | 0.10 |
| 1 | R00 | Abnormalities of heart beat | 649 (0.5%) |  | 6.8% (4.8%, 8.7%) | 0.25 |
| 1 | S80 | Superficial injury of knee and lower leg | 649 (0.5%) |  | 1.5% (0.6%, 2.5%) | 0.12 |
| 1 | T78 | Adverse effects, not elsewhere classified | 608 (0.5%) |  | 1.6% (0.6%, 2.7%) | 0.13 |
| 1 | H81 | Disorders of vestibular function | 606 (0.5%) |  | 2.0% (0.9%, 3.1%) | 0.14 |
| 1 | T30 | Burn and corrosion, body region unspecified | 600 (0.5%) |  | 8.3% (6.1%, 10.5%) | 0.28 |
| 1 | K30 | Functional dyspepsia | 597 (0.5%) |  | 5.4% (3.6%, 7.2%) | 0.23 |
| 1 | M51 | Thoracic, thoracolumbar, and lumbosacral intervertebral disc disorders | 590 (0.4%) |  | 3.7% (2.2%, 5.3%) | 0.19 |
| 1 | S60 | Superficial injury of wrist, hand and fingers | 561 (0.4%) |  | 1.8% (0.7%, 2.9%) | 0.13 |
| 1 | V98 | Other specified transport accidents | 528 (0.4%) |  | 0.4% (0.0%, 0.9%) | 0.06 |
| 1 | W19 | Unspecified fall | 468 (0.4%) |  | 0.9% (0.0%, 1.7%) | 0.09 |
| 1 | M75 | Shoulder lesions | 449 (0.3%) |  | 0.4% (0.0%, 1.1%) | 0.07 |
| 1 | L60 | Nail disorders | 444 (0.3%) |  | 0.2% (0.0%, 0.7%) | 0.05 |
| 1 | S13 | Dislocation and sprain of joints and ligaments at neck level | 441 (0.3%) |  | 0.2% (0.0%, 0.7%) | 0.05 |
| 1 | J32 | Chronic sinusitis | 425 (0.3%) |  | 1.6% (0.4%, 2.9%) | 0.13 |
| 1 | S02 | Fracture of skull and facial bones | 423 (0.3%) |  | 5.0% (2.9%, 7.0%) | 0.22 |
| 1 | S42 | Fracture of shoulder and upper arm | 408 (0.3%) |  | 6.6% (4.2%, 9.0%) | 0.25 |
| 1 | H93 | Other disorders of ear, not elsewhere classified | 405 (0.3%) |  | 0.7% (0.0%, 1.6%) | 0.09 |
| 1 | H92 | Otalgia and effusion of ear | 403 (0.3%) |  | 0.7% (0.0%, 1.6%) | 0.09 |
| 1 | S91 | Open wound of ankle, foot and toes | 395 (0.3%) |  | 10.4% (7.4%, 13.4%) | 0.31 |
| 1 | H61 | Other disorders of external ear | 393 (0.3%) |  | 2.8% (1.2%, 4.4%) | 0.17 |
| 1 | G44 | Other headache syndromes | 391 (0.3%) |  | 2.8% (1.2%, 4.5%) | 0.17 |
| 1 | N45 | Orchitis and epididymitis | 380 (0.3%) |  | 3.9% (2.0%, 5.9%) | 0.19 |
| 1 | G47 | Sleep disorders | 349 (0.3%) |  | 3.2% (1.3%, 5.0%) | 0.17 |
| 1 | H00 | Hordeolum and chalazion | 346 (0.3%) |  | 0.3% (0.0%, 0.9%) | 0.05 |
| 1 | B01 | Varicella [chickenpox] | 336 (0.3%) |  | 9.8% (6.6%, 13.0%) | 0.30 |
| 1 | L98 | Other disorders of skin and subcutaneous tissue, not elsewhere classified | 336 (0.3%) |  | 11.3% (7.9%, 14.7%) | 0.32 |
| 1 | T88 | Other complications of surgical and medical care, not elsewhere classified | 331 (0.3%) |  | 9.1% (6.0%, 12.2%) | 0.29 |
| 1 | H44 | Disorders of globe | 328 (0.2%) |  | 7.9% (5.0%, 10.9%) | 0.27 |
| 1 | S05 | Injury of eye and orbit | 298 (0.2%) |  | 7.7% (4.7%, 10.7%) | 0.27 |
| 1 | T09 | Other injuries of spine and trunk, level unspecified | 297 (0.2%) |  | 0.7% (0.0%, 1.6%) | 0.08 |
| 1 | T16 | Foreign body in ear | 297 (0.2%) |  | 0.0% (0.0%, 0.0%) | 0.00 |
| 1 | F41 | Other anxiety disorders | 291 (0.2%) |  | 4.5% (2.1%, 6.8%) | 0.21 |
| 1 | H91 | Other and unspecified hearing loss | 283 (0.2%) |  | 1.8% (0.2%, 3.3%) | 0.13 |
| 1 | M77 | Other enthesopathies | 283 (0.2%) |  | 0.7% (0.0%, 1.7%) | 0.08 |
| 1 | J39 | Other diseases of upper respiratory tract | 277 (0.2%) |  | 4.7% (2.2%, 7.2%) | 0.21 |
| 1 | S63 | Dislocation and sprain of joints and ligaments at wrist and hand level | 273 (0.2%) |  | 0.7% (0.0%, 1.7%) | 0.09 |
| 1 | N50 | Other and unspecified disorders of male genital organs | 272 (0.2%) |  | 5.9% (3.1%, 8.7%) | 0.24 |
| 1 | R33 | Retention of urine | 270 (0.2%) |  | 11.9% (8.0%, 15.7%) | 0.32 |
| 1 | S20 | Superficial injury of thorax | 270 (0.2%) |  | 0.4% (0.0%, 1.1%) | 0.06 |
| 1 | S22 | Fracture of rib(s), sternum and thoracic spine | 265 (0.2%) |  | 4.9% (2.3%, 7.5%) | 0.22 |
| 1 | G51 | Facial nerve disorders | 265 (0.2%) |  | 9.8% (6.2%, 13.4%) | 0.30 |
| 1 | I48 | Atrial fibrillation and flutter | 264 (0.2%) |  | 4.2% (1.8%, 6.6%) | 0.20 |
| 1 | R09 | Other symptoms and signs involving the circulatory and respiratory system | 264 (0.2%) |  | 6.1% (3.2%, 8.9%) | 0.24 |
| 1 | T13 | Other injuries of lower limb, level unspecified | 260 (0.2%) |  | 3.1% (1.0%, 5.2%) | 0.17 |
| 1 | S40 | Superficial injury of shoulder and upper arm | 259 (0.2%) |  | 0.0% (0.0%, 0.0%) | 0.00 |
| 1 | F10 | Alcohol related disorders | 259 (0.2%) |  | 3.1% (1.0%, 5.2%) | 0.17 |
| 1 | M72 | Fibroblastic disorders | 255 (0.2%) |  | 2.7% (0.7%, 4.8%) | 0.16 |
| 1 | T11 | Other injuries of upper limb, level unspecified | 242 (0.2%) |  | 5.0% (2.2%, 7.7%) | 0.22 |
| 1 | H43 | Disorders of vitreous body | 236 (0.2%) |  | 0.4% (0.0%, 1.3%) | 0.07 |
| 1 | N20 | Calculus of kidney and ureter | 234 (0.2%) |  | 3.0% (0.8%, 5.2%) | 0.17 |
| 1 | T08 | Fracture of spine, level unspecified | 233 (0.2%) |  | 7.7% (4.3%, 11.2%) | 0.27 |
| 1 | N94 | Pain and other conditions associated with female genital organs and menstrual cycle | 232 (0.2%) |  | 1.7% (0.0%, 3.4%) | 0.13 |
| 1 | L29 | Pruritus | 232 (0.2%) |  | 3.9% (1.4%, 6.4%) | 0.19 |
| 1 | S30 | Superficial injury of abdomen, lower back, pelvis and external genitals | 231 (0.2%) |  | 1.3% (0.0%, 2.8%) | 0.11 |
| 1 | S43 | Dislocation and sprain of joints and ligaments of shoulder girdle | 228 (0.2%) |  | 1.3% (0.0%, 2.8%) | 0.11 |
| 1 | N92 | Excessive, frequent and irregular menstruation | 216 (0.2%) |  | 5.1% (2.2%, 8.0%) | 0.22 |
| 1 | S99 | Other and unspecified injuries of ankle and foot | 215 (0.2%) |  | 3.7% (1.2%, 6.3%) | 0.19 |
| 1 | H33 | Retinal detachments and breaks | 214 (0.2%) |  | 7.0% (3.6%, 10.4%) | 0.26 |
| 1 | A08 | Viral and other specified intestinal infections | 212 (0.2%) |  | 5.7% (2.5%, 8.8%) | 0.23 |
| 1 | R56 | Convulsions, not elsewhere classified | 212 (0.2%) |  | 6.6% (3.3%, 9.9%) | 0.25 |
| 1 | N30 | Cystitis | 209 (0.2%) |  | 6.7% (3.3%, 10.1%) | 0.25 |
| 1 | S50 | Superficial injury of elbow and forearm | 196 (0.1%) |  | 0.0% (0.0%, 0.0%) | 0.00 |
| 1 | B09 | Unspecified viral infection characterized by skin and mucous membrane lesions | 194 (0.1%) |  | 6.2% (2.8%, 9.6%) | 0.24 |
| 1 | N64 | Other disorders of breast | 181 (0.1%) |  | 6.1% (2.6%, 9.6%) | 0.24 |
| 1 | B35 | Dermatophytosis | 181 (0.1%) |  | 1.1% (0.0%, 2.6%) | 0.10 |
| 1 | S89 | Other and unspecified injuries of lower leg | 169 (0.1%) |  | 4.1% (1.1%, 7.1%) | 0.20 |
| 1 | L25 | Unspecified contact dermatitis | 169 (0.1%) |  | 4.1% (1.1%, 7.1%) | 0.20 |
| 1 | S70 | Superficial injury of hip and thigh | 167 (0.1%) |  | 3.0% (0.4%, 5.6%) | 0.17 |
| 1 | K04 | Diseases of pulp and periapical tissues | 165 (0.1%) |  | 10.9% (6.2%, 15.7%) | 0.31 |
| 1 | N40 | Benign prostatic hyperplasia | 165 (0.1%) |  | 0.6% (0.0%, 1.8%) | 0.08 |
| 1 | J34 | Other and unspecified disorders of nose and nasal sinuses | 164 (0.1%) |  | 3.0% (0.4%, 5.7%) | 0.17 |
| 1 | S32 | Fracture of lumbar spine and pelvis | 162 (0.1%) |  | 8.6% (4.3%, 13.0%) | 0.28 |
| 1 | H04 | Disorders of lacrimal system | 161 (0.1%) |  | 1.2% (0.0%, 3.0%) | 0.11 |
| 1 | L84 | Corns and callosities | 157 (0.1%) |  | 2.5% (0.1%, 5.0%) | 0.16 |
| 1 | L73 | Other follicular disorders | 155 (0.1%) |  | 9.0% (4.5%, 13.5%) | 0.29 |
| 1 | B37 | Candidiasis | 154 (0.1%) |  | 3.9% (0.8%, 7.0%) | 0.19 |
| 1 | S16 | Injury of muscle, fascia and tendon at neck level | 152 (0.1%) |  | 0.0% (0.0%, 0.0%) | 0.00 |
| 1 | M23 | Internal derangement of knee | 148 (0.1%) |  | 0.7% (0.0%, 2.0%) | 0.08 |
| 1 | L20 | Atopic dermatitis | 148 (0.1%) |  | 10.1% (5.3%, 15.0%) | 0.30 |
| 1 | J31 | Chronic rhinitis, nasopharyngitis and pharyngitis | 146 (0.1%) |  | 0.7% (0.0%, 2.0%) | 0.08 |
| 1 | N48 | Other disorders of penis | 145 (0.1%) |  | 7.6% (3.3%, 11.9%) | 0.27 |
| 1 | H72 | Perforation of tympanic membrane | 144 (0.1%) |  | 0.0% (0.0%, 0.0%) | 0.00 |
| 1 | N93 | Other abnormal uterine and vaginal bleeding | 143 (0.1%) |  | 8.4% (3.8%, 12.9%) | 0.28 |
| 1 | J30 | Vasomotor and allergic rhinitis | 137 (0.1%) |  | 0.0% (0.0%, 0.0%) | 0.00 |
| 1 | T63 | Toxic effect of contact with venomous animals and plants | 136 (0.1%) |  | 2.9% (0.1%, 5.8%) | 0.17 |
| 1 | G56 | Mononeuropathies of upper limb | 136 (0.1%) |  | 0.0% (0.0%, 0.0%) | 0.00 |
| 1 | B08 | Other viral infections characterized by skin and mucous membrane lesions, not elsewhere classified | 135 (0.1%) |  | 9.6% (4.7%, 14.6%) | 0.30 |
| 1 | K05 | Gingivitis and periodontal diseases | 129 (0.1%) |  | 0.0% (0.0%, 0.0%) | 0.00 |
| 1 | T23 | Burn and corrosion of wrist and hand | 128 (0.1%) |  | 4.7% (1.0%, 8.3%) | 0.21 |
| 1 | M20 | Acquired deformities of fingers and toes | 126 (0.1%) |  | 0.8% (0.0%, 2.3%) | 0.09 |
| 1 | K02 | Dental caries | 125 (0.1%) |  | 1.6% (0.0%, 3.8%) | 0.13 |
| 1 | R30 | Pain associated with micturition | 125 (0.1%) |  | 3.2% (0.1%, 6.3%) | 0.18 |
| 1 | K06 | Other disorders of gingiva and edentulous alveolar ridge | 124 (0.1%) |  | 1.6% (0.0%, 3.8%) | 0.13 |
| 1 | H26 | Other cataract | 124 (0.1%) |  | 1.6% (0.0%, 3.8%) | 0.13 |
| 1 | I87 | Other disorders of veins | 123 (0.1%) |  | 10.6% (5.1%, 16.0%) | 0.31 |
| 1 | M71 | Other bursopathies | 117 (0.1%) |  | 7.7% (2.9%, 12.5%) | 0.27 |
| 1 | M43 | Other deforming dorsopathies | 114 (0.1%) |  | 1.8% (0.0%, 4.2%) | 0.13 |
| 1 | M76 | Enthesopathies, lower limb, excluding foot | 114 (0.1%) |  | 0.9% (0.0%, 2.6%) | 0.09 |
| 1 | M48 | Other spondylopathies | 113 (0.1%) |  | 8.0% (3.0%, 13.0%) | 0.27 |
| 1 | D17 | Benign lipomatous neoplasm | 113 (0.1%) |  | 4.4% (0.6%, 8.2%) | 0.21 |
| 1 | J93 | Pneumothorax and air leak | 113 (0.1%) |  | 3.5% (0.1%, 6.9%) | 0.19 |
| 1 | B86 | Scabies | 106 (0.1%) |  | 5.7% (1.3%, 10.1%) | 0.23 |
| 1 | K10 | Other diseases of jaws | 105 (0.1%) |  | 4.8% (0.7%, 8.8%) | 0.21 |
| 1 | G54 | Nerve root and plexus disorders | 103 (0.1%) |  | 6.8% (1.9%, 11.7%) | 0.25 |
| 1 | N47 | Disorders of prepuce | 102 (0.1%) |  | 2.9% (0.0%, 6.2%) | 0.17 |
| 1 | B30 | Viral conjunctivitis | 102 (0.1%) |  | 1.0% (0.0%, 2.9%) | 0.10 |
| 1 | H90 | Conductive and sensorineural hearing loss | 101 (0.1%) |  | 2.0% (0.0%, 4.7%) | 0.14 |
| 1 | A88 | Other viral infections of central nervous system, not elsewhere classified | 99 (0.1%) |  | 0.0% (0.0%, 0.0%) | 0.00 |
| 1 | F43 | Reaction to severe stress, and adjustment disorders | 99 (0.1%) |  | 11.1% (4.9%, 17.3%) | 0.32 |
| 1 | B49 | Unspecified mycosis | 91 (0.1%) |  | 4.4% (0.2%, 8.6%) | 0.21 |
| 1 | R39 | Other and unspecified symptoms and signs involving the genitourinary system | 89 (0.1%) |  | 1.1% (0.0%, 3.3%) | 0.11 |
| 1 | S33 | Dislocation and sprain of joints and ligaments of lumbar spine and pelvis | 89 (0.1%) |  | 0.0% (0.0%, 0.0%) | 0.00 |
| 1 | F45 | Somatoform disorders | 88 (0.1%) |  | 3.4% (0.0%, 7.2%) | 0.18 |
| 1 | T00 | Superficial injuries involving multiple body regions | 87 (0.1%) |  | 4.6% (0.2%, 9.0%) | 0.21 |
| 1 | T59 | Toxic effect of other gases, fumes and vapors | 86 (0.1%) |  | 0.0% (0.0%, 0.0%) | 0.00 |
| 1 | Z03 | Encounter for medical observation for suspected diseases and conditions ruled out | 85 (0.1%) |  | 1.2% (0.0%, 3.5%) | 0.11 |
| 1 | S46 | Injury of muscle, fascia and tendon at shoulder and upper arm level | 83 (0.1%) |  | 0.0% (0.0%, 0.0%) | 0.00 |
| 1 | M94 | Other disorders of cartilage | 79 (0.1%) |  | 1.3% (0.0%, 3.7%) | 0.11 |
| 1 | K07 | Dentofacial anomalies [including malocclusion] and mandibular deformities | 72 (0.1%) |  | 0.0% (0.0%, 0.0%) | 0.00 |
| 1 | J11 | Influenza due to unidentified influenza virus | 72 (0.1%) |  | 8.3% (1.9%, 14.7%) | 0.28 |
| 1 | T22 | Burn and corrosion of shoulder and upper limb, except wrist and hand | 71 (0.1%) |  | 2.8% (0.0%, 6.7%) | 0.17 |
| 1 | E04 | Other nontoxic goiter | 70 (0.1%) |  | 7.1% (1.1%, 13.2%) | 0.26 |
| 1 | H20 | Iridocyclitis | 70 (0.1%) |  | 2.9% (0.0%, 6.8%) | 0.17 |
| 1 | B00 | Herpesviral [herpes simplex] infections | 70 (0.1%) |  | 10.0% (3.0%, 17.0%) | 0.30 |
| 1 | G40 | Epilepsy and recurrent seizures | 70 (0.1%) |  | 10.0% (3.0%, 17.0%) | 0.30 |
| 1 | M67 | Other disorders of synovium and tendon | 69 (0.1%) |  | 0.0% (0.0%, 0.0%) | 0.00 |
| 1 | H01 | Other inflammation of eyelid | 68 (0.1%) |  | 1.5% (0.0%, 4.3%) | 0.12 |
| 1 | R35 | Polyuria | 68 (0.1%) |  | 2.9% (0.0%, 7.0%) | 0.17 |
| 1 | H18 | Other disorders of cornea | 67 (0.1%) |  | 6.0% (0.3%, 11.6%) | 0.24 |
| 1 | S06 | Intracranial injury | 66 (0.1%) |  | 10.6% (3.2%, 18.0%) | 0.31 |
| 1 | N89 | Other noninflammatory disorders of vagina | 65 (0.0%) |  | 3.1% (0.0%, 7.3%) | 0.17 |
| 1 | K14 | Diseases of tongue | 63 (0.0%) |  | 7.9% (1.3%, 14.6%) | 0.27 |
| 1 | H17 | Corneal scars and opacities | 63 (0.0%) |  | 0.0% (0.0%, 0.0%) | 0.00 |
| 1 | I47 | Paroxysmal tachycardia | 62 (0.0%) |  | 0.0% (0.0%, 0.0%) | 0.00 |
| 1 | M70 | Soft tissue disorders related to use, overuse and pressure | 62 (0.0%) |  | 4.8% (0.0%, 10.2%) | 0.22 |
| 1 | Z02 | Encounter for administrative examination | 61 (0.0%) |  | 0.0% (0.0%, 0.0%) | 0.00 |
| 1 | Z33 | Pregnant state | 60 (0.0%) |  | 5.0% (0.0%, 10.5%) | 0.22 |
| 1 | B07 | Viral warts | 59 (0.0%) |  | 0.0% (0.0%, 0.0%) | 0.00 |
| 1 | N81 | Female genital prolapse | 58 (0.0%) |  | 0.0% (0.0%, 0.0%) | 0.00 |
| 1 | S53 | Dislocation and sprain of joints and ligaments of elbow | 57 (0.0%) |  | 0.0% (0.0%, 0.0%) | 0.00 |
| 1 | H15 | Disorders of sclera | 56 (0.0%) |  | 7.1% (0.4%, 13.9%) | 0.26 |
| 1 | N95 | Menopausal and other perimenopausal disorders | 55 (0.0%) |  | 7.3% (0.4%, 14.1%) | 0.26 |
| 1 | M16 | Osteoarthritis of hip | 55 (0.0%) |  | 1.8% (0.0%, 5.3%) | 0.13 |
| 1 | N34 | Urethritis and urethral syndrome | 54 (0.0%) |  | 0.0% (0.0%, 0.0%) | 0.00 |
| 1 | O20 | Hemorrhage in early pregnancy | 53 (0.0%) |  | 3.8% (0.0%, 8.9%) | 0.19 |
| 1 | S10 | Superficial injury of neck | 51 (0.0%) |  | 2.0% (0.0%, 5.8%) | 0.14 |
| 1 | S59 | Other and unspecified injuries of elbow and forearm | 51 (0.0%) |  | 2.0% (0.0%, 5.8%) | 0.14 |
| 1 | F07 | Personality and behavioral disorders due to known physiological condition | 51 (0.0%) |  | 5.9% (0.0%, 12.3%) | 0.24 |
| 1 | Z71 | Persons encountering health services for other counseling and medical advice, not elsewhere classified | 51 (0.0%) |  | 3.9% (0.0%, 9.2%) | 0.20 |
| 1 | P12 | Birth injury to scalp | 51 (0.0%) |  | 3.9% (0.0%, 9.2%) | 0.20 |
| 1 | L91 | Hypertrophic disorders of skin | 51 (0.0%) |  | 3.9% (0.0%, 9.2%) | 0.20 |
| 1 | B18 | Chronic viral hepatitis | 50 (0.0%) |  | 0.0% (0.0%, 0.0%) | 0.00 |
| 1 | S73 | Dislocation and sprain of joint and ligaments of hip | 50 (0.0%) |  | 2.0% (0.0%, 5.9%) | 0.14 |
| 1 | J35 | Chronic diseases of tonsils and adenoids | 48 (0.0%) |  | 6.2% (0.0%, 13.1%) | 0.24 |
| 1 | T62 | Toxic effect of other noxious substances eaten as food | 46 (0.0%) |  | 4.3% (0.0%, 10.2%) | 0.21 |
| 1 | M66 | Spontaneous rupture of synovium and tendon | 45 (0.0%) |  | 8.9% (0.6%, 17.2%) | 0.29 |
| 1 | K13 | Other diseases of lip and oral mucosa | 44 (0.0%) |  | 4.5% (0.0%, 10.7%) | 0.21 |
| 1 | J01 | Acute sinusitis | 42 (0.0%) |  | 4.8% (0.0%, 11.2%) | 0.22 |
| 1 | S76 | Injury of muscle, fascia and tendon at hip and thigh level | 41 (0.0%) |  | 2.4% (0.0%, 7.2%) | 0.16 |
| 1 | M53 | Other and unspecified dorsopathies, not elsewhere classified | 41 (0.0%) |  | 7.3% (0.0%, 15.3%) | 0.26 |
| 1 | T25 | Burn and corrosion of ankle and foot | 39 (0.0%) |  | 10.3% (0.7%, 19.8%) | 0.31 |
| 1 | R61 | Generalized hyperhidrosis | 39 (0.0%) |  | 0.0% (0.0%, 0.0%) | 0.00 |
| 1 | T15 | Foreign body on external eye | 38 (0.0%) |  | 0.0% (0.0%, 0.0%) | 0.00 |
| 1 | A64 | Unspecified sexually transmitted disease | 38 (0.0%) |  | 2.6% (0.0%, 7.7%) | 0.16 |
| 1 | F20 | Schizophrenia | 38 (0.0%) |  | 10.5% (0.8%, 20.3%) | 0.31 |
| 1 | Z24 | Need for immunization against certain single bacterial diseases | 38 (0.0%) |  | 0.0% (0.0%, 0.0%) | 0.00 |
| 1 | L70 | Acne | 38 (0.0%) |  | 0.0% (0.0%, 0.0%) | 0.00 |
| 1 | I49 | Other cardiac arrhythmias | 37 (0.0%) |  | 2.7% (0.0%, 7.9%) | 0.16 |
| 1 | D25 | Leiomyoma of uterus | 37 (0.0%) |  | 10.8% (0.8%, 20.8%) | 0.31 |
| 1 | H69 | Other and unspecified disorders of Eustachian tube | 36 (0.0%) |  | 0.0% (0.0%, 0.0%) | 0.00 |
| 1 | R46 | Symptoms and signs involving appearance and behavior | 35 (0.0%) |  | 2.9% (0.0%, 8.4%) | 0.17 |
| 1 | B36 | Other superficial mycoses | 35 (0.0%) |  | 5.7% (0.0%, 13.4%) | 0.24 |
| 1 | S03 | Dislocation and sprain of joints and ligaments of head | 35 (0.0%) |  | 0.0% (0.0%, 0.0%) | 0.00 |
| 1 | J04 | Acute laryngitis and tracheitis | 35 (0.0%) |  | 11.4% (0.9%, 22.0%) | 0.32 |
| 1 | H65 | Nonsuppurative otitis media | 34 (0.0%) |  | 0.0% (0.0%, 0.0%) | 0.00 |
| 1 | Z92 | Personal history of medical treatment | 33 (0.0%) |  | 6.1% (0.0%, 14.2%) | 0.24 |
| 1 | R36 | Urethral discharge | 32 (0.0%) |  | 0.0% (0.0%, 0.0%) | 0.00 |
| 1 | R49 | Voice and resonance disorders | 32 (0.0%) |  | 6.2% (0.0%, 14.6%) | 0.25 |
| 1 | Z84 | Family history of other conditions | 32 (0.0%) |  | 3.1% (0.0%, 9.2%) | 0.18 |
| 1 | T21 | Burn and corrosion of trunk | 31 (0.0%) |  | 6.5% (0.0%, 15.1%) | 0.25 |
| 1 | K01 | Embedded and impacted teeth | 31 (0.0%) |  | 0.0% (0.0%, 0.0%) | 0.00 |
| 1 | S66 | Injury of muscle, fascia and tendon at wrist and hand level | 31 (0.0%) |  | 0.0% (0.0%, 0.0%) | 0.00 |
| 1 | F52 | Sexual dysfunction not due to a substance or known physiological condition | 31 (0.0%) |  | 0.0% (0.0%, 0.0%) | 0.00 |
| 1 | T74 | Adult and child abuse, neglect and other maltreatment, confirmed | 30 (0.0%) |  | 6.7% (0.0%, 15.6%) | 0.25 |
| 1 | S49 | Other and unspecified injuries of shoulder and upper arm | 30 (0.0%) |  | 0.0% (0.0%, 0.0%) | 0.00 |
| 1 | L55 | Sunburn | 30 (0.0%) |  | 0.0% (0.0%, 0.0%) | 0.00 |
| 1 | L74 | Eccrine sweat disorders | 29 (0.0%) |  | 0.0% (0.0%, 0.0%) | 0.00 |
| 1 | N91 | Absent, scanty and rare menstruation | 27 (0.0%) |  | 3.7% (0.0%, 10.8%) | 0.19 |
| 1 | I86 | Varicose veins of other sites | 26 (0.0%) |  | 0.0% (0.0%, 0.0%) | 0.00 |
| 1 | T70 | Effects of air pressure and water pressure | 26 (0.0%) |  | 0.0% (0.0%, 0.0%) | 0.00 |
| 1 | Z20 | Contact with and (suspected) exposure to communicable diseases | 24 (0.0%) |  | 4.2% (0.0%, 12.2%) | 0.20 |
| 1 | N90 | Other noninflammatory disorders of vulva and perineum | 22 (0.0%) |  | 0.0% (0.0%, 0.0%) | 0.00 |
| 1 | M24 | Other specific joint derangements | 22 (0.0%) |  | 4.5% (0.0%, 13.2%) | 0.21 |
| 1 | Z76 | Persons encountering health services in other circumstances | 22 (0.0%) |  | 0.0% (0.0%, 0.0%) | 0.00 |
| 1 | A63 | Other predominantly sexually transmitted diseases, not elsewhere classified | 21 (0.0%) |  | 4.8% (0.0%, 13.9%) | 0.22 |
| 1 | S39 | Other and unspecified injuries of abdomen, lower back, pelvis and external genitals | 21 (0.0%) |  | 0.0% (0.0%, 0.0%) | 0.00 |
| 1 | Q18 | Other congenital malformations of face and neck | 21 (0.0%) |  | 4.8% (0.0%, 13.9%) | 0.22 |
| 1 | Z21 | Asymptomatic human immunodeficiency virus [HIV] infection status | 21 (0.0%) |  | 9.5% (0.0%, 22.1%) | 0.30 |
| 1 | I24 | Other acute ischemic heart diseases | 20 (0.0%) |  | 10.0% (0.0%, 23.1%) | 0.31 |
| 1 | N43 | Hydrocele and spermatocele | 20 (0.0%) |  | 0.0% (0.0%, 0.0%) | 0.00 |
| 1 | T20 | Burn and corrosion of head, face, and neck | 19 (0.0%) |  | 5.3% (0.0%, 15.3%) | 0.23 |
| 1 | N21 | Calculus of lower urinary tract | 19 (0.0%) |  | 0.0% (0.0%, 0.0%) | 0.00 |
| 1 | M41 | Scoliosis | 19 (0.0%) |  | 0.0% (0.0%, 0.0%) | 0.00 |
| 1 | Z86 | Personal history of certain other diseases | 19 (0.0%) |  | 5.3% (0.0%, 15.3%) | 0.23 |
| 1 | L81 | Other disorders of pigmentation | 19 (0.0%) |  | 5.3% (0.0%, 15.3%) | 0.23 |
| 1 | J33 | Nasal polyp | 18 (0.0%) |  | 0.0% (0.0%, 0.0%) | 0.00 |
| 1 | T10 | Fracture of upper limb, level unspecified | 18 (0.0%) |  | 5.6% (0.0%, 16.1%) | 0.24 |
| 1 | L21 | Seborrheic dermatitis | 18 (0.0%) |  | 11.1% (0.0%, 25.6%) | 0.32 |
| 1 | M22 | Disorder of patella | 17 (0.0%) |  | 0.0% (0.0%, 0.0%) | 0.00 |
| 1 | D18 | Hemangioma and lymphangioma, any site | 17 (0.0%) |  | 0.0% (0.0%, 0.0%) | 0.00 |
| 1 | H83 | Other diseases of inner ear | 16 (0.0%) |  | 6.2% (0.0%, 18.1%) | 0.25 |
| 1 | H35 | Other retinal disorders | 16 (0.0%) |  | 0.0% (0.0%, 0.0%) | 0.00 |
| 1 | H52 | Disorders of refraction and accommodation | 16 (0.0%) |  | 0.0% (0.0%, 0.0%) | 0.00 |
| 1 | N32 | Other disorders of bladder | 16 (0.0%) |  | 0.0% (0.0%, 0.0%) | 0.00 |
| 1 | M92 | Other juvenile osteochondrosis | 16 (0.0%) |  | 0.0% (0.0%, 0.0%) | 0.00 |
| 1 | L23 | Allergic contact dermatitis | 16 (0.0%) |  | 6.2% (0.0%, 18.1%) | 0.25 |
| 1 | L24 | Irritant contact dermatitis | 16 (0.0%) |  | 0.0% (0.0%, 0.0%) | 0.00 |
| 1 | B26 | Mumps | 15 (0.0%) |  | 0.0% (0.0%, 0.0%) | 0.00 |
| 1 | S29 | Other and unspecified injuries of thorax | 15 (0.0%) |  | 0.0% (0.0%, 0.0%) | 0.00 |
| 2 | A09 | Infectious gastroenteritis and colitis, unspecified | 8272 (18.7%) |  | 12.2% (11.5%, 12.9%) | 0.33 |
| 2 | R10 | Abdominal and pelvic pain | 4985 (11.2%) |  | 29.8% (28.6%, 31.1%) | 0.46 |
| 2 | N39 | Other disorders of urinary system | 3798 (8.6%) |  | 20.8% (19.5%, 22.1%) | 0.41 |
| 2 | R51 | Headache | 3370 (7.6%) |  | 20.3% (18.9%, 21.7%) | 0.40 |
| 2 | S61 | Open wound of wrist, hand and fingers | 1744 (3.9%) |  | 14.4% (12.8%, 16.1%) | 0.35 |
| 2 | R21 | Rash and other nonspecific skin eruption | 1310 (3.0%) |  | 17.9% (15.9%, 20.0%) | 0.38 |
| 2 | M10 | Gout | 1295 (2.9%) |  | 17.5% (15.5%, 19.6%) | 0.38 |
| 2 | B02 | Zoster [herpes zoster] | 1233 (2.8%) |  | 19.4% (17.2%, 21.6%) | 0.40 |
| 2 | I84 | Haemorrhoids | 1111 (2.5%) |  | 18.2% (15.9%, 20.4%) | 0.39 |
| 2 | H53 | Visual disturbances | 934 (2.1%) |  | 15.7% (13.4%, 18.1%) | 0.36 |
| 2 | L30 | Other and unspecified dermatitis | 904 (2.0%) |  | 13.1% (10.9%, 15.2%) | 0.34 |
| 2 | R31 | Hematuria | 877 (2.0%) |  | 14.1% (11.8%, 16.4%) | 0.35 |
| 2 | R22 | Localized swelling, mass and lump of skin and subcutaneous tissue | 848 (1.9%) |  | 29.1% (26.1%, 32.2%) | 0.45 |
| 2 | J98 | Other respiratory disorders | 760 (1.7%) |  | 30.1% (26.9%, 33.4%) | 0.46 |
| 2 | S69 | Other and unspecified injuries of wrist, hand and finger(s) | 714 (1.6%) |  | 12.5% (10.0%, 14.9%) | 0.33 |
| 2 | R06 | Abnormalities of breathing | 673 (1.5%) |  | 21.0% (17.9%, 24.0%) | 0.41 |
| 2 | R11 | Nausea and vomiting | 656 (1.5%) |  | 32.8% (29.2%, 36.4%) | 0.47 |
| 2 | T81 | Complications of procedures, not elsewhere classified | 528 (1.2%) |  | 33.5% (29.5%, 37.5%) | 0.47 |
| 2 | R55 | Syncope and collapse | 474 (1.1%) |  | 15.2% (12.0%, 18.4%) | 0.36 |
| 2 | J40 | Bronchitis, not specified as acute or chronic | 458 (1.0%) |  | 14.0% (10.8%, 17.1%) | 0.35 |
| 2 | N63 | Unspecified lump in breast | 393 (0.9%) |  | 21.6% (17.6%, 25.7%) | 0.41 |
| 2 | R20 | Disturbances of skin sensation | 392 (0.9%) |  | 18.4% (14.5%, 22.2%) | 0.39 |
| 2 | K80 | Cholelithiasis | 351 (0.8%) |  | 19.7% (15.5%, 23.8%) | 0.40 |
| 2 | M65 | Synovitis and tenosynovitis | 334 (0.8%) |  | 17.4% (13.3%, 21.4%) | 0.38 |
| 2 | I83 | Varicose veins of lower extremities | 309 (0.7%) |  | 23.0% (18.3%, 27.7%) | 0.42 |
| 2 | R59 | Enlarged lymph nodes | 292 (0.7%) |  | 15.8% (11.6%, 19.9%) | 0.36 |
| 2 | R60 | Edema, not elsewhere classified | 280 (0.6%) |  | 29.6% (24.3%, 35.0%) | 0.46 |
| 2 | L72 | Follicular cysts of skin and subcutaneous tissue | 277 (0.6%) |  | 14.1% (10.0%, 18.2%) | 0.35 |
| 2 | H16 | Keratitis | 274 (0.6%) |  | 27.4% (22.1%, 32.7%) | 0.45 |
| 2 | I20 | Angina pectoris | 265 (0.6%) |  | 18.9% (14.2%, 23.6%) | 0.39 |
| 2 | K40 | Inguinal hernia | 261 (0.6%) |  | 21.8% (16.8%, 26.9%) | 0.41 |
| 2 | K12 | Stomatitis and related lesions | 247 (0.6%) |  | 15.4% (10.9%, 19.9%) | 0.36 |
| 2 | T18 | Foreign body in alimentary tract | 238 (0.5%) |  | 24.8% (19.3%, 30.3%) | 0.43 |
| 2 | K11 | Diseases of salivary glands | 212 (0.5%) |  | 13.7% (9.1%, 18.3%) | 0.34 |
| 2 | W54 | Contact with dog | 212 (0.5%) |  | 21.7% (16.1%, 27.2%) | 0.41 |
| 2 | F32 | Major depressive disorder, single episode | 212 (0.5%) |  | 17.9% (12.8%, 23.1%) | 0.38 |
| 2 | R25 | Abnormal involuntary movements | 204 (0.5%) |  | 12.7% (8.2%, 17.3%) | 0.33 |
| 2 | M13 | Other arthritis | 193 (0.4%) |  | 30.6% (24.1%, 37.1%) | 0.46 |
| 2 | R73 | Elevated blood glucose level | 190 (0.4%) |  | 29.5% (23.0%, 36.0%) | 0.46 |
| 2 | R58 | Hemorrhage, not elsewhere classified | 158 (0.4%) |  | 15.2% (9.6%, 20.8%) | 0.36 |
| 2 | H54 | Blindness and low vision | 157 (0.4%) |  | 12.7% (7.5%, 18.0%) | 0.33 |
| 2 | K60 | Fissure and fistula of anal and rectal regions | 146 (0.3%) |  | 26.0% (18.9%, 33.1%) | 0.44 |
| 2 | G50 | Disorders of trigeminal nerve | 143 (0.3%) |  | 19.6% (13.1%, 26.1%) | 0.40 |
| 2 | T24 | Burn and corrosion of lower limb, except ankle and foot | 142 (0.3%) |  | 12.7% (7.2%, 18.1%) | 0.33 |
| 2 | E05 | Thyrotoxicosis [hyperthyroidism] | 137 (0.3%) |  | 28.5% (20.9%, 36.0%) | 0.45 |
| 2 | R14 | Flatulence and related conditions | 131 (0.3%) |  | 15.3% (9.1%, 21.4%) | 0.36 |
| 2 | N75 | Diseases of Bartholin's gland | 131 (0.3%) |  | 19.1% (12.4%, 25.8%) | 0.39 |
| 2 | J20 | Acute bronchitis | 126 (0.3%) |  | 14.3% (8.2%, 20.4%) | 0.35 |
| 2 | E86 | Volume depletion | 124 (0.3%) |  | 26.6% (18.8%, 34.4%) | 0.44 |
| 2 | H40 | Glaucoma | 123 (0.3%) |  | 22.0% (14.6%, 29.3%) | 0.42 |
| 2 | O21 | Excessive vomiting in pregnancy | 121 (0.3%) |  | 27.3% (19.3%, 35.2%) | 0.45 |
| 2 | J36 | Peritonsillar abscess | 120 (0.3%) |  | 16.7% (10.0%, 23.3%) | 0.37 |
| 2 | J44 | Other chronic obstructive pulmonary disease | 113 (0.3%) |  | 31.9% (23.3%, 40.4%) | 0.47 |
| 2 | I88 | Nonspecific lymphadenitis | 112 (0.3%) |  | 13.4% (7.1%, 19.7%) | 0.34 |
| 2 | N76 | Other inflammation of vagina and vulva | 111 (0.3%) |  | 18.9% (11.6%, 26.2%) | 0.39 |
| 2 | L40 | Psoriasis | 110 (0.2%) |  | 28.2% (19.8%, 36.6%) | 0.45 |
| 2 | K46 | Unspecified abdominal hernia | 108 (0.2%) |  | 16.7% (9.6%, 23.7%) | 0.37 |
| 2 | S51 | Open wound of elbow and forearm | 107 (0.2%) |  | 15.9% (9.0%, 22.8%) | 0.37 |
| 2 | N73 | Other female pelvic inflammatory diseases | 104 (0.2%) |  | 26.0% (17.5%, 34.4%) | 0.44 |
| 2 | E16 | Other disorders of pancreatic internal secretion | 98 (0.2%) |  | 12.2% (5.8%, 18.7%) | 0.33 |
| 2 | H02 | Other disorders of eyelid | 89 (0.2%) |  | 22.5% (13.8%, 31.1%) | 0.42 |
| 2 | G95 | Other and unspecified diseases of spinal cord | 88 (0.2%) |  | 21.6% (13.0%, 30.2%) | 0.41 |
| 2 | R45 | Symptoms and signs involving emotional state | 79 (0.2%) |  | 25.3% (15.7%, 34.9%) | 0.44 |
| 2 | R23 | Other skin changes | 79 (0.2%) |  | 13.9% (6.3%, 21.6%) | 0.35 |
| 2 | T29 | Burns and corrosions of multiple body regions | 78 (0.2%) |  | 16.7% (8.4%, 24.9%) | 0.38 |
| 2 | R32 | Unspecified urinary incontinence | 68 (0.2%) |  | 16.2% (7.4%, 24.9%) | 0.37 |
| 2 | K58 | Irritable bowel syndrome | 67 (0.2%) |  | 17.9% (8.7%, 27.1%) | 0.39 |
| 2 | G62 | Other and unspecified polyneuropathies | 66 (0.1%) |  | 22.7% (12.6%, 32.8%) | 0.42 |
| 2 | K22 | Other diseases of esophagus | 63 (0.1%) |  | 23.8% (13.3%, 34.3%) | 0.43 |
| 2 | I25 | Chronic ischemic heart disease | 57 (0.1%) |  | 12.3% (3.8%, 20.8%) | 0.33 |
| 2 | S67 | Crushing injury of wrist, hand and fingers | 50 (0.1%) |  | 32.0% (19.1%, 44.9%) | 0.47 |
| 2 | M46 | Other inflammatory spondylopathies | 45 (0.1%) |  | 13.3% (3.4%, 23.3%) | 0.34 |
| 2 | A60 | Anogenital herpesviral [herpes simplex] infections | 44 (0.1%) |  | 13.6% (3.5%, 23.8%) | 0.35 |
| 2 | L01 | Impetigo | 41 (0.1%) |  | 17.1% (5.6%, 28.6%) | 0.38 |
| 2 | N13 | Obstructive and reflux uropathy | 39 (0.1%) |  | 33.3% (18.5%, 48.1%) | 0.48 |
| 2 | M21 | Other acquired deformities of limbs | 39 (0.1%) |  | 12.8% (2.3%, 23.3%) | 0.34 |
| 2 | E03 | Other hypothyroidism | 37 (0.1%) |  | 16.2% (4.3%, 28.1%) | 0.37 |
| 2 | S81 | Open wound of knee and lower leg | 33 (0.1%) |  | 24.2% (9.6%, 38.9%) | 0.44 |
| 2 | S86 | Injury of muscle, fascia and tendon at lower leg level | 32 (0.1%) |  | 12.5% (1.0%, 24.0%) | 0.34 |
| 2 | F19 | Other psychoactive substance related disorders | 32 (0.1%) |  | 15.6% (3.0%, 28.2%) | 0.37 |
| 2 | B27 | Infectious mononucleosis | 31 (0.1%) |  | 22.6% (7.9%, 37.3%) | 0.43 |
| 2 | M84 | Disorder of continuity of bone | 31 (0.1%) |  | 25.8% (10.4%, 41.2%) | 0.44 |
| 2 | N80 | Endometriosis | 30 (0.1%) |  | 13.3% (1.2%, 25.5%) | 0.35 |
| 2 | K66 | Other disorders of peritoneum | 26 (0.1%) |  | 26.9% (9.9%, 44.0%) | 0.45 |
| 2 | M89 | Other disorders of bone | 26 (0.1%) |  | 19.2% (4.1%, 34.4%) | 0.40 |
| 2 | E06 | Thyroiditis | 25 (0.1%) |  | 32.0% (13.7%, 50.3%) | 0.48 |
| 2 | N41 | Inflammatory diseases of prostate | 24 (0.1%) |  | 16.7% (1.8%, 31.6%) | 0.38 |
| 2 | L28 | Lichen simplex chronicus and prurigo | 24 (0.1%) |  | 16.7% (1.8%, 31.6%) | 0.38 |
| 2 | K63 | Other diseases of intestine | 23 (0.1%) |  | 30.4% (11.6%, 49.2%) | 0.47 |
| 2 | R43 | Disturbances of smell and taste | 23 (0.1%) |  | 17.4% (1.9%, 32.9%) | 0.39 |
| 2 | K42 | Umbilical hernia | 21 (0.0%) |  | 28.6% (9.2%, 47.9%) | 0.46 |
| 2 | L04 | Acute lymphadenitis | 21 (0.0%) |  | 19.0% (2.3%, 35.8%) | 0.40 |
| 2 | G99 | Other disorders of nervous system in diseases classified elsewhere | 21 (0.0%) |  | 14.3% (0.0%, 29.3%) | 0.36 |
| 2 | N44 | Noninflammatory disorders of testis | 20 (0.0%) |  | 20.0% (2.5%, 37.5%) | 0.41 |
| 2 | X84 | Intentional self-harm by unspecified means | 20 (0.0%) |  | 20.0% (2.5%, 37.5%) | 0.41 |
| 2 | R79 | Other abnormal findings of blood chemistry | 19 (0.0%) |  | 15.8% (0.0%, 32.2%) | 0.37 |
| 2 | K43 | Ventral hernia | 18 (0.0%) |  | 27.8% (7.1%, 48.5%) | 0.46 |
| 2 | R80 | Proteinuria | 18 (0.0%) |  | 22.2% (3.0%, 41.4%) | 0.43 |
| 2 | M45 | Ankylosing spondylitis | 18 (0.0%) |  | 16.7% (0.0%, 33.9%) | 0.38 |
| 2 | T65 | Toxic effect of other and unspecified substances | 18 (0.0%) |  | 16.7% (0.0%, 33.9%) | 0.38 |
| 2 | T26 | Burn and corrosion confined to eye and adnexa | 16 (0.0%) |  | 18.8% (0.0%, 37.9%) | 0.40 |
| 2 | M11 | Other crystal arthropathies | 16 (0.0%) |  | 31.2% (8.5%, 54.0%) | 0.48 |
| 2 | S31 | Open wound of abdomen, lower back, pelvis and external genitals | 15 (0.0%) |  | 20.0% (0.0%, 40.2%) | 0.41 |
| 2 | O00 | Ectopic pregnancy | 15 (0.0%) |  | 26.7% (4.3%, 49.0%) | 0.46 |
| 3 | R50 | Fever of other and unknown origin | 5638 (20.3%) |  | 39.4% (38.1%, 40.7%) | 0.49 |
| 3 | L03 | Cellulitis and acute lymphangitis | 5111 (18.4%) |  | 55.4% (54.0%, 56.7%) | 0.50 |
| 3 | L02 | Cutaneous abscess, furuncle and carbuncle | 4693 (16.9%) |  | 56.8% (55.3%, 58.2%) | 0.50 |
| 3 | J22 | Unspecified acute lower respiratory infection | 1565 (5.6%) |  | 35.3% (33.0%, 37.7%) | 0.48 |
| 3 | R04 | Hemorrhage from respiratory passages | 1522 (5.5%) |  | 53.0% (50.4%, 55.5%) | 0.50 |
| 3 | A90 | Dengue fever [classical dengue] | 1522 (5.5%) |  | 40.4% (37.9%, 42.9%) | 0.49 |
| 3 | L08 | Other local infections of skin and subcutaneous tissue | 681 (2.4%) |  | 35.7% (32.1%, 39.3%) | 0.48 |
| 3 | E14 | Unspecified diabetes mellitus | 572 (2.1%) |  | 43.4% (39.3%, 47.4%) | 0.50 |
| 3 | R19 | Other symptoms and signs involving the digestive system and abdomen | 572 (2.1%) |  | 34.1% (30.2%, 38.0%) | 0.47 |
| 3 | L97 | Non-pressure chronic ulcer of lower limb, not elsewhere classified | 555 (2.0%) |  | 58.2% (54.1%, 62.3%) | 0.49 |
| 3 | N12 | Tubulo-interstitial nephritis, not specified as acute or chronic | 509 (1.8%) |  | 49.9% (45.6%, 54.2%) | 0.50 |
| 3 | T79 | Certain early complications of trauma, not elsewhere classified | 327 (1.2%) |  | 51.1% (45.7%, 56.5%) | 0.50 |
| 3 | R53 | Malaise and fatigue | 322 (1.2%) |  | 37.6% (32.3%, 42.9%) | 0.49 |
| 3 | N61 | Inflammatory disorders of breast | 314 (1.1%) |  | 52.9% (47.3%, 58.4%) | 0.50 |
| 3 | K62 | Other diseases of anus and rectum | 313 (1.1%) |  | 34.8% (29.5%, 40.1%) | 0.48 |
| 3 | R63 | Symptoms and signs concerning food and fluid intake | 302 (1.1%) |  | 48.0% (42.4%, 53.6%) | 0.50 |
| 3 | D64 | Other anemias | 245 (0.9%) |  | 61.6% (55.5%, 67.7%) | 0.49 |
| 3 | N10 | Acute pyelonephritis | 220 (0.8%) |  | 47.3% (40.7%, 53.9%) | 0.50 |
| 3 | R13 | Aphagia and dysphagia | 189 (0.7%) |  | 53.4% (46.3%, 60.6%) | 0.50 |
| 3 | M06 | Other rheumatoid arthritis | 184 (0.7%) |  | 38.6% (31.6%, 45.6%) | 0.49 |
| 3 | C50 | Malignant neoplasm of breast | 161 (0.6%) |  | 56.5% (48.9%, 64.2%) | 0.50 |
| 3 | D69 | Purpura and other hemorrhagic conditions | 157 (0.6%) |  | 61.8% (54.2%, 69.4%) | 0.49 |
| 3 | T85 | Complications of other internal prosthetic devices, implants and grafts | 151 (0.5%) |  | 45.7% (37.7%, 53.6%) | 0.50 |
| 3 | L05 | Pilonidal cyst and sinus | 145 (0.5%) |  | 55.9% (47.8%, 63.9%) | 0.50 |
| 3 | T83 | Complications of genitourinary prosthetic devices, implants and grafts | 116 (0.4%) |  | 44.8% (35.8%, 53.9%) | 0.50 |
| 3 | R91 | Abnormal findings on diagnostic imaging of lung | 102 (0.4%) |  | 51.0% (41.3%, 60.7%) | 0.50 |
| 3 | B99 | Other and unspecified infectious diseases | 96 (0.3%) |  | 50.0% (40.0%, 60.0%) | 0.50 |
| 3 | N83 | Noninflammatory disorders of ovary, fallopian tube and broad ligament | 92 (0.3%) |  | 41.3% (31.2%, 51.4%) | 0.50 |
| 3 | S72 | Fracture of femur | 74 (0.3%) |  | 47.3% (35.9%, 58.7%) | 0.50 |
| 3 | I21 | Acute myocardial infarction | 68 (0.2%) |  | 42.6% (30.9%, 54.4%) | 0.50 |
| 3 | K52 | Other and unspecified noninfective gastroenteritis and colitis | 66 (0.2%) |  | 42.4% (30.5%, 54.3%) | 0.50 |
| 3 | J15 | Bacterial pneumonia, not elsewhere classified | 66 (0.2%) |  | 45.5% (33.4%, 57.5%) | 0.50 |
| 3 | L27 | Dermatitis due to substances taken internally | 65 (0.2%) |  | 41.5% (29.6%, 53.5%) | 0.50 |
| 3 | G20 | Parkinson's disease | 60 (0.2%) |  | 43.3% (30.8%, 55.9%) | 0.50 |
| 3 | I89 | Other noninfective disorders of lymphatic vessels and lymph nodes | 57 (0.2%) |  | 50.9% (37.9%, 63.9%) | 0.50 |
| 3 | R94 | Abnormal results of function studies | 57 (0.2%) |  | 35.1% (22.7%, 47.5%) | 0.48 |
| 3 | M32 | Systemic lupus erythematosus (SLE) | 53 (0.2%) |  | 62.3% (49.2%, 75.3%) | 0.49 |
| 3 | R26 | Abnormalities of gait and mobility | 51 (0.2%) |  | 60.8% (47.4%, 74.2%) | 0.49 |
| 3 | F29 | Unspecified psychosis not due to a substance or known physiological condition | 49 (0.2%) |  | 44.9% (31.0%, 58.8%) | 0.50 |
| 3 | H05 | Disorders of orbit | 46 (0.2%) |  | 45.7% (31.3%, 60.0%) | 0.50 |
| 3 | C11 | Malignant neoplasm of nasopharynx | 46 (0.2%) |  | 37.0% (23.0%, 50.9%) | 0.49 |
| 3 | H34 | Retinal vascular occlusions | 45 (0.2%) |  | 55.6% (41.0%, 70.1%) | 0.50 |
| 3 | P59 | Neonatal jaundice from other and unspecified causes | 41 (0.1%) |  | 39.0% (24.1%, 54.0%) | 0.49 |
| 3 | I95 | Hypotension | 40 (0.1%) |  | 47.5% (32.0%, 63.0%) | 0.51 |
| 3 | K76 | Other diseases of liver | 39 (0.1%) |  | 41.0% (25.6%, 56.5%) | 0.50 |
| 3 | N49 | Inflammatory disorders of male genital organs, not elsewhere classified | 36 (0.1%) |  | 41.7% (25.6%, 57.8%) | 0.50 |
| 3 | F50 | Eating disorders | 35 (0.1%) |  | 45.7% (29.2%, 62.2%) | 0.51 |
| 3 | R44 | Other symptoms and signs involving general sensations and perceptions | 34 (0.1%) |  | 61.8% (45.4%, 78.1%) | 0.49 |
| 3 | E11 | Type 2 diabetes mellitus | 31 (0.1%) |  | 38.7% (21.6%, 55.9%) | 0.50 |
| 3 | C80 | Malignant neoplasm without specification of site | 31 (0.1%) |  | 51.6% (34.0%, 69.2%) | 0.51 |
| 3 | D48 | Neoplasm of uncertain behavior of other and unspecified sites | 31 (0.1%) |  | 41.9% (24.6%, 59.3%) | 0.50 |
| 3 | T50 | Poisoning by, adverse effect of and underdosing of diuretics and other and unspecified drugs, medicaments and biological substances | 30 (0.1%) |  | 40.0% (22.5%, 57.5%) | 0.50 |
| 3 | R40 | Somnolence, stupor and coma | 30 (0.1%) |  | 40.0% (22.5%, 57.5%) | 0.50 |
| 3 | Z89 | Acquired absence of limb | 29 (0.1%) |  | 55.2% (37.1%, 73.3%) | 0.51 |
| 3 | L53 | Other erythematous conditions | 28 (0.1%) |  | 53.6% (35.1%, 72.0%) | 0.51 |
| 3 | C61 | Malignant neoplasm of prostate | 27 (0.1%) |  | 48.1% (29.3%, 67.0%) | 0.51 |
| 3 | F03 | Unspecified dementia | 24 (0.1%) |  | 41.7% (21.9%, 61.4%) | 0.50 |
| 3 | G25 | Other extrapyramidal and movement disorders | 24 (0.1%) |  | 45.8% (25.9%, 65.8%) | 0.51 |
| 3 | R16 | Hepatomegaly and splenomegaly, not elsewhere classified | 23 (0.1%) |  | 52.2% (31.8%, 72.6%) | 0.51 |
| 3 | N19 | Unspecified kidney failure | 23 (0.1%) |  | 60.9% (40.9%, 80.8%) | 0.50 |
| 3 | F31 | Bipolar disorder | 23 (0.1%) |  | 34.8% (15.3%, 54.2%) | 0.49 |
| 3 | Q61 | Cystic kidney disease | 21 (0.1%) |  | 52.4% (31.0%, 73.7%) | 0.51 |
| 3 | M60 | Myositis | 19 (0.1%) |  | 42.1% (19.9%, 64.3%) | 0.51 |
| 3 | I82 | Other venous embolism and thrombosis | 16 (0.1%) |  | 37.5% (13.8%, 61.2%) | 0.50 |
| 3 | L52 | Erythema nodosum | 15 (0.1%) |  | 53.3% (28.1%, 78.6%) | 0.52 |
| 4 | J18 | Pneumonia, unspecified organism | 2572 (21.9%) |  | 67.9% (66.1%, 69.7%) | 0.47 |
| 4 | A41 | Other sepsis | 1134 (9.6%) |  | 95.4% (94.2%, 96.6%) | 0.21 |
| 4 | K61 | Abscess of anal and rectal regions | 910 (7.7%) |  | 71.6% (68.7%, 74.6%) | 0.45 |
| 4 | E87 | Other disorders of fluid, electrolyte and acid-base balance | 718 (6.1%) |  | 78.7% (75.7%, 81.7%) | 0.41 |
| 4 | N18 | Chronic kidney disease (CKD) | 337 (2.9%) |  | 72.7% (67.9%, 77.5%) | 0.45 |
| 4 | I96 | Gangrene, not elsewhere classified | 321 (2.7%) |  | 85.4% (81.5%, 89.2%) | 0.35 |
| 4 | K81 | Cholecystitis | 295 (2.5%) |  | 88.8% (85.2%, 92.4%) | 0.32 |
| 4 | K56 | Paralytic ileus and intestinal obstruction without hernia | 273 (2.3%) |  | 93.0% (90.0%, 96.1%) | 0.25 |
| 4 | I80 | Phlebitis and thrombophlebitis | 270 (2.3%) |  | 69.3% (63.8%, 74.8%) | 0.46 |
| 4 | K35 | Acute appendicitis | 259 (2.2%) |  | 87.6% (83.6%, 91.7%) | 0.33 |
| 4 | K37 | Unspecified appendicitis | 243 (2.1%) |  | 87.2% (83.0%, 91.4%) | 0.33 |
| 4 | I73 | Other peripheral vascular diseases | 237 (2.0%) |  | 68.8% (62.9%, 74.7%) | 0.46 |
| 4 | R41 | Other symptoms and signs involving cognitive functions and awareness | 198 (1.7%) |  | 78.8% (73.1%, 84.5%) | 0.41 |
| 4 | R17 | Unspecified jaundice | 197 (1.7%) |  | 91.4% (87.4%, 95.3%) | 0.28 |
| 4 | K83 | Other diseases of biliary tract | 193 (1.6%) |  | 93.8% (90.4%, 97.2%) | 0.24 |
| 4 | I67 | Other cerebrovascular diseases | 189 (1.6%) |  | 79.4% (73.6%, 85.1%) | 0.41 |
| 4 | G45 | Transient cerebral ischemic attacks and related syndromes | 185 (1.6%) |  | 78.9% (73.0%, 84.8%) | 0.41 |
| 4 | M86 | Osteomyelitis | 178 (1.5%) |  | 82.0% (76.4%, 87.7%) | 0.39 |
| 4 | K92 | Other diseases of digestive system | 158 (1.3%) |  | 72.8% (65.8%, 79.7%) | 0.45 |
| 4 | I77 | Other disorders of arteries and arterioles | 144 (1.2%) |  | 83.3% (77.2%, 89.4%) | 0.37 |
| 4 | J47 | Bronchiectasis | 140 (1.2%) |  | 67.1% (59.4%, 74.9%) | 0.47 |
| 4 | M62 | Other disorders of muscle | 123 (1.0%) |  | 70.7% (62.7%, 78.8%) | 0.46 |
| 4 | J90 | Pleural effusion, not elsewhere classified | 115 (1.0%) |  | 83.5% (76.7%, 90.3%) | 0.37 |
| 4 | A16 | Respiratory tuberculosis, not confirmed bacteriologically or histologically | 109 (0.9%) |  | 77.1% (69.2%, 85.0%) | 0.42 |
| 4 | T82 | Complications of cardiac and vascular prosthetic devices, implants and grafts | 108 (0.9%) |  | 81.5% (74.2%, 88.8%) | 0.39 |
| 4 | K75 | Other inflammatory liver diseases | 100 (0.8%) |  | 81.0% (73.3%, 88.7%) | 0.39 |
| 4 | C34 | Malignant neoplasm of bronchus and lung | 94 (0.8%) |  | 77.7% (69.2%, 86.1%) | 0.42 |
| 4 | K85 | Acute pancreatitis | 89 (0.8%) |  | 82.0% (74.0%, 90.0%) | 0.39 |
| 4 | H49 | Paralytic strabismus | 87 (0.7%) |  | 86.2% (79.0%, 93.5%) | 0.35 |
| 4 | K57 | Diverticular disease of intestine | 86 (0.7%) |  | 81.4% (73.2%, 89.6%) | 0.39 |
| 4 | A49 | Bacterial infection of unspecified site | 84 (0.7%) |  | 76.2% (67.1%, 85.3%) | 0.43 |
| 4 | I50 | Heart failure | 79 (0.7%) |  | 72.2% (62.3%, 82.0%) | 0.45 |
| 4 | C18 | Malignant neoplasm of colon | 79 (0.7%) |  | 64.6% (54.0%, 75.1%) | 0.48 |
| 4 | G52 | Disorders of other cranial nerves | 71 (0.6%) |  | 81.7% (72.7%, 90.7%) | 0.39 |
| 4 | M00 | Pyogenic arthritis | 65 (0.6%) |  | 90.8% (83.7%, 97.8%) | 0.29 |
| 4 | G83 | Other paralytic syndromes | 61 (0.5%) |  | 72.1% (60.9%, 83.4%) | 0.45 |
| 4 | C79 | Secondary malignant neoplasm of other and unspecified sites | 58 (0.5%) |  | 74.1% (62.9%, 85.4%) | 0.44 |
| 4 | N17 | Acute kidney failure | 54 (0.5%) |  | 85.2% (75.7%, 94.7%) | 0.36 |
| 4 | R18 | Ascites | 53 (0.5%) |  | 81.1% (70.6%, 91.7%) | 0.39 |
| 4 | C85 | Other specified and unspecified types of non-Hodgkin lymphoma | 49 (0.4%) |  | 79.6% (68.3%, 90.9%) | 0.41 |
| 4 | C22 | Malignant neoplasm of liver and intrahepatic bile ducts | 46 (0.4%) |  | 69.6% (56.3%, 82.9%) | 0.47 |
| 4 | D70 | Neutropenia | 45 (0.4%) |  | 91.1% (82.8%, 99.4%) | 0.29 |
| 4 | C16 | Malignant neoplasm of stomach | 45 (0.4%) |  | 68.9% (55.4%, 82.4%) | 0.47 |
| 4 | M35 | Other systemic involvement of connective tissue | 42 (0.4%) |  | 64.3% (49.8%, 78.8%) | 0.48 |
| 4 | A46 | Erysipelas | 42 (0.4%) |  | 66.7% (52.4%, 80.9%) | 0.48 |
| 4 | K65 | Peritonitis | 40 (0.3%) |  | 72.5% (58.7%, 86.3%) | 0.45 |
| 4 | S68 | Traumatic amputation of wrist, hand and fingers | 40 (0.3%) |  | 70.0% (55.8%, 84.2%) | 0.46 |
| 4 | G70 | Myasthenia gravis and other myoneural disorders | 38 (0.3%) |  | 76.3% (62.8%, 89.8%) | 0.43 |
| 4 | L12 | Pemphigoid | 38 (0.3%) |  | 73.7% (59.7%, 87.7%) | 0.45 |
| 4 | L89 | Pressure ulcer | 38 (0.3%) |  | 63.2% (47.8%, 78.5%) | 0.49 |
| 4 | N04 | Nephrotic syndrome | 37 (0.3%) |  | 67.6% (52.5%, 82.7%) | 0.47 |
| 4 | E83 | Disorders of mineral metabolism | 31 (0.3%) |  | 77.4% (62.7%, 92.1%) | 0.43 |
| 4 | C20 | Malignant neoplasm of rectum | 31 (0.3%) |  | 67.7% (51.3%, 84.2%) | 0.48 |
| 4 | C15 | Malignant neoplasm of esophagus | 30 (0.3%) |  | 86.7% (74.5%, 98.8%) | 0.35 |
| 4 | K74 | Fibrosis and cirrhosis of liver | 29 (0.2%) |  | 69.0% (52.1%, 85.8%) | 0.47 |
| 4 | L51 | Erythema multiforme | 28 (0.2%) |  | 85.7% (72.8%, 98.7%) | 0.36 |
| 4 | K50 | Crohn's disease [regional enteritis] | 27 (0.2%) |  | 63.0% (44.7%, 81.2%) | 0.49 |
| 4 | C25 | Malignant neoplasm of pancreas | 26 (0.2%) |  | 88.5% (76.2%, 100.0%) | 0.33 |
| 4 | D61 | Other aplastic anemias and other bone marrow failure syndromes | 24 (0.2%) |  | 91.7% (80.6%, 100.0%) | 0.28 |
| 4 | D72 | Other disorders of white blood cells | 24 (0.2%) |  | 66.7% (47.8%, 85.5%) | 0.48 |
| 4 | H46 | Optic neuritis | 24 (0.2%) |  | 79.2% (62.9%, 95.4%) | 0.41 |
| 4 | G93 | Other disorders of brain | 24 (0.2%) |  | 75.0% (57.7%, 92.3%) | 0.44 |
| 4 | C95 | Leukemia of unspecified cell type | 23 (0.2%) |  | 100.0% (100.0%, 100.0%) | 0.00 |
| 4 | Z94 | Transplanted organ and tissue status | 23 (0.2%) |  | 73.9% (56.0%, 91.9%) | 0.45 |
| 4 | D75 | Other and unspecified diseases of blood and blood-forming organs | 22 (0.2%) |  | 72.7% (54.1%, 91.3%) | 0.46 |
| 4 | H47 | Other disorders of optic [2nd] nerve and visual pathways | 22 (0.2%) |  | 81.8% (65.7%, 97.9%) | 0.39 |
| 4 | S56 | Injury of muscle, fascia and tendon at forearm level | 22 (0.2%) |  | 77.3% (59.8%, 94.8%) | 0.43 |
| 4 | F05 | Delirium due to known physiological condition | 21 (0.2%) |  | 85.7% (70.7%, 100.0%) | 0.36 |
| 4 | T80 | Complications following infusion, transfusion and therapeutic injection | 20 (0.2%) |  | 75.0% (56.0%, 94.0%) | 0.44 |
| 4 | G35 | Multiple sclerosis | 20 (0.2%) |  | 65.0% (44.1%, 85.9%) | 0.49 |
| 4 | T45 | Poisoning by, adverse effect of and underdosing of primarily systemic and hematological agents, not elsewhere classified | 18 (0.2%) |  | 77.8% (58.6%, 97.0%) | 0.43 |
| 4 | C92 | Myeloid leukemia | 18 (0.2%) |  | 94.4% (83.9%, 100.0%) | 0.24 |
| 4 | G03 | Meningitis due to other and unspecified causes | 17 (0.1%) |  | 94.1% (82.9%, 100.0%) | 0.24 |
| 4 | G81 | Hemiplegia and hemiparesis | 17 (0.1%) |  | 64.7% (42.0%, 87.4%) | 0.49 |
| 4 | F22 | Delusional disorders | 16 (0.1%) |  | 68.8% (46.0%, 91.5%) | 0.48 |
| 4 | C78 | Secondary malignant neoplasm of respiratory and digestive organs | 16 (0.1%) |  | 87.5% (71.3%, 100.0%) | 0.34 |
| 4 | D37 | Neoplasm of uncertain behavior of oral cavity and digestive organs | 16 (0.1%) |  | 75.0% (53.8%, 96.2%) | 0.45 |
| 4 | C64 | Malignant neoplasm of kidney, except renal pelvis | 15 (0.1%) |  | 80.0% (59.8%, 100.0%) | 0.41 |

**Table S5.** ICD-code prefixes stratified by ICD-code blocks.

| **ICD Code Grouping** | **ICD-10 Block** | **Cluster** | **Count and Proportion within Block (n(%))** | **ICD-10 Code** | **ICD-10 Description** | **Presentation Count and Proportion of Whole Cohort (n(%))** |
| --- | --- | --- | --- | --- | --- | --- |
| A00-A09 | Intestinal infectious diseases | 1 | 212 (2.5%) | A08 | Viral and other specified intestinal infections | 212 (0.1%) |
|  |  | 2 | 8272 (97.5%) | A09 | cystitirrteritis and colitis, unspecified | 8272 (3.84%) |
| A15-A19 | Tuberculosis | 4 | 109 (100%) | A16 | Respiratory tuberculosis, not confirmed bacteriologically or histologically | 109 (0.05%) |
| A30-A49 | Other bacterial diseases | 4 | 1260 (100%) | A41 | Other sepsis | 1134 (0.53%) |
|  |  |  |  | A46 | Erysipelas | 42 (0.02%) |
|  |  |  |  | A49 | Bacterial infection of unspecified site | 84 (0.04%) |
| A50-A64 | Infections with a predominantly sexual mode of transmission | 1 | 59 (57.28%) | A63 | Other predominantly sexually transmitted diseases, not elsewhere classified | 21 (0.01%) |
|  |  |  |  | A64 | Unspecified sexually transmitted disease | 38 (0.02%) |
|  |  | 2 | 44 (42.72%) | A60 | Anogenital herpesviral [herpes simplex] infections | 44 (0.02%) |
| A80-A89 | Viral infections of the central nervous system | 1 | 99 (100%) | A88 | Other viral infections of central nervous system, not elsewhere classified | 99 (0.05%) |
| A90-A99 | Arthropod-borne viral fevers and viral haemorrhagic fevers | 3 | 1522 (100%) | A90 | Dengue fever [classical dengue] | 1522 (0.71%) |
| B00-B09 | Viral infections characterized by skin and mucous membrane lesions | 1 | 794 (39.17%) | B00 | Herpesviral [herpes simplex] infections | 70 (0.03%) |
|  |  |  |  | B01 | Varicella [chickenpox] | 336 (0.16%) |
|  |  |  |  | B07 | Viral warts | 59 (0.03%) |
|  |  |  |  | B08 | Other viral infections characterized by skin and mucous membrane lesions, not elsewhere classified | 135 (0.06%) |
|  |  |  |  | B09 | Unspecified viral infection characterized by skin and mucous membrane lesions | 194 (0.09%) |
|  |  | 2 | 1233 (60.83%) | B02 | Zoster [herpes zoster] | 1233 (0.57%) |
| B15-B19 | Viral hepatitis | 1 | 50 (100%) | B18 | Chronic viral hepatitis | 50 (0.02%) |
| B25-B34 | Other viral diseases | 1 | 930 (96.77%) | B26 | Mumps | 15 (0.01%) |
|  |  |  |  | B30 | Viral conjunctivitis | 102 (0.05%) |
|  |  |  |  | B34 | Viral infection of unspecified site | 813 (0.38%) |
|  |  | 2 | 31 (3.23%) | B27 | Infectious mononucleosis | 31 (0.01%) |
| B35-B49 | Mycoses | 1 | 461 (100%) | B35 | Dermatophytosis | 181 (0.08%) |
|  |  |  |  | B36 | Other superficial mycoses | 35 (0.02%) |
|  |  |  |  | B37 | Candidiasis | 154 (0.07%) |
|  |  |  |  | B49 | Unspecified mycosis | 91 (0.04%) |
| B85-B89 | Pediculosis, acariasis and other infestations | 1 | 106 (100%) | B86 | Scabies | 106 (0.05%) |
| B99-B99 | Other infectious diseases | 3 | 96 (100%) | B99 | Other and unspecified infectious diseases | 96 (0.04%) |
| C00-C97 | Malignant neoplasms | 3 | 265 (33.33%) | C11 | Malignant neoplasm of nasopharynx | 46 (0.02%) |
|  |  |  |  | C50 | Malignant neoplasm of breast | 161 (0.07%) |
|  |  |  |  | C61 | Malignant neoplasm of prostate | 27 (0.01%) |
|  |  |  |  | C80 | Malignant neoplasm without specification of site | 31 (0.01%) |
|  |  | 4 | 530 (66.67%) | C15 | Malignant neoplasm of esophagus | 30 (0.01%) |
|  |  |  |  | C16 | Malignant neoplasm of stomach | 45 (0.02%) |
|  |  |  |  | C18 | Malignant neoplasm of colon | 79 (0.04%) |
|  |  |  |  | C20 | Malignant neoplasm of rectum | 31 (0.01%) |
|  |  |  |  | C22 | Malignant neoplasm of liver and intrahepatic bile ducts | 46 (0.02%) |
|  |  |  |  | C25 | Malignant neoplasm of pancreas | 26 (0.01%) |
|  |  |  |  | C34 | Malignant neoplasm of bronchus and lung | 94 (0.04%) |
|  |  |  |  | C64 | Malignant neoplasm of kidney, except renal pelvis | 15 (0.01%) |
|  |  |  |  | C78 | Secondary malignant neoplasm of respiratory and digestive organs | 16 (0.01%) |
|  |  |  |  | C79 | Secondary malignant neoplasm of other and unspecified sites | 58 (0.03%) |
|  |  |  |  | C85 | Other specified and unspecified types of non-Hodgkin lymphoma | 49 (0.02%) |
|  |  |  |  | C92 | Myeloid leukemia | 18 (0.01%) |
|  |  |  |  | C95 | Leukemia of unspecified cell type | 23 (0.01%) |
| D10-D36 | Benign neoplasms | 1 | 167 (100%) | D17 | Benign lipomatous neoplasm | 113 (0.05%) |
|  |  |  |  | D18 | Hemangioma and lymphangioma, any site | 17 (0.01%) |
|  |  |  |  | D25 | Leiomyoma of uterus | 37 (0.02%) |
| D37-D48 | Neoplasms of uncertain or unknown behaviour | 3 | 31 (65.96%) | D48 | Neoplasm of uncertain behavior of other and unspecified sites | 31 (0.01%) |
|  |  | 4 | 16 (34.04%) | D37 | Neoplasm of uncertain behavior of oral cavity and digestive organs | 16 (0.01%) |
| D60-D64 | Aplastic and other anaemias | 3 | 245 (91.08%) | D64 | Other anemias | 245 (0.11%) |
|  |  | 4 | 24 (8.92%) | D61 | Other aplastic anemias and other bone marrow failure syndromes | 24 (0.01%) |
| D65-D69 | Coagulation defects, purpura and other haemorrhagic conditions | 3 | 157 (100%) | D69 | Purpura and other hemorrhagic conditions | 157 (0.07%) |
| D70-D77 | Other diseases of blood and blood-forming organs | 4 | 91 (100%) | D70 | Neutropenia | 45 (0.02%) |
|  |  |  |  | D72 | Other disorders of white blood cells | 24 (0.01%) |
|  |  |  |  | D75 | Other and unspecified diseases of blood and blood-forming organs | 22 (0.01%) |
| E00-E07 | Disorders of thyroid gland | 1 | 70 (26.02%) | E04 | Other nontoxic goiter | 70 (0.03%) |
|  |  | 2 | 199 (73.98%) | E03 | Other hypothyroidism | 37 (0.02%) |
|  |  |  |  | E05 | Thyrotoxicosis [hyperthyroidism] | 137 (0.06%) |
|  |  |  |  | E06 | Thyroiditis | 25 (0.01%) |
| E10-E14 | Diabetes mellitus | 3 | 603 (100%) | E11 | Type 2 diabetes mellitus | 31 (0.01%) |
|  |  |  |  | E14 | Unspecified diabetes mellitus | 572 (0.27%) |
| E15-E16 | Other disorders of glucose regulation and pancreatic internal secretion | 2 | 98 (100%) | E16 | Other disorders of pancreatic internal secretion | 98 (0.05%) |
| E70-E90 | Metabolic disorders | 2 | 124 (14.2%) | E86 | Volume depletion | 124 (0.06%) |
|  |  | 4 | 749 (85.8%) | E83 | Disorders of mineral metabolism | 31 (0.01%) |
|  |  |  |  | E87 | Other disorders of fluid, electrolyte and acid-base balance | 718 (0.33%) |
| F00-F09 | Organic, including symptomatic, mental disorders | 1 | 51 (53.12%) | F07 | Personality and behavioral disorders due to known physiological condition | 51 (0.02%) |
|  |  | 3 | 24 (25%) | F03 | Unspecified dementia | 24 (0.01%) |
|  |  | 4 | 21 (21.88%) | F05 | Delirium due to known physiological condition | 21 (0.01%) |
| F10-F19 | Mental and behavioural disorders due to psychoactive substance use | 1 | 259 (89%) | F10 | Alcohol related disorders | 259 (0.12%) |
|  |  | 2 | 32 (11%) | F19 | Other psychoactive substance related disorders | 32 (0.01%) |
| F20-F29 | Schizophrenia, schizotypal and delusional disorders | 1 | 38 (36.89%) | F20 | Schizophrenia | 38 (0.02%) |
|  |  | 3 | 49 (47.57%) | F29 | Unspecified psychosis not due to a substance or known physiological condition | 49 (0.02%) |
|  |  | 4 | 16 (15.53%) | F22 | Delusional disorders | 16 (0.01%) |
| F30-F39 | Mood [affective] disorders | 2 | 212 (90.21%) | F32 | Major depressive disorder, single episode | 212 (0.1%) |
|  |  | 3 | 23 (9.79%) | F31 | Bipolar disorder | 23 (0.01%) |
| F40-F48 | Neurotic, stress-related and somatoform disorders | 1 | 478 (100%) | F41 | Other anxiety disorders | 291 (0.14%) |
|  |  |  |  | F43 | Reaction to severe stress, and adjustment disorders | 99 (0.05%) |
|  |  |  |  | F45 | Somatoform disorders | 88 (0.04%) |
| F50-F59 | Behavioural syndromes associated with physiological disturbances and physical factors | 1 | 31 (46.97%) | F52 | Sexual dysfunction not due to a substance or known physiological condition | 31 (0.01%) |
|  |  | 3 | 35 (53.03%) | F50 | Eating disorders | 35 (0.02%) |
| G00-G09 | Inflammatory diseases of the central nervous system | 4 | 17 (100%) | G03 | Meningitis due to other and unspecified causes | 17 (0.01%) |
| G20-G26 | Extrapyramidal and movement disorders | 3 | 84 (100%) | G20 | Parkinson's disease | 60 (0.03%) |
|  |  |  |  | G25 | Other extrapyramidal and movement disorders | 24 (0.01%) |
| G35-G37 | Demyelinating diseases of the central nervous system | 4 | 20 (100%) | G35 | Multiple sclerosis | 20 (0.01%) |
| G40-G47 | Episodic and paroxysmal disorders | 1 | 1689 (90.13%) | G40 | Epilepsy and recurrent seizures | 70 (0.03%) |
|  |  |  |  | G43 | Migraine | 879 (0.41%) |
|  |  |  |  | G44 | Other headache syndromes | 391 (0.18%) |
|  |  |  |  | G47 | Sleep disorders | 349 (0.16%) |
|  |  | 4 | 185 (9.87%) | G45 | Transient cerebral ischemic attacks and related syndromes | 185 (0.09%) |
| G50-G59 | Nerve, nerve root and plexus disorders | 1 | 504 (70.19%) | G51 | Facial nerve disorders | 265 (0.12%) |
|  |  |  |  | G54 | Nerve root and plexus disorders | 103 (0.05%) |
|  |  |  |  | G56 | Mononeuropathies of upper limb | 136 (0.06%) |
|  |  | 2 | 143 (19.92%) | G50 | Disorders of trigeminal nerve | 143 (0.07%) |
|  |  | 4 | 71 (9.89%) | G52 | Disorders of other cranial nerves | 71 (0.03%) |
| G60-G64 | Polyneuropathies and other disorders of the peripheral nervous system | 2 | 66 (100%) | G62 | Other and unspecified polyneuropathies | 66 (0.03%) |
| G70-G73 | Diseases of myoneural junction and muscle | 4 | 38 (100%) | G70 | Myasthenia gravis and other myoneural disorders | 38 (0.02%) |
| G80-G83 | Cerebral palsy and other paralytic syndromes | 4 | 78 (100%) | G81 | Hemiplegia and hemiparesis | 17 (0.01%) |
|  |  |  |  | G83 | Other paralytic syndromes | 61 (0.03%) |
| G90-G99 | Other disorders of the nervous system | 2 | 109 (81.95%) | G95 | Other and unspecified diseases of spinal cord | 88 (0.04%) |
|  |  |  |  | G99 | Other disorders of nervous system in diseases classified elsewhere | 21 (0.01%) |
|  |  | 4 | 24 (18.05%) | G93 | Other disorders of brain | 24 (0.01%) |
| H00-H06 | Disorders of eyelid, lacrimal system and orbit | 1 | 575 (80.99%) | H00 | Hordeolum and chalazion | 346 (0.16%) |
|  |  |  |  | H01 | Other inflammation of eyelid | 68 (0.03%) |
|  |  |  |  | H04 | Disorders of lacrimal system | 161 (0.07%) |
|  |  | 2 | 89 (12.54%) | H02 | Other disorders of eyelid | 89 (0.04%) |
|  |  | 3 | 46 (6.48%) | H05 | Disorders of orbit | 46 (0.02%) |
| H10-H13 | Disorders of conjunctiva | 1 | 1890 (100%) | H10 | Conjunctivitis | 1205 (0.56%) |
|  |  |  |  | H11 | Other disorders of conjunctiva | 685 (0.32%) |
| H15-H22 | Disorders of sclera, cornea, iris and ciliary body | 1 | 256 (48.3%) | H15 | Disorders of sclera | 56 (0.03%) |
|  |  |  |  | H17 | Corneal scars and opacities | 63 (0.03%) |
|  |  |  |  | H18 | Other disorders of cornea | 67 (0.03%) |
|  |  |  |  | H20 | Iridocyclitis | 70 (0.03%) |
|  |  | 2 | 274 (51.7%) | H16 | Keratitis | 274 (0.13%) |
| H25-H28 | Disorders of lens | 1 | 124 (100%) | H26 | Other cataract | 124 (0.06%) |
| H30-H36 | Disorders of choroid and retina | 1 | 230 (83.64%) | H33 | Retinal detachments and breaks | 214 (0.1%) |
|  |  |  |  | H35 | Other retinal disorders | 16 (0.01%) |
|  |  | 3 | 45 (16.36%) | H34 | Retinal vascular occlusions | 45 (0.02%) |
| H40-H42 | Glaucoma | 2 | 123 (100%) | H40 | Glaucoma | 123 (0.06%) |
| H43-H45 | Disorders of vitreous body and globe | 1 | 564 (100%) | H43 | Disorders of vitreous body | 236 (0.11%) |
|  |  |  |  | H44 | Disorders of globe | 328 (0.15%) |
| H46-H48 | Disorders of optic nerve and visual pathways | 4 | 46 (100%) | H46 | Optic neuritis | 24 (0.01%) |
|  |  |  |  | H47 | Other disorders of optic [2nd] nerve and visual pathways | 22 (0.01%) |
| H49-H52 | Disorders of ocular muscles, binocular movement, accommodation and refraction | 1 | 16 (15.53%) | H52 | Disorders of refraction and accommodation | 16 (0.01%) |
|  |  | 4 | 87 (84.47%) | H49 | Paralytic strabismus | 87 (0.04%) |
| H53-H54 | Visual disturbances and blindness | 2 | 1091 (100%) | H53 | Visual disturbances | 934 (0.43%) |
|  |  |  |  | H54 | Blindness and low vision | 157 (0.07%) |
| H55-H59 | Other disorders of eye and adnexa | 1 | 1059 (100%) | H57 | Other disorders of eye and adnexa | 1059 (0.49%) |
| H60-H62 | Diseases of external ear | 1 | 1601 (100%) | H60 | Otitis externa | 1208 (0.56%) |
|  |  |  |  | H61 | Other disorders of external ear | 393 (0.18%) |
| H65-H75 | Diseases of middle ear and mastoid | 1 | 961 (100%) | H65 | Nonsuppurative otitis media | 34 (0.02%) |
|  |  |  |  | H66 | Suppurative and unspecified otitis media | 747 (0.35%) |
|  |  |  |  | H69 | Other and unspecified disorders of Eustachian tube | 36 (0.02%) |
|  |  |  |  | H72 | Perforation of tympanic membrane | 144 (0.07%) |
| H80-H83 | Diseases of inner ear | 1 | 622 (100%) | H81 | Disorders of vestibular function | 606 (0.28%) |
|  |  |  |  | H83 | Other diseases of inner ear | 16 (0.01%) |
| H90-H95 | Other disorders of ear | 1 | 1192 (100%) | H90 | Conductive and sensorineural hearing loss | 101 (0.05%) |
|  |  |  |  | H91 | Other and unspecified hearing loss | 283 (0.13%) |
|  |  |  |  | H92 | Otalgia and effusion of ear | 403 (0.19%) |
|  |  |  |  | H93 | Other disorders of ear, not elsewhere classified | 405 (0.19%) |
| I00-I99 | Diseases of the circulatory system | 1 | 2129 (39.4%) | I10 | Essential (primary) hypertension | 1597 (0.74%) |
|  |  |  |  | I24 | Other acute ischemic heart diseases | 20 (0.01%) |
|  |  |  |  | I47 | Paroxysmal tachycardia | 62 (0.03%) |
|  |  |  |  | I48 | Atrial fibrillation and flutter | 264 (0.12%) |
|  |  |  |  | I49 | Other cardiac arrhythmias | 37 (0.02%) |
|  |  |  |  | I86 | Varicose veins of other sites | 26 (0.01%) |
|  |  |  |  | I87 | Other disorders of veins | 123 (0.06%) |
|  |  | 2 | 1854 (34.31%) | I20 | Angina pectoris | 265 (0.12%) |
|  |  |  |  | I25 | Chronic ischemic heart disease | 57 (0.03%) |
|  |  |  |  | I83 | Varicose veins of lower extremities | 309 (0.14%) |
|  |  |  |  | I84 | Haemorrhoids | 1111 (0.52%) |
|  |  |  |  | I88 | Nonspecific lymphadenitis | 112 (0.05%) |
|  |  | 3 | 181 (3.35%) | I21 | Acute myocardial infarction | 68 (0.03%) |
|  |  |  |  | I82 | Other venous embolism and thrombosis | 16 (0.01%) |
|  |  |  |  | I89 | Other noninfective disorders of lymphatic vessels and lymph nodes | 57 (0.03%) |
|  |  |  |  | I95 | Hypotension | 40 (0.02%) |
|  |  | 4 | 1240 (22.95%) | I50 | Heart failure | 79 (0.04%) |
|  |  |  |  | I67 | Other cerebrovascular diseases | 189 (0.09%) |
|  |  |  |  | I73 | Other peripheral vascular diseases | 237 (0.11%) |
|  |  |  |  | I77 | Other disorders of arteries and arterioles | 144 (0.07%) |
|  |  |  |  | I80 | Phlebitis and thrombophlebitis | 270 (0.13%) |
|  |  |  |  | I96 | Gangrene, not elsewhere classified | 321 (0.15%) |
| J00-J06 | Acute upper respiratory infections | 1 | 18685 (100%) | J01 | Acute sinusitis | 42 (0.02%) |
|  |  |  |  | J02 | Acute pharyngitis | 848 (0.39%) |
|  |  |  |  | J03 | Acute tonsillitis | 1600 (0.74%) |
|  |  |  |  | J04 | Acute laryngitis and tracheitis | 35 (0.02%) |
|  |  |  |  | J06 | Acute upper respiratory infections of multiple and unspecified sites | 16160 (7.5%) |
| J09-J18 | Influenza and pneumonia | 1 | 72 (2.66%) | J11 | Influenza due to unidentified influenza virus | 72 (0.03%) |
|  |  | 3 | 66 (2.44%) | J15 | Bacterial pneumonia, not elsewhere classified | 66 (0.03%) |
|  |  | 4 | 2572 (94.91%) | J18 | Pneumonia, unspecified organism | 2572 (1.19%) |
| J20-J22 | Other acute lower respiratory infections | 2 | 126 (7.45%) | J20 | Acute bronchitis | 126 (0.06%) |
|  |  | 3 | 1565 (92.55%) | J22 | Unspecified acute lower respiratory infection | 1565 (0.73%) |
| J30-J39 | Other diseases of upper respiratory tract | 1 | 1215 (91.01%) | J30 | Vasomotor and allergic rhinitis | 137 (0.06%) |
|  |  |  |  | J31 | Chronic rhinitis, nasopharyngitis and pharyngitis | 146 (0.07%) |
|  |  |  |  | J32 | Chronic sinusitis | 425 (0.2%) |
|  |  |  |  | J33 | Nasal polyp | 18 (0.01%) |
|  |  |  |  | J34 | Other and unspecified disorders of nose and nasal sinuses | 164 (0.08%) |
|  |  |  |  | J35 | Chronic diseases of tonsils and adenoids | 48 (0.02%) |
|  |  |  |  | J39 | Other diseases of upper respiratory tract | 277 (0.13%) |
|  |  | 2 | 120 (8.99%) | J36 | Peritonsillar abscess | 120 (0.06%) |
| J40-J47 | Chronic lower respiratory diseases | 1 | 1073 (60.15%) | J45 | Asthma | 1073 (0.5%) |
|  |  | 2 | 571 (32.01%) | J40 | Bronchitis, not specified as acute or chronic | 458 (0.21%) |
|  |  |  |  | J44 | Other chronic obstructive pulmonary disease | 113 (0.05%) |
|  |  | 4 | 140 (7.85%) | J47 | Bronchiectasis | 140 (0.06%) |
| J90-J94 | Other diseases of pleura | 1 | 113 (49.56%) | J93 | Pneumothorax and air leak | 113 (0.05%) |
|  |  | 4 | 115 (50.44%) | J90 | Pleural effusion, not elsewhere classified | 115 (0.05%) |
| J95-J99 | Other diseases of the respiratory system | 2 | 760 (100%) | J98 | Other respiratory disorders | 760 (0.35%) |
| K00-K14 | Diseases of oral cavity, salivary glands and jaws | 1 | 1807 (79.74%) | K01 | Embedded and impacted teeth | 31 (0.01%) |
|  |  |  |  | K02 | Dental caries | 125 (0.06%) |
|  |  |  |  | K04 | Diseases of pulp and periapical tissues | 165 (0.08%) |
|  |  |  |  | K05 | Gingivitis and periodontal diseases | 129 (0.06%) |
|  |  |  |  | K06 | Other disorders of gingiva and edentulous alveolar ridge | 124 (0.06%) |
|  |  |  |  | K07 | Dentofacial anomalies [including malocclusion] and mandibular deformities | 72 (0.03%) |
|  |  |  |  | K08 | Other disorders of teeth and supporting structures | 949 (0.44%) |
|  |  |  |  | K10 | Other diseases of jaws | 105 (0.05%) |
|  |  |  |  | K13 | Other diseases of lip and oral mucosa | 44 (0.02%) |
|  |  |  |  | K14 | Diseases of tongue | 63 (0.03%) |
|  |  | 2 | 459 (20.26%) | K11 | Diseases of salivary glands | 212 (0.1%) |
|  |  |  |  | K12 | Stomatitis and related lesions | 247 (0.11%) |
| K20-K31 | Diseases of oesophagus, stomach and duodenum | 1 | 3708 (98.33%) | K21 | Gastro-esophageal reflux disease | 761 (0.35%) |
|  |  |  |  | K29 | Gastritis and duodenitis | 2350 (1.09%) |
|  |  |  |  | K30 | Functional dyspepsia | 597 (0.28%) |
|  |  | 2 | 63 (1.67%) | K22 | Other diseases of esophagus | 63 (0.03%) |
| K35-K38 | Diseases of appendix | 4 | 502 (100%) | K35 | Acute appendicitis | 259 (0.12%) |
|  |  |  |  | K37 | Unspecified appendicitis | 243 (0.11%) |
| K40-K46 | Hernia | 2 | 408 (100%) | K40 | Inguinal hernia | 261 (0.12%) |
|  |  |  |  | K42 | Umbilical hernia | 21 (0.01%) |
|  |  |  |  | K43 | Ventral hernia | 18 (0.01%) |
|  |  |  |  | K46 | Unspecified abdominal hernia | 108 (0.05%) |
| K50-K52 | Noninfective enteritis and colitis | 3 | 66 (70.97%) | K52 | Other and unspecified noninfective gastroenteritis and colitis | 66 (0.03%) |
|  |  | 4 | 27 (29.03%) | K50 | Crohn's disease [regional enteritis] | 27 (0.01%) |
| K55-K64 | Other diseases of intestines | 1 | 1581 (46.51%) | K59 | Other functional intestinal disorders | 1581 (0.73%) |
|  |  | 2 | 236 (6.94%) | K58 | Irritable bowel syndrome | 67 (0.03%) |
|  |  |  |  | K60 | Fissure and fistula of anal and rectal regions | 146 (0.07%) |
|  |  |  |  | K63 | Other diseases of intestine | 23 (0.01%) |
|  |  | 3 | 313 (9.21%) | K62 | Other diseases of anus and rectum | 313 (0.15%) |
|  |  | 4 | 1269 (37.33%) | K56 | Paralytic ileus and intestinal obstruction without hernia | 273 (0.13%) |
|  |  |  |  | K57 | Diverticular disease of intestine | 86 (0.04%) |
|  |  |  |  | K61 | Abscess of anal and rectal regions | 910 (0.42%) |
| K65-K67 | Diseases of peritoneum | 2 | 26 (39.39%) | K66 | Other disorders of peritoneum | 26 (0.01%) |
|  |  | 4 | 40 (60.61%) | K65 | Peritonitis | 40 (0.02%) |
| K70-K77 | Diseases of liver | 3 | 39 (23.21%) | K76 | Other diseases of liver | 39 (0.02%) |
|  |  | 4 | 129 (76.79%) | K74 | Fibrosis and cirrhosis of liver | 29 (0.01%) |
|  |  |  |  | K75 | Other inflammatory liver diseases | 100 (0.05%) |
| K80-K87 | Disorders of gallbladder, biliary tract and pancreas | 2 | 351 (37.82%) | K80 | Cholelithiasis | 351 (0.16%) |
|  |  | 4 | 577 (62.18%) | K81 | Cholecystitis | 295 (0.14%) |
|  |  |  |  | K83 | Other diseases of biliary tract | 193 (0.09%) |
|  |  |  |  | K85 | Acute pancreatitis | 89 (0.04%) |
| K90-K93 | Other diseases of the digestive system | 4 | 158 (100%) | K92 | Other diseases of digestive system | 158 (0.07%) |
| L00-L08 | Infections of the skin and subcutaneous tissue | 2 | 62 (0.58%) | L01 | Impetigo | 41 (0.02%) |
|  |  |  |  | L04 | Acute lymphadenitis | 21 (0.01%) |
|  |  | 3 | 10630 (99.42%) | L02 | Cutaneous abscess, furuncle and carbuncle | 4693 (2.18%) |
|  |  |  |  | L03 | Cellulitis and acute lymphangitis | 5111 (2.37%) |
|  |  |  |  | L05 | Pilonidal cyst and sinus | 145 (0.07%) |
|  |  |  |  | L08 | Other local infections of skin and subcutaneous tissue | 681 (0.32%) |
| L10-L14 | Bullous disorders | 4 | 38 (100%) | L12 | Pemphigoid | 38 (0.02%) |
| L20-L30 | Dermatitis and eczema | 1 | 599 (37.63%) | L20 | Atopic dermatitis | 148 (0.07%) |
|  |  |  |  | L21 | Seborrheic dermatitis | 18 (0.01%) |
|  |  |  |  | L23 | Allergic contact dermatitis | 16 (0.01%) |
|  |  |  |  | L24 | Irritant contact dermatitis | 16 (0.01%) |
|  |  |  |  | L25 | Unspecified contact dermatitis | 169 (0.08%) |
|  |  |  |  | L29 | Pruritus | 232 (0.11%) |
|  |  | 2 | 928 (58.29%) | L28 | Lichen simplex chronicus and prurigo | 24 (0.01%) |
|  |  |  |  | L30 | Other and unspecified dermatitis | 904 (0.42%) |
|  |  | 3 | 65 (4.08%) | L27 | Dermatitis due to substances taken internally | 65 (0.03%) |
| L40-L45 | Papulosquamous disorders | 2 | 110 (100%) | L40 | Psoriasis | 110 (0.05%) |
| L50-L54 | Urticaria and erythema | 1 | 1139 (94.13%) | L50 | Urticaria | 1139 (0.53%) |
|  |  | 3 | 43 (3.55%) | L52 | Erythema nodosum | 15 (0.01%) |
|  |  |  |  | L53 | Other erythematous conditions | 28 (0.01%) |
|  |  | 4 | 28 (2.31%) | L51 | Erythema multiforme | 28 (0.01%) |
| L55-L59 | Radiation-related disorders of the skin and subcutaneous tissue | 1 | 30 (100%) | L55 | Sunburn | 30 (0.01%) |
| L60-L75 | Disorders of skin appendages | 1 | 666 (70.63%) | L60 | Nail disorders | 444 (0.21%) |
|  |  |  |  | L70 | Acne | 38 (0.02%) |
|  |  |  |  | L73 | Other follicular disorders | 155 (0.07%) |
|  |  |  |  | L74 | Eccrine sweat disorders | 29 (0.01%) |
|  |  | 2 | 277 (29.37%) | L72 | Follicular cysts of skin and subcutaneous tissue | 277 (0.13%) |
| L80-L99 | Other disorders of the skin and subcutaneous tissue | 1 | 563 (48.7%) | L81 | Other disorders of pigmentation | 19 (0.01%) |
|  |  |  |  | L84 | Corns and callosities | 157 (0.07%) |
|  |  |  |  | L91 | Hypertrophic disorders of skin | 51 (0.02%) |
|  |  |  |  | L98 | Other disorders of skin and subcutaneous tissue, not elsewhere classified | 336 (0.16%) |
|  |  | 3 | 555 (48.01%) | L97 | Non-pressure chronic ulcer of lower limb, not elsewhere classified | 555 (0.26%) |
|  |  | 4 | 38 (3.29%) | L89 | Pressure ulcer | 38 (0.02%) |
| M00-M25 | Arthropathies | 1 | 6550 (78.52%) | M16 | Osteoarthritis of hip | 55 (0.03%) |
|  |  |  |  | M17 | Osteoarthritis of knee | 701 (0.33%) |
|  |  |  |  | M19 | Other and unspecified osteoarthritis | 828 (0.38%) |
|  |  |  |  | M20 | Acquired deformities of fingers and toes | 126 (0.06%) |
|  |  |  |  | M22 | Disorder of patella | 17 (0.01%) |
|  |  |  |  | M23 | Internal derangement of knee | 148 (0.07%) |
|  |  |  |  | M24 | Other specific joint derangements | 22 (0.01%) |
|  |  |  |  | M25 | Other joint disorder, not elsewhere classified | 4653 (2.16%) |
|  |  | 2 | 1543 (18.5%) | M10 | Gout | 1295 (0.6%) |
|  |  |  |  | M11 | Other crystal arthropathies | 16 (0.01%) |
|  |  |  |  | M13 | Other arthritis | 193 (0.09%) |
|  |  |  |  | M21 | Other acquired deformities of limbs | 39 (0.02%) |
|  |  | 3 | 184 (2.21%) | M06 | Other rheumatoid arthritis | 184 (0.09%) |
|  |  | 4 | 65 (0.78%) | M00 | Pyogenic arthritis | 65 (0.03%) |
| M30-M36 | Systemic connective tissue disorders | 3 | 53 (55.79%) | M32 | Systemic lupus erythematosus (SLE) | 53 (0.02%) |
|  |  | 4 | 42 (44.21%) | M35 | Other systemic involvement of connective tissue | 42 (0.02%) |
| M40-M54 | Dorsopathies | 1 | 12195 (99.49%) | M41 | Scoliosis | 19 (0.01%) |
|  |  |  |  | M43 | Other deforming dorsopathies | 114 (0.05%) |
|  |  |  |  | M47 | Spondylosis | 1231 (0.57%) |
|  |  |  |  | M48 | Other spondylopathies | 113 (0.05%) |
|  |  |  |  | M51 | Thoracic, thoracolumbar, and lumbosacral intervertebral disc disorders | 590 (0.27%) |
|  |  |  |  | M53 | Other and unspecified dorsopathies, not elsewhere classified | 41 (0.02%) |
|  |  |  |  | M54 | Dorsalgia | 10087 (4.68%) |
|  |  | 2 | 63 (0.51%) | M45 | Ankylosing spondylitis | 18 (0.01%) |
|  |  |  |  | M46 | Other inflammatory spondylopathies | 45 (0.02%) |
| M60-M79 | Soft tissue disorders | 1 | 5197 (91.61%) | M66 | Spontaneous rupture of synovium and tendon | 45 (0.02%) |
|  |  |  |  | M67 | Other disorders of synovium and tendon | 69 (0.03%) |
|  |  |  |  | M70 | Soft tissue disorders related to use, overuse and pressure | 62 (0.03%) |
|  |  |  |  | M71 | Other bursopathies | 117 (0.05%) |
|  |  |  |  | M72 | Fibroblastic disorders | 255 (0.12%) |
|  |  |  |  | M75 | Shoulder lesions | 449 (0.21%) |
|  |  |  |  | M76 | Enthesopathies, lower limb, excluding foot | 114 (0.05%) |
|  |  |  |  | M77 | Other enthesopathies | 283 (0.13%) |
|  |  |  |  | M79 | Other and unspecified soft tissue disorders, not elsewhere classified | 3803 (1.76%) |
|  |  | 2 | 334 (5.89%) | M65 | Synovitis and tenosynovitis | 334 (0.16%) |
|  |  | 3 | 19 (0.33%) | M60 | Myositis | 19 (0.01%) |
|  |  | 4 | 123 (2.17%) | M62 | Other disorders of muscle | 123 (0.06%) |
| M80-M94 | Osteopathies and chondropathies | 1 | 95 (28.79%) | M92 | Other juvenile osteochondrosis | 16 (0.01%) |
|  |  |  |  | M94 | Other disorders of cartilage | 79 (0.04%) |
|  |  | 2 | 57 (17.27%) | M84 | Disorder of continuity of bone | 31 (0.01%) |
|  |  |  |  | M89 | Other disorders of bone | 26 (0.01%) |
|  |  | 4 | 178 (53.94%) | M86 | Osteomyelitis | 178 (0.08%) |
| N00-N08 | Glomerular diseases | 4 | 37 (100%) | N04 | Nephrotic syndrome | 37 (0.02%) |
| N10-N16 | Renal tubulo-interstitial diseases | 2 | 39 (5.08%) | N13 | Obstructive and reflux uropathy | 39 (0.02%) |
|  |  | 3 | 729 (94.92%) | N10 | Acute pyelonephritis | 220 (0.1%) |
|  |  |  |  | N12 | Tubulo-interstitial nephritis, not specified as acute or chronic | 509 (0.24%) |
| N17-N19 | Renal failure | 3 | 23 (5.56%) | N19 | Unspecified kidney failure | 23 (0.01%) |
|  |  | 4 | 391 (94.44%) | N17 | Acute kidney failure | 54 (0.03%) |
|  |  |  |  | N18 | Chronic kidney disease (CKD) | 337 (0.16%) |
| N20-N23 | Urolithiasis | 1 | 2336 (100%) | N20 | Calculus of kidney and ureter | 234 (0.11%) |
|  |  |  |  | N21 | Calculus of lower urinary tract | 19 (0.01%) |
|  |  |  |  | N23 | Unspecified renal colic | 2083 (0.97%) |
| N30-N39 | Other diseases of urinary system | 1 | 279 (6.84%) | N30 | Cystitis | 209 (0.1%) |
|  |  |  |  | N32 | Other disorders of bladder | 16 (0.01%) |
|  |  |  |  | N34 | Urethritis and urethral syndrome | 54 (0.03%) |
|  |  | 2 | 3798 (93.16%) | N39 | Other disorders of urinary system | 3798 (1.76%) |
| N40-N51 | Diseases of male genital organs | 1 | 1084 (93.13%) | N40 | Benign prostatic hyperplasia | 165 (0.08%) |
|  |  |  |  | N43 | Hydrocele and spermatocele | 20 (0.01%) |
|  |  |  |  | N45 | Orchitis and epididymitis | 380 (0.18%) |
|  |  |  |  | N47 | Disorders of prepuce | 102 (0.05%) |
|  |  |  |  | N48 | Other disorders of penis | 145 (0.07%) |
|  |  |  |  | N50 | Other and unspecified disorders of male genital organs | 272 (0.13%) |
|  |  | 2 | 44 (3.78%) | N41 | Inflammatory diseases of prostate | 24 (0.01%) |
|  |  |  |  | N44 | Noninflammatory disorders of testis | 20 (0.01%) |
|  |  | 3 | 36 (3.09%) | N49 | Inflammatory disorders of male genital organs, not elsewhere classified | 36 (0.02%) |
| N60-N64 | Disorders of breast | 1 | 181 (20.38%) | N64 | Other disorders of breast | 181 (0.08%) |
|  |  | 2 | 393 (44.26%) | N63 | Unspecified lump in breast | 393 (0.18%) |
|  |  | 3 | 314 (35.36%) | N61 | Inflammatory disorders of breast | 314 (0.15%) |
| N70-N77 | Inflammatory diseases of female pelvic organs | 2 | 346 (100%) | N73 | Other female pelvic inflammatory diseases | 104 (0.05%) |
|  |  |  |  | N75 | Diseases of Bartholin's gland | 131 (0.06%) |
|  |  |  |  | N76 | Other inflammation of vagina and vulva | 111 (0.05%) |
| N80-N98 | Noninflammatory disorders of female genital tract | 1 | 818 (87.02%) | N81 | Female genital prolapse | 58 (0.03%) |
|  |  |  |  | N89 | Other noninflammatory disorders of vagina | 65 (0.03%) |
|  |  |  |  | N90 | Other noninflammatory disorders of vulva and perineum | 22 (0.01%) |
|  |  |  |  | N91 | Absent, scanty and rare menstruation | 27 (0.01%) |
|  |  |  |  | N92 | Excessive, frequent and irregular menstruation | 216 (0.1%) |
|  |  |  |  | N93 | Other abnormal uterine and vaginal bleeding | 143 (0.07%) |
|  |  |  |  | N94 | Pain and other conditions associated with female genital organs and menstrual cycle | 232 (0.11%) |
|  |  |  |  | N95 | Menopausal and other perimenopausal disorders | 55 (0.03%) |
|  |  | 2 | 30 (3.19%) | N80 | Endometriosis | 30 (0.01%) |
|  |  | 3 | 92 (9.79%) | N83 | Noninflammatory disorders of ovary, fallopian tube and broad ligament | 92 (0.04%) |
| O00-O08 | Pregnancy with abortive outcome | 2 | 15 (100%) | O00 | Ectopic pregnancy | 15 (0.01%) |
| O20-O29 | Other maternal disorders predominantly related to pregnancy | 1 | 53 (30.46%) | O20 | Hemorrhage in early pregnancy | 53 (0.02%) |
|  |  | 2 | 121 (69.54%) | O21 | Excessive vomiting in pregnancy | 121 (0.06%) |
| P10-P15 | Birth trauma | 1 | 51 (100%) | P12 | Birth injury to scalp | 51 (0.02%) |
| P50-P61 | Haemorrhagic and haematological disorders of fetus and newborn | 3 | 41 (100%) | P59 | Neonatal jaundice from other and unspecified causes | 41 (0.02%) |
| Q10-Q18 | Congenital malformations of eye, ear, face and neck | 1 | 21 (100%) | Q18 | Other congenital malformations of face and neck | 21 (0.01%) |
| Q60-Q64 | Congenital malformations of the urinary system | 3 | 21 (100%) | Q61 | Cystic kidney disease | 21 (0.01%) |
| R00-R09 | Symptoms and signs involving the circulatory and respiratory systems | 1 | 10608 (82.86%) | R00 | Abnormalities of heart beat | 649 (0.3%) |
|  |  |  |  | R05 | Cough | 1333 (0.62%) |
|  |  |  |  | R07 | Pain in throat and chest | 8362 (3.88%) |
|  |  |  |  | R09 | Other symptoms and signs involving the circulatory and respiratory system | 264 (0.12%) |
|  |  | 2 | 673 (5.26%) | R06 | Abnormalities of breathing | 673 (0.31%) |
|  |  | 3 | 1522 (11.89%) | R04 | Hemorrhage from respiratory passages | 1522 (0.71%) |
| R10-R19 | Symptoms and signs involving the digestive system and abdomen | 2 | 5772 (84.81%) | R10 | Abdominal and pelvic pain | 4985 (2.31%) |
|  |  |  |  | R11 | Nausea and vomiting | 656 (0.3%) |
|  |  |  |  | R14 | Flatulence and related conditions | 131 (0.06%) |
|  |  | 3 | 784 (11.52%) | R13 | Aphagia and dysphagia | 189 (0.09%) |
|  |  |  |  | R16 | Hepatomegaly and splenomegaly, not elsewhere classified | 23 (0.01%) |
|  |  |  |  | R19 | Other symptoms and signs involving the digestive system and abdomen | 572 (0.27%) |
|  |  | 4 | 250 (3.67%) | R17 | Unspecified jaundice | 197 (0.09%) |
|  |  |  |  | R18 | Ascites | 53 (0.02%) |
| R20-R23 | Symptoms and signs involving the skin and subcutaneous tissue | 2 | 2629 (100%) | R20 | Disturbances of skin sensation | 392 (0.18%) |
|  |  |  |  | R21 | Rash and other nonspecific skin eruption | 1310 (0.61%) |
|  |  |  |  | R22 | Localized swelling, mass and lump of skin and subcutaneous tissue | 848 (0.39%) |
|  |  |  |  | R23 | Other skin changes | 79 (0.04%) |
| R25-R29 | Symptoms and signs involving the nervous and musculoskeletal systems | 1 | 2238 (89.77%) | R29 | Other symptoms and signs involving the nervous and musculoskeletal systems | 2238 (1.04%) |
|  |  | 2 | 204 (8.18%) | R25 | Abnormal involuntary movements | 204 (0.09%) |
|  |  | 3 | 51 (2.05%) | R26 | Abnormalities of gait and mobility | 51 (0.02%) |
| R30-R39 | Symptoms and signs involving the urinary system | 1 | 584 (38.19%) | R30 | Pain associated with micturition | 125 (0.06%) |
|  |  |  |  | R33 | Retention of urine | 270 (0.13%) |
|  |  |  |  | R35 | Polyuria | 68 (0.03%) |
|  |  |  |  | R36 | Urethral discharge | 32 (0.01%) |
|  |  |  |  | R39 | Other and unspecified symptoms and signs involving the genitourinary system | 89 (0.04%) |
|  |  | 2 | 945 (61.81%) | R31 | Hematuria | 877 (0.41%) |
|  |  |  |  | R32 | Unspecified urinary incontinence | 68 (0.03%) |
| R40-R46 | Symptoms and signs involving cognition, perception, emotional state and behaviour | 1 | 3537 (90.67%) | R42 | Dizziness and giddiness | 3502 (1.63%) |
|  |  |  |  | R46 | Symptoms and signs involving appearance and behavior | 35 (0.02%) |
|  |  | 2 | 102 (2.61%) | R43 | Disturbances of smell and taste | 23 (0.01%) |
|  |  |  |  | R45 | Symptoms and signs involving emotional state | 79 (0.04%) |
|  |  | 3 | 64 (1.64%) | R40 | Somnolence, stupor and coma | 30 (0.01%) |
|  |  |  |  | R44 | Other symptoms and signs involving general sensations and perceptions | 34 (0.02%) |
|  |  | 4 | 198 (5.08%) | R41 | Other symptoms and signs involving cognitive functions and awareness | 198 (0.09%) |
| R47-R49 | Symptoms and signs involving speech and voice | 1 | 32 (100%) | R49 | Voice and resonance disorders | 32 (0.01%) |
| R50-R69 | General symptoms and signs | 1 | 2868 (20.93%) | R52 | Pain, unspecified | 1456 (0.68%) |
|  |  |  |  | R56 | Convulsions, not elsewhere classified | 212 (0.1%) |
|  |  |  |  | R61 | Generalized hyperhidrosis | 39 (0.02%) |
|  |  |  |  | R68 | Other general symptoms and signs | 1161 (0.54%) |
|  |  | 2 | 4574 (33.38%) | R51 | Headache | 3370 (1.56%) |
|  |  |  |  | R55 | Syncope and collapse | 474 (0.22%) |
|  |  |  |  | R58 | Hemorrhage, not elsewhere classified | 158 (0.07%) |
|  |  |  |  | R59 | Enlarged lymph nodes | 292 (0.14%) |
|  |  |  |  | R60 | Edema, not elsewhere classified | 280 (0.13%) |
|  |  | 3 | 6262 (45.69%) | R50 | Fever of other and unknown origin | 5638 (2.62%) |
|  |  |  |  | R53 | Malaise and fatigue | 322 (0.15%) |
|  |  |  |  | R63 | Symptoms and signs concerning food and fluid intake | 302 (0.14%) |
| R70-R79 | Abnormal findings on examination of blood, without diagnosis | 2 | 209 (100%) | R73 | Elevated blood glucose level | 190 (0.09%) |
|  |  |  |  | R79 | Other abnormal findings of blood chemistry | 19 (0.01%) |
| R80-R82 | Abnormal findings on examination of urine, without diagnosis | 2 | 18 (100%) | R80 | Proteinuria | 18 (0.01%) |
| R90-R94 | Abnormal findings on diagnostic imaging/in function studies, without diagnosis | 3 | 159 (100%) | R91 | Abnormal findings on diagnostic imaging of lung | 102 (0.05%) |
|  |  |  |  | R94 | Abnormal results of function studies | 57 (0.03%) |
| S00-S09 | Injuries to the head | 1 | 7197 (100%) | S00 | Superficial injury of head | 1059 (0.49%) |
|  |  |  |  | S01 | Open wound of head | 1558 (0.72%) |
|  |  |  |  | S02 | Fracture of skull and facial bones | 423 (0.2%) |
|  |  |  |  | S03 | Dislocation and sprain of joints and ligaments of head | 35 (0.02%) |
|  |  |  |  | S05 | Injury of eye and orbit | 298 (0.14%) |
|  |  |  |  | S06 | Intracranial injury | 66 (0.03%) |
|  |  |  |  | S09 | Other and unspecified injuries of head | 3758 (1.74%) |
| S10-S19 | Injuries to the neck | 1 | 644 (100%) | S10 | Superficial injury of neck | 51 (0.02%) |
|  |  |  |  | S13 | Dislocation and sprain of joints and ligaments at neck level | 441 (0.2%) |
|  |  |  |  | S16 | Injury of muscle, fascia and tendon at neck level | 152 (0.07%) |
| S20-S29 | Injuries to the thorax | 1 | 550 (100%) | S20 | Superficial injury of thorax | 270 (0.13%) |
|  |  |  |  | S22 | Fracture of rib(s), sternum and thoracic spine | 265 (0.12%) |
|  |  |  |  | S29 | Other and unspecified injuries of thorax | 15 (0.01%) |
| S30-S39 | Injuries to the abdomen, lower back, lumbar spine and pelvis | 1 | 503 (97.1%) | S30 | Superficial injury of abdomen, lower back, pelvis and external genitals | 231 (0.11%) |
|  |  |  |  | S32 | Fracture of lumbar spine and pelvis | 162 (0.08%) |
|  |  |  |  | S33 | Dislocation and sprain of joints and ligaments of lumbar spine and pelvis | 89 (0.04%) |
|  |  |  |  | S39 | Other and unspecified injuries of abdomen, lower back, pelvis and external genitals | 21 (0.01%) |
|  |  | 2 | 15 (2.9%) | S31 | Open wound of abdomen, lower back, pelvis and external genitals | 15 (0.01%) |
| S40-S49 | Injuries to the shoulder and upper arm | 1 | 1008 (100%) | S40 | Superficial injury of shoulder and upper arm | 259 (0.12%) |
|  |  |  |  | S42 | Fracture of shoulder and upper arm | 408 (0.19%) |
|  |  |  |  | S43 | Dislocation and sprain of joints and ligaments of shoulder girdle | 228 (0.11%) |
|  |  |  |  | S46 | Injury of muscle, fascia and tendon at shoulder and upper arm level | 83 (0.04%) |
|  |  |  |  | S49 | Other and unspecified injuries of shoulder and upper arm | 30 (0.01%) |
| S50-S59 | Injuries to the elbow and forearm | 1 | 1250 (90.65%) | S50 | Superficial injury of elbow and forearm | 196 (0.09%) |
|  |  |  |  | S52 | Fracture of forearm | 946 (0.44%) |
|  |  |  |  | S53 | Dislocation and sprain of joints and ligaments of elbow | 57 (0.03%) |
|  |  |  |  | S59 | Other and unspecified injuries of elbow and forearm | 51 (0.02%) |
|  |  | 2 | 107 (7.76%) | S51 | Open wound of elbow and forearm | 107 (0.05%) |
|  |  | 4 | 22 (1.6%) | S56 | Injury of muscle, fascia and tendon at forearm level | 22 (0.01%) |
| S60-S69 | Injuries to the wrist and hand | 1 | 2130 (45.53%) | S60 | Superficial injury of wrist, hand and fingers | 561 (0.26%) |
|  |  |  |  | S62 | Fracture at wrist and hand level | 1265 (0.59%) |
|  |  |  |  | S63 | Dislocation and sprain of joints and ligaments at wrist and hand level | 273 (0.13%) |
|  |  |  |  | S66 | Injury of muscle, fascia and tendon at wrist and hand level | 31 (0.01%) |
|  |  | 2 | 2508 (53.61%) | S61 | Open wound of wrist, hand and fingers | 1744 (0.81%) |
|  |  |  |  | S67 | Crushing injury of wrist, hand and fingers | 50 (0.02%) |
|  |  |  |  | S69 | Other and unspecified injuries of wrist, hand and finger(s) | 714 (0.33%) |
|  |  | 4 | 40 (0.86%) | S68 | Traumatic amputation of wrist, hand and fingers | 40 (0.02%) |
| S70-S79 | Injuries to the hip and thigh | 1 | 258 (77.71%) | S70 | Superficial injury of hip and thigh | 167 (0.08%) |
|  |  |  |  | S73 | Dislocation and sprain of joint and ligaments of hip | 50 (0.02%) |
|  |  |  |  | S76 | Injury of muscle, fascia and tendon at hip and thigh level | 41 (0.02%) |
|  |  | 3 | 74 (22.29%) | S72 | Fracture of femur | 74 (0.03%) |
| S80-S89 | Injuries to the knee and lower leg | 1 | 2260 (97.2%) | S80 | Superficial injury of knee and lower leg | 649 (0.3%) |
|  |  |  |  | S82 | Fracture of lower leg, including ankle | 752 (0.35%) |
|  |  |  |  | S83 | Dislocation and sprain of joints and ligaments of knee | 690 (0.32%) |
|  |  |  |  | S89 | Other and unspecified injuries of lower leg | 169 (0.08%) |
|  |  | 2 | 65 (2.8%) | S81 | Open wound of knee and lower leg | 33 (0.02%) |
|  |  |  |  | S86 | Injury of muscle, fascia and tendon at lower leg level | 32 (0.01%) |
| S90-S99 | Injuries to the ankle and foot | 1 | 4376 (100%) | S90 | Superficial injury of ankle, foot and toes | 655 (0.3%) |
|  |  |  |  | S91 | Open wound of ankle, foot and toes | 395 (0.18%) |
|  |  |  |  | S92 | Fracture of foot and toe, except ankle | 1515 (0.7%) |
|  |  |  |  | S93 | Dislocation and sprain of joints and ligaments at ankle, foot and toe level | 1596 (0.74%) |
|  |  |  |  | S99 | Other and unspecified injuries of ankle and foot | 215 (0.1%) |
| T00-T07 | Injuries involving multiple body regions | 1 | 87 (100%) | T00 | Superficial injuries involving multiple body regions | 87 (0.04%) |
| T08-T14 | Injuries to unspecified part of trunk, limb or body region | 1 | 8456 (100%) | T08 | Fracture of spine, level unspecified | 233 (0.11%) |
|  |  |  |  | T09 | Other injuries of spine and trunk, level unspecified | 297 (0.14%) |
|  |  |  |  | T10 | Fracture of upper limb, level unspecified | 18 (0.01%) |
|  |  |  |  | T11 | Other injuries of upper limb, level unspecified | 242 (0.11%) |
|  |  |  |  | T13 | Other injuries of lower limb, level unspecified | 260 (0.12%) |
|  |  |  |  | T14 | Injury of unspecified body region | 7406 (3.44%) |
| T15-T19 | Effects of foreign body entering through natural orifice | 1 | 1253 (84.04%) | T15 | Foreign body on external eye | 38 (0.02%) |
|  |  |  |  | T16 | Foreign body in ear | 297 (0.14%) |
|  |  |  |  | T17 | Foreign body in respiratory tract | 918 (0.43%) |
|  |  | 2 | 238 (15.96%) | T18 | Foreign body in alimentary tract | 238 (0.11%) |
| T20-T32 | Burns and corrosions | 1 | 888 (79%) | T20 | Burn and corrosion of head, face, and neck | 19 (0.01%) |
|  |  |  |  | T21 | Burn and corrosion of trunk | 31 (0.01%) |
|  |  |  |  | T22 | Burn and corrosion of shoulder and upper limb, except wrist and hand | 71 (0.03%) |
|  |  |  |  | T23 | Burn and corrosion of wrist and hand | 128 (0.06%) |
|  |  |  |  | T25 | Burn and corrosion of ankle and foot | 39 (0.02%) |
|  |  |  |  | T30 | Burn and corrosion, body region unspecified | 600 (0.28%) |
|  |  | 2 | 236 (21%) | T24 | Burn and corrosion of lower limb, except ankle and foot | 142 (0.07%) |
|  |  |  |  | T26 | Burn and corrosion confined to eye and adnexa | 16 (0.01%) |
|  |  |  |  | T29 | Burns and corrosions of multiple body regions | 78 (0.04%) |
| T36-T50 | Poisoning by drugs, medicaments and biological substances | 3 | 30 (62.5%) | T50 | Poisoning by, adverse effect of and underdosing of diuretics and other and unspecified drugs, medicaments and biological substances | 30 (0.01%) |
|  |  | 4 | 18 (37.5%) | T45 | Poisoning by, adverse effect of and underdosing of primarily systemic and hematological agents, not elsewhere classified | 18 (0.01%) |
| T51-T65 | Toxic effects of substances chiefly nonmedicinal as to source | 1 | 268 (93.71%) | T59 | Toxic effect of other gases, fumes and vapors | 86 (0.04%) |
|  |  |  |  | T62 | Toxic effect of other noxious substances eaten as food | 46 (0.02%) |
|  |  |  |  | T63 | Toxic effect of contact with venomous animals and plants | 136 (0.06%) |
|  |  | 2 | 18 (6.29%) | T65 | Toxic effect of other and unspecified substances | 18 (0.01%) |
| T66-T78 | Other and unspecified effects of external causes | 1 | 664 (100%) | T70 | Effects of air pressure and water pressure | 26 (0.01%) |
|  |  |  |  | T74 | Adult and child abuse, neglect and other maltreatment, confirmed | 30 (0.01%) |
|  |  |  |  | T78 | Adverse effects, not elsewhere classified | 608 (0.28%) |
| T79-T79 | Certain early complications of trauma | 3 | 327 (100%) | T79 | Certain early complications of trauma, not elsewhere classified | 327 (0.15%) |
| T80-T88 | Complications of surgical and medical care, not elsewhere classified | 1 | 331 (26.4%) | T88 | Other complications of surgical and medical care, not elsewhere classified | 331 (0.15%) |
|  |  | 2 | 528 (42.11%) | T81 | Complications of procedures, not elsewhere classified | 528 (0.25%) |
|  |  | 3  3 | 267 (21.29%)  267 (21.29%) | T83 | Complications of genitourinary prosthetic devices, implants and grafts | 116 (0.05%) |
|  |  |  |  | T85 | Complications of other internal prosthetic devices, implants and grafts | 151 (0.07%) |
|  |  | 4 | 128 (10.21%) | T80 | Complications following infusion, transfusion and therapeutic injection | 20 (0.01%) |
|  |  |  |  | T82 | Complications of cardiac and vascular prosthetic devices, implants and grafts | 108 (0.05%) |
| V01-V99;W00-W99;X00-X59 | Accidents | 1 | 996 (82.45%) | V98 | Other specified transport accidents | 528 (0.25%) |
|  |  |  |  | W19 | Unspecified fall | 468 (0.22%) |
|  |  | 2 | 212 (17.55%) | W54 | Contact with dog | 212 (0.1%) |
| X60-X84 | Intentional self-harm | 2 | 20 (100%) | X84 | Intentional self-harm by unspecified means | 20 (0.01%) |
| X85-X99; Y01-Y09 | Assault | 1 | 771 (100%) | Y09 | Assault by unspecified means | 771 (0.36%) |
| Z00-Z99 | Factors influencing health status and contact with health services | 1 | 446 (89.56%) | Z02 | Encounter for administrative examination | 61 (0.03%) |
|  |  |  |  | Z03 | Encounter for medical observation for suspected diseases and conditions ruled out | 85 (0.04%) |
|  |  |  |  | Z20 | Contact with and (suspected) exposure to communicable diseases | 24 (0.01%) |
|  |  |  |  | Z21 | Asymptomatic human immunodeficiency virus [HIV] infection status | 21 (0.01%) |
|  |  |  |  | Z24 | Need for immunization against certain single bacterial diseases | 38 (0.02%) |
|  |  |  |  | Z33 | Pregnant state | 60 (0.03%) |
|  |  |  |  | Z71 | Persons encountering health services for other counseling and medical advice, not elsewhere classified | 51 (0.02%) |
|  |  |  |  | Z76 | Persons encountering health services in other circumstances | 22 (0.01%) |
|  |  |  |  | Z84 | Family history of other conditions | 32 (0.01%) |
|  |  |  |  | Z86 | Personal history of certain other diseases | 19 (0.01%) |
|  |  |  |  | Z92 | Personal history of medical treatment | 33 (0.02%) |
|  |  | 3 | 29 (5.82%) | Z89 | Acquired absence of limb | 29 (0.01%) |
|  |  | 4 | 23 (4.62%) | Z94 | Transplanted organ and tissue status | 23 (0.01%) |

**Table S6.** Excluded ICD-codes.

| **ICD-10 Code Prefix** | **ICD-10 Code Description** | **Presentation Count** |
| --- | --- | --- |
| C02 | Malignant neoplasm of other and unspecified parts of tongue | 14 |
| C90 | Multiple myeloma and malignant plasma cell neoplasms | 14 |
| D66 | Hereditary factor VIII deficiency | 14 |
| E78 | Disorders of lipoprotein metabolism and other lipidemias | 14 |
| I62 | Other and unspecified nontraumatic intracranial hemorrhage | 14 |
| J00 | Acute nasopharyngitis [common cold] | 14 |
| J12 | Viral pneumonia, not elsewhere classified | 14 |
| J84 | Other interstitial pulmonary diseases | 14 |
| L26 | Exfoliative dermatitis | 14 |
| S19 | Other specified and unspecified injuries of neck | 14 |
| T67 | Effects of heat and light | 14 |
| T84 | Complications of internal orthopedic prosthetic devices, implants and grafts | 14 |
| B24 | Unspecified human immunodeficiency virus [HIV] disease | 13 |
| D43 | Neoplasm of uncertain behavior of brain and central nervous system | 13 |
| G24 | Dystonia | 13 |
| H68 | Eustachian salpingitis and obstruction | 13 |
| H73 | Other disorders of tympanic membrane | 13 |
| I60 | Nontraumatic subarachnoid hemorrhage | 13 |
| J05 | Acute obstructive laryngitis [croup] and epiglottitis | 13 |
| K27 | Peptic ulcer, site unspecified | 13 |
| K51 | Ulcerative colitis | 13 |
| L85 | Other epidermal thickening | 13 |
| L90 | Atrophic disorders of skin | 13 |
| M31 | Other necrotizing vasculopathies | 13 |
| R12 | Heartburn | 13 |
| T19 | Foreign body in genitourinary tract | 13 |
| C24 | Malignant neoplasm of other and unspecified parts of biliary tract | 12 |
| C56 | Malignant neoplasm of ovary | 12 |
| D22 | Melanocytic nevi | 12 |
| D68 | Other coagulation defects | 12 |
| K70 | Alcoholic liver disease | 12 |
| L93 | Lupus erythematosus | 12 |
| N05 | Unspecified nephritic syndrome | 12 |
| N28 | Other disorders of kidney and ureter, not elsewhere classified | 12 |
| Q89 | Other congenital malformations, not elsewhere classified | 12 |
| R47 | Speech disturbances, not elsewhere classified | 12 |
| R82 | Other and unspecified abnormal findings in urine | 12 |
| S79 | Other and unspecified injuries of hip and thigh | 12 |
| Z91 | Personal risk factors, not elsewhere classified | 12 |
| B17 | Other acute viral hepatitis | 11 |
| D24 | Benign neoplasm of breast | 11 |
| F42 | Obsessive-compulsive disorder | 11 |
| G61 | Inflammatory polyneuropathy | 11 |
| H70 | Mastoiditis and related conditions | 11 |
| O03 | Spontaneous abortion | 11 |
| T01 | Open wounds involving multiple body regions | 11 |
| T12 | Fracture of lower limb, level unspecified | 11 |
| T87 | Complications peculiar to reattachment and amputation | 11 |
| Z64 | Problems related to certain psychosocial circumstances | 11 |
| A52 | Late syphilis | 10 |
| B05 | Measles | 10 |
| K00 | Disorders of tooth development and eruption | 10 |
| L65 | Other nonscarring hair loss | 10 |
| R74 | Abnormal serum enzyme levels | 10 |
| S21 | Open wound of thorax | 10 |
| S23 | Dislocation and sprain of joints and ligaments of thorax | 10 |
| S97 | Crushing injury of ankle and foot | 10 |
| Z96 | Presence of other functional implants | 10 |
| B16 | Acute hepatitis B | 9 |
| C67 | Malignant neoplasm of bladder | 9 |
| D32 | Benign neoplasm of meninges | 9 |
| E10 | Type 1 diabetes mellitus | 9 |
| F25 | Schizoaffective disorders | 9 |
| F39 | Unspecified mood [affective] disorder | 9 |
| F69 | Unspecified disorder of adult personality and behavior | 9 |
| F99 | Mental disorder, not otherwise specified | 9 |
| G55 | Nerve root and plexus compressions in diseases classified elsewhere | 9 |
| G57 | Mononeuropathies of lower limb | 9 |
| G58 | Other mononeuropathies | 9 |
| J69 | Pneumonitis due to solids and liquids | 9 |
| K31 | Other diseases of stomach and duodenum | 9 |
| M34 | Systemic sclerosis [scleroderma] | 9 |
| S54 | Injury of nerves at forearm level | 9 |
| T75 | Other and unspecified effects of other external causes | 9 |
| C32 | Malignant neoplasm of larynx | 8 |
| C44 | Other and unspecified malignant neoplasm of skin | 8 |
| D56 | Thalassemia | 8 |
| E88 | Other and unspecified metabolic disorders | 8 |
| F44 | Dissociative and conversion disorders | 8 |
| H71 | Cholesteatoma of middle ear | 8 |
| I63 | Cerebral infarction | 8 |
| K20 | Esophagitis | 8 |
| M33 | Dermatopolymyositis | 8 |
| M87 | Osteonecrosis | 8 |
| N31 | Neuromuscular dysfunction of bladder, not elsewhere classified | 8 |
| Q27 | Other congenital malformations of peripheral vascular system | 8 |
| S12 | Fracture of cervical vertebra and other parts of neck | 8 |
| T39 | Poisoning by, adverse effect of and underdosing of nonopioid analgesics, antipyretics and antirheumatics | 8 |
| A04 | Other bacterial intestinal infections | 7 |
| C19 | Malignant neoplasm of rectosigmoid junction | 7 |
| C23 | Malignant neoplasm of gallbladder | 7 |
| C54 | Malignant neoplasm of corpus uteri | 7 |
| C73 | Malignant neoplasm of thyroid gland | 7 |
| D27 | Benign neoplasm of ovary | 7 |
| I51 | Complications and ill-defined descriptions of heart disease | 7 |
| I99 | Other and unspecified disorders of circulatory system | 7 |
| J95 | Intraoperative and postprocedural complications and disorders of respiratory system, not elsewhere classified | 7 |
| L42 | Pityriasis rosea | 7 |
| M02 | Postinfective and reactive arthropathies | 7 |
| N60 | Benign mammary dysplasia | 7 |
| O02 | Other abnormal products of conception | 7 |
| R15 | Fecal incontinence | 7 |
| Z72 | Problems related to lifestyle | 7 |
| Z87 | Personal history of other diseases and conditions | 7 |
| C05 | Malignant neoplasm of palate | 6 |
| C53 | Malignant neoplasm of cervix uteri | 6 |
| D11 | Benign neoplasm of major salivary glands | 6 |
| D39 | Neoplasm of uncertain behavior of female genital organs | 6 |
| D44 | Neoplasm of uncertain behavior of endocrine glands | 6 |
| E07 | Other disorders of thyroid | 6 |
| F18 | Inhalant related disorders | 6 |
| G60 | Hereditary and idiopathic neuropathy | 6 |
| H51 | Other disorders of binocular movement | 6 |
| I26 | Pulmonary embolism | 6 |
| I72 | Other aneurysm | 6 |
| I97 | Intraoperative and postprocedural complications and disorders of circulatory system, not elsewhere classified | 6 |
| J41 | Simple and mucopurulent chronic bronchitis | 6 |
| J85 | Abscess of lung and mediastinum | 6 |
| K86 | Other diseases of pancreas | 6 |
| N70 | Salpingitis and oophoritis | 6 |
| N84 | Polyp of female genital tract | 6 |
| S04 | Injury of cranial nerve | 6 |
| S14 | Injury of nerves and spinal cord at neck level | 6 |
| S64 | Injury of nerves at wrist and hand level | 6 |
| T42 | Poisoning by, adverse effect of and underdosing of antiepileptic, sedative- hypnotic and antiparkinsonism drugs | 6 |
| Z60 | Problems related to social environment | 6 |
| A01 | Typhoid and paratyphoid fevers | 5 |
| A54 | Gonococcal infection | 5 |
| A92 | Other mosquito-borne viral fevers | 5 |
| C09 | Malignant neoplasm of tonsil | 5 |
| C41 | Malignant neoplasm of bone and articular cartilage of other and unspecified sites | 5 |
| C43 | Malignant melanoma of skin | 5 |
| C71 | Malignant neoplasm of brain | 5 |
| D10 | Benign neoplasm of mouth and pharynx | 5 |
| D45 | Polycythemia vera | 5 |
| D46 | Myelodysplastic syndromes | 5 |
| D47 | Other neoplasms of uncertain behavior of lymphoid, hematopoietic and related tissue | 5 |
| D59 | Acquired hemolytic anemia | 5 |
| E28 | Ovarian dysfunction | 5 |
| E63 | Other nutritional deficiencies | 5 |
| E80 | Disorders of porphyrin and bilirubin metabolism | 5 |
| G30 | Alzheimer's disease | 5 |
| I31 | Other diseases of pericardium | 5 |
| I45 | Other conduction disorders | 5 |
| I71 | Aortic aneurysm and dissection | 5 |
| I74 | Arterial embolism and thrombosis | 5 |
| K25 | Gastric ulcer | 5 |
| K26 | Duodenal ulcer | 5 |
| L10 | Pemphigus | 5 |
| L13 | Other bullous disorders | 5 |
| M18 | Osteoarthritis of first carpometacarpal joint | 5 |
| R03 | Abnormal blood-pressure reading, without diagnosis | 5 |
| R27 | Other lack of coordination | 5 |
| S37 | Injury of urinary and pelvic organs | 5 |
| S41 | Open wound of shoulder and upper arm | 5 |
| T07 | Unspecified multiple injuries | 5 |
| Z48 | Encounter for other postprocedural aftercare | 5 |
| Z75 | Problems related to medical facilities and other health care | 5 |
| Z93 | Artificial opening status | 5 |
| A53 | Other and unspecified syphilis | 4 |
| A87 | Viral meningitis | 4 |
| B33 | Other viral diseases, not elsewhere classified | 4 |
| B83 | Other helminthiases | 4 |
| B85 | Pediculosis and phthiriasis | 4 |
| C04 | Malignant neoplasm of floor of mouth | 4 |
| C49 | Malignant neoplasm of other connective and soft tissue | 4 |
| C83 | Non-follicular lymphoma | 4 |
| C96 | Other and unspecified malignant neoplasms of lymphoid, hematopoietic and related tissue | 4 |
| D12 | Benign neoplasm of colon, rectum, anus and anal canal | 4 |
| D16 | Benign neoplasm of bone and articular cartilage | 4 |
| D21 | Other benign neoplasms of connective and other soft tissue | 4 |
| D33 | Benign neoplasm of brain and other parts of central nervous system | 4 |
| D40 | Neoplasm of uncertain behavior of male genital organs | 4 |
| E24 | Cushing's syndrome | 4 |
| G72 | Other and unspecified myopathies | 4 |
| G98 | Other disorders of nervous system not elsewhere classified | 4 |
| H21 | Other disorders of iris and ciliary body | 4 |
| H27 | Other disorders of lens | 4 |
| H74 | Other disorders of middle ear mastoid | 4 |
| I34 | Nonrheumatic mitral valve disorders | 4 |
| I42 | Cardiomyopathy | 4 |
| I44 | Atrioventricular and left bundle-branch block | 4 |
| J38 | Diseases of vocal cords and larynx, not elsewhere classified | 4 |
| K72 | Hepatic failure, not elsewhere classified | 4 |
| K82 | Other diseases of gallbladder | 4 |
| N36 | Other disorders of urethra | 4 |
| O91 | Infections of breast associated with pregnancy, the puerperium and lactation | 4 |
| P02 | Newborn affected by complications of placenta, cord and membranes | 4 |
| S71 | Open wound of hip and thigh | 4 |
| T35 | Frostbite involving multiple body regions and unspecified frostbite | 4 |
| T61 | Toxic effect of noxious substances eaten as seafood | 4 |
| Y07 | Perpetrator of assault, maltreatment and neglect | 4 |
| Z97 | Presence of other devices | 4 |
| A19 | Miliary tuberculosis | 3 |
| A38 | Scarlet fever | 3 |
| A51 | Early syphilis | 3 |
| A57 | Chancroid | 3 |
| A68 | Relapsing fevers | 3 |
| A91 | Dengue hemorrhagic fever | 3 |
| B19 | Unspecified viral hepatitis | 3 |
| C55 | Malignant neoplasm of uterus, part unspecified | 3 |
| D23 | Other benign neoplasms of skin | 3 |
| D36 | Benign neoplasm of other and unspecified sites | 3 |
| D41 | Neoplasm of uncertain behavior of urinary organs | 3 |
| D50 | Iron deficiency anemia | 3 |
| F30 | Manic episode | 3 |
| F34 | Persistent mood [affective] disorders | 3 |
| F40 | Phobic anxiety disorders | 3 |
| F51 | Sleep disorders not due to a substance or known physiological condition | 3 |
| F68 | Other disorders of adult personality and behavior | 3 |
| G04 | Encephalitis, myelitis and encephalomyelitis | 3 |
| G11 | Hereditary ataxia | 3 |
| G36 | Other acute disseminated demyelination | 3 |
| G37 | Other demyelinating diseases of central nervous system | 3 |
| G46 | Vascular syndromes of brain in cerebrovascular diseases | 3 |
| G71 | Primary disorders of muscles | 3 |
| H80 | Otosclerosis | 3 |
| I33 | Acute and subacute endocarditis | 3 |
| I38 | Endocarditis, valve unspecified | 3 |
| I61 | Nontraumatic intracerebral hemorrhage | 3 |
| I78 | Diseases of capillaries | 3 |
| J81 | Pulmonary edema | 3 |
| K03 | Other diseases of hard tissues of teeth | 3 |
| K41 | Femoral hernia | 3 |
| K55 | Vascular disorders of intestine | 3 |
| K91 | Intraoperative and postprocedural complications and disorders of digestive system, not elsewhere classified | 3 |
| L56 | Other acute skin changes due to ultraviolet radiation | 3 |
| L57 | Skin changes due to chronic exposure to nonionizing radiation | 3 |
| L63 | Alopecia areata | 3 |
| L92 | Granulomatous disorders of skin and subcutaneous tissue | 3 |
| L95 | Vasculitis limited to skin, not elsewhere classified | 3 |
| M40 | Kyphosis and lordosis | 3 |
| M50 | Cervical disc disorders | 3 |
| M81 | Osteoporosis without current pathological fracture | 3 |
| N35 | Urethral stricture | 3 |
| N46 | Male infertility | 3 |
| N62 | Hypertrophy of breast | 3 |
| N71 | Inflammatory disease of uterus, except cervix | 3 |
| N88 | Other noninflammatory disorders of cervix uteri | 3 |
| O85 | Puerperal sepsis | 3 |
| Q66 | Congenital deformities of feet | 3 |
| Q83 | Congenital malformations of breast | 3 |
| R34 | Anuria and oliguria | 3 |
| R72 | Abnormality of white blood cells, not elsewhere classified | 3 |
| R77 | Other abnormalities of plasma proteins | 3 |
| R85 | Abnormal findings in specimens from digestive organs and abdominal cavity | 3 |
| S27 | Injury of other and unspecified intrathoracic organs | 3 |
| T86 | Complications of transplanted organs and tissue | 3 |
| U04 | Severe acute respiratory syndrome [SARS] | 3 |
| Z59 | Problems related to housing and economic circumstances | 3 |
| Z95 | Presence of cardiac and vascular implants and grafts | 3 |
| A31 | Infection due to other mycobacteria | 2 |
| A39 | Meningococcal infection | 2 |
| A59 | Trichomoniasis | 2 |
| A74 | Other diseases caused by chlamydiae | 2 |
| B15 | Acute hepatitis A | 2 |
| B23 | Human immunodeficiency virus [HIV] disease resulting in other conditions | 2 |
| B25 | Cytomegaloviral disease | 2 |
| B54 | Unspecified malaria | 2 |
| B59 | Pneumocystosis | 2 |
| B80 | Enterobiasis | 2 |
| B96 | Other bacterial agents as the cause of diseases classified elsewhere | 2 |
| C01 | Malignant neoplasm of base of tongue | 2 |
| C13 | Malignant neoplasm of hypopharynx | 2 |
| C30 | Malignant neoplasm of nasal cavity and middle ear | 2 |
| C37 | Malignant neoplasm of thymus | 2 |
| C60 | Malignant neoplasm of penis | 2 |
| C66 | Malignant neoplasm of ureter | 2 |
| C77 | Secondary and unspecified malignant neoplasm of lymph nodes | 2 |
| C84 | Mature T/NK-cell lymphomas | 2 |
| D01 | Carcinoma in situ of other and unspecified digestive organs | 2 |
| D29 | Benign neoplasm of male genital organs | 2 |
| D35 | Benign neoplasm of other and unspecified endocrine glands | 2 |
| D58 | Other hereditary hemolytic anemias | 2 |
| D67 | Hereditary factor IX deficiency | 2 |
| D89 | Other disorders involving the immune mechanism, not elsewhere classified | 2 |
| E22 | Hyperfunction of pituitary gland | 2 |
| E23 | Hypofunction and other disorders of the pituitary gland | 2 |
| E27 | Other disorders of adrenal gland | 2 |
| E46 | Unspecified protein-calorie malnutrition | 2 |
| E67 | Other hyperalimentation | 2 |
| E79 | Disorders of purine and pyrimidine metabolism | 2 |
| F60 | Specific personality disorders | 2 |
| F84 | Pervasive developmental disorders | 2 |
| F90 | Attention-deficit hyperactivity disorders | 2 |
| F95 | Tic disorder | 2 |
| G06 | Intracranial and intraspinal abscess and granuloma | 2 |
| G12 | Spinal muscular atrophy and related syndromes | 2 |
| G21 | Secondary parkinsonism | 2 |
| G64 | Other disorders of peripheral nervous system | 2 |
| G80 | Cerebral palsy | 2 |
| G96 | Other disorders of central nervous system | 2 |
| H55 | Nystagmus and other irregular eye movements | 2 |
| I12 | Hypertensive chronic kidney disease | 2 |
| I35 | Nonrheumatic aortic valve disorders | 2 |
| I46 | Cardiac arrest | 2 |
| I65 | Occlusion and stenosis of precerebral arteries, not resulting in cerebral infarction | 2 |
| J37 | Chronic laryngitis and laryngotracheitis | 2 |
| J42 | Unspecified chronic bronchitis | 2 |
| J43 | Emphysema | 2 |
| K44 | Diaphragmatic hernia | 2 |
| K71 | Toxic liver disease | 2 |
| L80 | Vitiligo | 2 |
| M95 | Other acquired deformities of musculoskeletal system and connective tissue | 2 |
| N03 | Chronic nephritic syndrome | 2 |
| N15 | Other renal tubulo-interstitial diseases | 2 |
| N85 | Other noninflammatory disorders of uterus, except cervix | 2 |
| N87 | Dysplasia of cervix uteri | 2 |
| O04 | Complications following (induced) termination of pregnancy | 2 |
| O46 | Antepartum hemorrhage, not elsewhere classified | 2 |
| O73 | Retained placenta and membranes, without hemorrhage | 2 |
| O90 | Complications of the puerperium, not elsewhere classified | 2 |
| Q53 | Undescended and ectopic testicle | 2 |
| Q67 | Congenital musculoskeletal deformities of head, face, spine and chest | 2 |
| R64 | Cachexia | 2 |
| R76 | Other abnormal immunological findings in serum | 2 |
| R86 | Abnormal findings in specimens from male genital organs | 2 |
| T31 | Burns classified according to extent of body surface involved | 2 |
| T60 | Toxic effect of pesticides | 2 |
| T66 | Radiation sickness, unspecified | 2 |
| Z56 | Problems related to employment and unemployment | 2 |
| Z88 | Allergy status to drugs, medicaments and biological substances | 2 |
| Z99 | Dependence on enabling machines and devices, not elsewhere classified | 2 |
| A02 | Other salmonella infections | 1 |
| A05 | Other bacterial foodborne intoxications, not elsewhere classified | 1 |
| A07 | Other protozoal intestinal diseases | 1 |
| A17 | Tuberculosis of nervous system | 1 |
| A18 | Tuberculosis of other organs | 1 |
| A48 | Other bacterial diseases, not elsewhere classified | 1 |
| A58 | Granuloma inguinale | 1 |
| A66 | Yaws | 1 |
| A69 | Other spirochetal infections | 1 |
| A79 | Other rickettsioses | 1 |
| A82 | Rabies | 1 |
| A99 | Unspecified viral hemorrhagic fever | 1 |
| B06 | Rubella [German measles] | 1 |
| B45 | Cryptococcosis | 1 |
| B48 | Other mycoses, not elsewhere classified | 1 |
| B77 | Ascariasis | 1 |
| B89 | Unspecified parasitic disease | 1 |
| B97 | Viral agents as the cause of diseases classified elsewhere | 1 |
| C06 | Malignant neoplasm of other and unspecified parts of mouth | 1 |
| C07 | Malignant neoplasm of parotid gland | 1 |
| C14 | Malignant neoplasm of other and ill-defined sites in the lip, oral cavity and pharynx | 1 |
| C21 | Malignant neoplasm of anus and anal canal | 1 |
| C31 | Malignant neoplasm of accessory sinuses | 1 |
| C38 | Malignant neoplasm of heart, mediastinum and pleura | 1 |
| C40 | Malignant neoplasm of bone and articular cartilage of limbs | 1 |
| C45 | Mesothelioma | 1 |
| C47 | Malignant neoplasm of peripheral nerves and autonomic nervous system | 1 |
| C68 | Malignant neoplasm of other and unspecified urinary organs | 1 |
| C75 | Malignant neoplasm of other endocrine glands and related structures | 1 |
| C81 | Hodgkin lymphoma | 1 |
| D03 | Melanoma in situ | 1 |
| D05 | Carcinoma in situ of breast | 1 |
| D13 | Benign neoplasm of other and ill-defined parts of digestive system | 1 |
| D30 | Benign neoplasm of urinary organs | 1 |
| D51 | Vitamin B12 deficiency anemia | 1 |
| D73 | Diseases of spleen | 1 |
| D84 | Other immunodeficiencies | 1 |
| D86 | Sarcoidosis | 1 |
| E13 | Other specified diabetes mellitus | 1 |
| E21 | Hyperparathyroidism and other disorders of parathyroid gland | 1 |
| E29 | Testicular dysfunction | 1 |
| E66 | Overweight and obesity | 1 |
| E71 | Disorders of branched-chain amino-acid metabolism and fatty-acid metabolism | 1 |
| E72 | Other disorders of amino-acid metabolism | 1 |
| E85 | Amyloidosis | 1 |
| E89 | Postprocedural endocrine and metabolic complications and disorders, not elsewhere classified | 1 |
| F00 | Dementia in Alzheimer disease | 1 |
| F11 | Opioid related disorders | 1 |
| F48 | Other nonpsychotic mental disorders | 1 |
| F63 | Impulse disorders | 1 |
| F64 | Gender identity disorders | 1 |
| F79 | Unspecified intellectual disabilities | 1 |
| F91 | Conduct disorders | 1 |
| G23 | Other degenerative diseases of basal ganglia | 1 |
| G63 | Polyneuropathy in diseases classified elsewhere | 1 |
| G82 | Paraplegia (paraparesis) and quadriplegia (quadriparesis) | 1 |
| G91 | Hydrocephalus | 1 |
| H30 | Chorioretinal inflammation | 1 |
| H31 | Other disorders of choroid | 1 |
| H50 | Other strabismus | 1 |
| I07 | Rheumatic tricuspid valve diseases | 1 |
| I09 | Other rheumatic heart diseases | 1 |
| I15 | Secondary hypertension | 1 |
| I27 | Other pulmonary heart diseases | 1 |
| I30 | Acute pericarditis | 1 |
| I37 | Nonrheumatic pulmonary valve disorders | 1 |
| I70 | Atherosclerosis | 1 |
| J09 | Influenza due to certain identified influenza viruses | 1 |
| J21 | Acute bronchiolitis | 1 |
| J67 | Hypersensitivity pneumonitis due to organic dust | 1 |
| J86 | Pyothorax | 1 |
| J94 | Other pleural conditions | 1 |
| K09 | Cysts of oral region, not elsewhere classified | 1 |
| K90 | Intestinal malabsorption | 1 |
| L00 | Staphylococcal scalded skin syndrome | 1 |
| L43 | Lichen planus | 1 |
| L71 | Rosacea | 1 |
| L88 | Pyoderma gangrenosum | 1 |
| M08 | Juvenile arthritis | 1 |
| M12 | Other and unspecified arthropathy | 1 |
| M15 | Polyosteoarthritis | 1 |
| M30 | Polyarteritis nodosa and related conditions | 1 |
| M80 | Osteoporosis with current pathological fracture | 1 |
| M85 | Other disorders of bone density and structure | 1 |
| M96 | Intraoperative and postprocedural complications and disorders of musculoskeletal system, not elsewhere classified | 1 |
| M99 | Biomechanical lesions, not elsewhere classified | 1 |
| N00 | Acute nephritic syndrome | 1 |
| N01 | Rapidly progressive nephritic syndrome | 1 |
| N02 | Recurrent and persistent hematuria | 1 |
| N11 | Chronic tubulo-interstitial nephritis | 1 |
| N16 | Renal tubulo-interstitial disorders in diseases classified elsewhere | 1 |
| N25 | Disorders resulting from impaired renal tubular function | 1 |
| N42 | Other and unspecified disorders of prostate | 1 |
| N72 | Inflammatory disease of cervix uteri | 1 |
| N82 | Fistulae involving female genital tract | 1 |
| O08 | Complications following ectopic and molar pregnancy | 1 |
| O13 | Gestational [pregnancy-induced] hypertension without significant proteinuria | 1 |
| O23 | Infections of genitourinary tract in pregnancy | 1 |
| O71 | Other obstetric trauma | 1 |
| O72 | Postpartum hemorrhage | 1 |
| O86 | Other puerperal infections | 1 |
| Q07 | Other congenital malformations of nervous system | 1 |
| Q28 | Other congenital malformations of circulatory system | 1 |
| Q33 | Congenital malformations of lung | 1 |
| Q44 | Congenital malformations of gallbladder, bile ducts and liver | 1 |
| Q55 | Other congenital malformations of male genital organs | 1 |
| Q64 | Other congenital malformations of urinary system | 1 |
| Q65 | Congenital deformities of hip | 1 |
| Q69 | Polydactyly | 1 |
| Q75 | Other congenital malformations of skull and face bones | 1 |
| Q76 | Congenital malformations of spine and bony thorax | 1 |
| Q78 | Other osteochondrodysplasias | 1 |
| Q79 | Congenital malformations of musculoskeletal system, not elsewhere classified | 1 |
| Q80 | Congenital ichthyosis | 1 |
| Q81 | Epidermolysis bullosa | 1 |
| Q85 | Phakomatoses, not elsewhere classified | 1 |
| R57 | Shock, not elsewhere classified | 1 |
| R70 | Elevated erythrocyte sedimentation rate and abnormality of plasma viscosity | 1 |
| R81 | Glycosuria | 1 |
| R83 | Abnormal findings in cerebrospinal fluid | 1 |
| R87 | Abnormal findings in specimens from female genital organs | 1 |
| R89 | Abnormal findings in specimens from other organs, systems and tissues | 1 |
| R90 | Abnormal findings on diagnostic imaging of central nervous system | 1 |
| R93 | Abnormal findings on diagnostic imaging of other body structures | 1 |
| S44 | Injury of nerves at shoulder and upper arm level | 1 |
| S96 | Injury of muscle and tendon at ankle and foot level | 1 |
| T43 | Poisoning by, adverse effect of and underdosing of psychotropic drugs, not elsewhere classified | 1 |
| T51 | Toxic effect of alcohol | 1 |
| T54 | Toxic effect of corrosive substances | 1 |
| T69 | Other effects of reduced temperature | 1 |
| T73 | Effects of other deprivation | 1 |
| T95 | Sequelae of burns, corrosions and frostbite | 1 |
| V88 | Nontraffic accident of specified type but victim's mode of transport unknown | 1 |
| W23 | Caught, crushed, jammed or pinched in or between objects | 1 |
| W91 | Exposure to unspecified type of radiation | 1 |
| Y20 | Hanging, strangulation and suffocation, undetermined intent | 1 |
| Z30 | Encounter for contraceptive management | 1 |
| Z35 | Supervision of high-risk pregnancy | 1 |
| Z47 | Orthopedic aftercare | 1 |
| Z53 | Persons encountering health services for specific procedures and treatment, not carried out | 1 |
| Z73 | Problems related to life management difficulty | 1 |
| Z82 | Family history of certain disabilities and chronic diseases (leading to disablement) | 1 |
| Z85 | Personal history of malignant neoplasm | 1 |
| Z98 | Other postprocedural states | 1 |

**Table S7.** ED presentation characteristics, including ICD-10 codes with at least 10 presentations.

| Characteristics | Overall | Cluster 1 | Cluster 2 | Cluster 3 | Cluster 4 |
| --- | --- | --- | --- | --- | --- |
| Size, N (%) | 216183 (100) | 131851 (61.0) | 44473 (20.6) | 27929 (12.9) | 11930 (5.5) |
| ICD-10 code prefixes, N | 567 | 289 | 114 | 73 | 91 |
| Admission rate, % (SD) | 17.4 ± 0.4 | 4.7 ± 0.2 | 19.5 ± 0.4 | 47.7 ± 0.5 | 78.1 ± 0.4 |
| Sex, N (%) |  |  |  |  |  |
| Female | 100791 (46.6) | 60025 (45.5) | 22121 (49.7) | 13146 (47.1) | 5499 (46.1) |
| Male | 115392 (53.4) | 71826 (54.5) | 22352 (50.3) | 14783 (52.9) | 6431 (53.9) |
| Age, mean (SD) | 46.2 (19.4) | 44.8 (19.2) | 45.4 (19.4) | 49.2 (19.2) | 57.0 (18.2) |
| Ethnicity |  |  |  |  |  |
| Chinese, N (%) | 126191 (58.4) | 74940 (56.8) | 26351 (59.2) | 16866 (60.4) | 8034 (67.3) |
| Indian, N (%) | 33577 (15.5) | 21506 (16.3) | 6449 (14.5) | 4273 (15.3) | 1349 (11.3) |
| Malay, N (%) | 26396 (12.2) | 16033 (12.2) | 5604 (12.6) | 3379 (12.1) | 1380 (11.6) |
| Others, N (%) | 30019 (13.9) | 19372 (14.7) | 6069 (13.6) | 3411 (12.2) | 1167 (9.8) |
| Charlson Comorbidity Index |  |  |  |  |  |
| None, N (%) | 176988 (81.9) | 115966 (88.0) | 36281 (81.6) | 19127 (68.5) | 5614 (47.1) |
| Mild, N (%) | 23757 (11.0) | 10733 (8.1) | 5038 (11.3) | 5065 (18.1) | 2921 (24.5) |
| Moderate, N (%) | 8146 (3.8) | 3098 (2.4) | 1688 (3.8) | 1822 (6.5) | 1538 (12.9) |
| Severe, N (%) | 7292 (3.4) | 2054 (1.6) | 1466 (3.3) | 1915 (6.9) | 1857 (15.6) |
| Systolic Blood Pressure |  |  |  |  |  |
| Normal, N (%) | 126316 (58.4) | 79853 (60.6) | 25638 (57.6) | 15030 (53.8) | 5795 (48.6) |
| Hypotension, N (%) | 300 (0.1) | 164 (0.1) | 59 (0.1) | 57 (0.2) | 20 (0.2) |
| Hypertension, N (%) | 89567 (41.4) | 51834 (39.3) | 18776 (42.2) | 12842 (46.0) | 6115 (51.3) |
| Pulse Rate |  |  |  |  |  |
| Normal, N (%) | 150825 (69.8) | 96659 (73.3) | 30646 (68.9) | 16537 (59.2) | 6983 (58.5) |
| Bradycardia, N (%) | 7683 (3.5) | 4870 (3.7) | 1522 (3.4) | 870 (3.1) | 421 (3.5) |
| Tachycardia, N (%) | 57675 (26.7) | 30322 (23) | 12305 (27.7) | 10522 (37.7) | 4526 (37.9) |
| Temperature |  |  |  |  |  |
| Normal, N (%) | 194233 (89.8) | 122043 (92.6) | 40511 (91.1) | 22245 (79.7) | 9434 (79.1) |
| Hypothermia, N (%) | 4388 (2.0) | 2604 (2.0) | 1034 (2.3) | 479 (1.7) | 271 (2.3) |
| Hyperthermia, N (%) | 17562 (8.1) | 7204 (5.5) | 2928 (6.6) | 5205 (18.6) | 2225 (18.6) |
| N of previous ED visits, mean (SD) | 0.6 ± 1.9 | 0.7 ± 2 | 0.6 ± 1.9 | 0.5 ± 1.4 | 0.6 ± 1.3 |
| N of previous inpatient visits, mean (SD) | 0.1 ± 0.6 | 0.1 ± 0.6 | 0.2 ± 0.7 | 0.2 ± 0.6 | 0.3 ± 0.8 |
| N of recent surgeries, mean (SD) | 0.0 ± 0.3 | 0.0 ± 0.2 | 0.0 ± 0.3 | 0.1 ± 0.4 | 0.1 ± 0.6 |
| N of previous ICU admissions, mean (SD) | 0.0 ± 0.1 | 0.0 ± 0.0 | 0.0 ± 0.1 | 0.0 ± 0.1 | 0.0 ± 0.1 |
| Previous HD admissions, mean (SD) | 0.0 ± 0.1 | 0.0 ± 0.1 | 0.0 ± 0.1 | 0.0 ± 0.2 | 0.0 ± 0.2 |
| Previous ICA admissions, mean (SD) | 0.0 ± 0.1 | 0.0 ± 0.1 | 0.0 ± 0.1 | 0.0 ± 0.1 | 0.0 ± 0.2 |
| Previous ID admissions, mean (SD) | 0.0 ± 0.4 | 0.0 ± 0.3 | 0.0 ± 0.4 | 0.0 ± 0.5 | 0.1 ± 0.7 |

Abbreviations: N, number; SD, standard deviation; ED, emergency department; ICU, intensive care unit; HD, high dependency; ICA, intermediate care area; ID, infectious disease

**Table S8.** Number ICD-10 blocks according to different cumulative thresholds, when including ICD-10 codes with at least 10 presentations.

| Cumulative coverage (%) | ICD-10 blocks (N) |
| --- | --- |
| 100 | 136 |
| 90 | 52 |
| 80 | 33 |
| 70 | 22 |
| 60 | 15 |

*The additional 4 blocks added when ICD-10 codes with at least 10 presentations were utilised for analyses were: B20-B24 Human immunodeficiency virus [HIV] disease; Q80-Q89 Other congenital malformations; N25-N29 Other disorders of kidney and ureter; J80-J84 Other respiratory diseases principally affecting the interstitium.
