# Supplementary material for: Pragmatic Risk Stratification Method to Identify Emergency Department Presentations for Alternative Care Service Pathways: Registry-Based Retrospective Study Over 5 Years
Source: J Med Internet Res. 2025 May 12;27:e73758. doi: 10.2196/73758 (PMC12107196; doi:10.2196/73758)
Supplement: Multimedia Appendix 3 [file jmir_v27i1e73758_app3.pdf]

Transcript with chatbot to refine spelling and grammar

You are to assume a role of a proofreader. Review the following text for spelling and grammar issues. Only provide suggested changes. Do not make any direct changes to the manuscript. I will review the suggestions, and make the final edits myself. Text will be submitted in chunks. There is no need to comment on superscripts.

ChatGPT said:

Understood. Please go ahead and submit the first chunk of text for review.

4o

You said:

The emergency medical system is a critical part of healthcare that should provide accessible and time-sensitive care for acute illnesses and injuries. However, it faces increasing challenges due to the growing demand driven by aging populations and complexity of care1. Suboptimal usage of Pre-hospital Emergency Care (PEC) refers to the activation of the system for conditions that may otherwise be treated at alternative healthcare settings, without the need for urgent transport to the emergency department (ED). Globally, the utilisation of ED services is rising, with non-urgent visits estimated to comprise of 20-40% of all ED attendances2-4. Such non-urgent visits contribute to hospital overcrowding, prolonged waiting times, increased risk of medication errors, and poorer health outcomes5-8. Redirecting low acuity patients to alternative care settings may lead to better allocation of resources within PEC, ensuring that the ED is reserved for urgent cases. This approach reduces system-wide stress and delays, while low-acuity patients can be managed safely in appropriate care settings9-11. An important consideration is determining how to stratify ED patients based on their admission risk to better identify those who might be suitable for alternative care. Diagnosis codes provide a clearer and comprehensive reflection of a patient’s care needs compared to triage information. There is limited concordance between presenting complaints and diagnoses, limiting the use of triage data for classification of non-urgent cases12. Utilising diagnosis codes provides a framework to understand population-level needs that may inform emergency care service delivery. Although the International Classification of Diseases (ICD)-10 coding system provides a detailed classification with over 68,000 unique codes which are organized into chapters and subsections, its high level of granularity poses challenges for practical applications such as admission risk stratification13. Aggregating these codes into meaningful categories that retain predictive relevance while minimising diagnostic oversimplification remains a challenge14. Clustering methods have been applied to various ED cohorts, primarily focusing on demographic and comorbidity data15,16. However, the variables used in these studies to define cluster characteristics were selected based on traits deemed clinically relevant, without a clear or quantitative explanation. Similarly, another study clustered patients based on the co-occurrence of diagnoses, using diagnosis codes to identify patterns of multimorbidity in the ED13. While informative at a systems level, this approach did not incorporate admission risk. There are no studies, to the authors' best knowledge, that cluster ED patients according to admission risk based on diagnosis codes. Therefore, the aim of this study was to identify key patient characteristics associated with admission risk, and to develop a pragmatic risk-based classification to identify ED patient groups potentially suitable for alternative care settings. By consolidating diagnosis codes into clinically meaningful categories, the study sought to overcome

the challenges of granular coding and identify subgroups of patients with lower admission probabilities for better allocation of PEC resources.

ChatGPT said:

Here are the suggested changes for spelling and grammar:

1. **"complexity of care1"** → consider adding **"the"** before **"complexity"** for parallel structure:
   *"...driven by aging populations and **the** complexity of care..."*
2. **"comprise of 20–40%"** → remove **"of"**:
   *"...estimated to comprise **20–40%** of all ED attendances..."*
3. **"low acuity patients"** → hyphenate for clarity:
   "low-acuity patients"
4. **"reduces system-wide stress and delays, while low-acuity patients"** → consider replacing **"while"** with **"and"** to avoid ambiguity:
   *"reduces system-wide stress and delays, **and** low-acuity patients..."*
5. **"to better identify those who might be suitable"** → consider removing **"better"** for conciseness unless you are contrasting with a previous method:
   "...to identify those who might be suitable..."
6. **"Organized into chapters and subsections"** → consider using British spelling if consistency with UK spelling is preferred:
   "Organised into chapters and subsections"
7. **"remains a challenge14"** → add a comma before "remains" if the sentence is long or complex:
   "...while minimising diagnostic oversimplification, remains a challenge..."
8. **"traits deemed clinically relevant"** → consider adding **"as"** for clarity:
   *"...traits deemed **as** clinically relevant..."*
9. **"to the authors' best knowledge"** → more standard phrasing:
   "...to the best of the authors' knowledge..."

Would you like me to proceed with the next chunk?

4o

You said:

Singapore is a dense urban city-state with a population of approximately 6.0 million and a land area of 734 km². Singapore's healthcare system comprises a mix of public and private providers, with the public sector managing the majority of healthcare needs18. While the annual population growth rate of Singapore is approximately 1.1%, ED attendance has risen disproportionately, increasing around 5.6% annually between 2005 and 201619,20. The study hospital is a tertiary care institution and the largest of Singapore’s ten acute care public hospitals, accounting for one-fifth of total acute hospital beds nationwide. The Singapore Health Services (SingHealth) cluster primarily serves the eastern region of Singapore, of which this hospital is part of. The study hospital serves more than a million patients annually, and within this, the ED manages around 130,000 patient visits annually with more than approximately 40,000 of these being admitted as inpatients21.

ChatGPT said:

Here are the suggested changes for spelling and grammar:

1. **"Singapore is a dense urban city-state"** → consider removing either *"dense"* or *"urban"* for conciseness unless you intend to emphasise both distinctly.
2. **"734 km²"** → consider spelling out the unit for formal writing:
   "734 square kilometres"
3. **"Singapore's healthcare system comprises"** → change to British spelling for consistency if UK spelling is preferred:
   "Singapore's healthcare system comprises ***a mix of*** *public and private providers..."* (no change necessary unless you're revising spelling).
4. **"increasing around 5.6% annually"** → revise to:
   "increasing ***by*** *around 5.6% annually..."*
5. **"of which this hospital is part of"** → avoid the double preposition **"of ... of"**:
   "...which this hospital is part of" → *"...**of which this hospital is a part**."*
6. **"with more than approximately 40,000 of these being admitted"** → **"more than"** and **"approximately"** are redundant together. Choose one:
   "...with ***more than 40,000*** *of these being admitted..."* or *"...with* ***approximately 40,000*** *of these being admitted..."*

Let me know when you're ready with the next chunk.

4o

You said:

The study utilised an established ED database which includes de-identified data extracted from the SingHealth Electronic Health Intelligence System (eHints), a comprehensive data integration platform within the SingHealth cluster. This platform consolidates data from multiple hospital systems, including clinical records, ED registrations, admissions, as well as operations and finance data sources. To maintain patient confidentiality, all data were de-identified by masking details of their ED case number, admission case number, and patient identification number. Electronic health records from other public and private hospitals were not available. Data from January 2016 to December 2020 were extracted, during which the Systematized Nomenclature of Medicine Clinical Terms (SNOMED CT) was exclusively used for assigning diagnosis codes. SNOMED CT provides a comprehensive classification that is useful for clinical care and documentation. However, it is a concept model, which represents clinical ideas such as diseases, symptoms, and procedures; and has a polyhierarchical structure, where a single concept can belong to multiple categories, presenting challenges for health services reporting and research22. The ICD-10 system offers a standardized and hierarchical classification framework better suited for such analyses23,24, and the SNOMED CT codes were thus converted to the ICD-10 system with minimal data loss. Prior data from 2008 to 2014 were recorded using ICD-9 codes, and were excluded from the present analyses due to their limited specificity and structural differences compared to ICD-10. The study included ED records for patients of all ages classified as Priority Levels 3 and 4 under the Patient Acuity Category Scale25, representing ambulatory patients requiring relatively 'stable' ED care11. Exclusion criteria were records for Priority

Level 1 and 2 patients, who required critical or urgent care, as well as those with records outside the timeframe of interest (2015-2020).

ChatGPT said:

Here are the suggested changes for spelling and grammar:

1. **"utilised"** → correct as British spelling; no change needed if UK spelling is preferred.
2. **"which includes de-identified data extracted..."** → consider replacing **"which"** with **"that"** for defining clause clarity:
*"an established ED database **that** includes de-identified data..."*
3. **"as well as operations and finance data sources"** → consider rewording for parallel structure:
"...admissions, operations, and finance data sources."
4. **"Electronic health records from other public and private hospitals were not available."** → consider clarifying whether this limitation applies to the dataset or to broader interoperability.
5. **"January 2016 to December 2020 were extracted"** → smoother phrasing:
"Data were extracted for the period from January 2016 to December 2020..."
6. **"and has a polyhierarchical structure, where..."** → revise to:
*"...and has a polyhierarchical structure **in which** a single concept can belong to multiple categories..."*
7. **"challenges for health services reporting and research22"** → add a comma before the citation if ending a clause:
"...presenting challenges for health services reporting and research,**"*
8. **"standardized"** → change to British spelling:
"standardised"
9. **"with minimal data loss."** → consider adding "incurred" for clarity:
*"...converted to the ICD-10 system with minimal data loss **incurred**."*
10. **"Prior data from 2008 to 2014"** → consider specifying "ED data" for clarity:
*"Prior **ED** data from 2008 to 2014..."*
11. **"'stable' ED care11"** → consider removing quotation marks unless "stable" is a direct quote or term needing emphasis.
12. **"records for Priority Level 1 and 2 patients"** → consider:
*"records **of** Priority Level 1 and 2 patients"*

Let me know when you're ready to proceed with the next section.

4o

You said:

This study demonstrated the utility of clustering ICD-10 codes as a novel method for stratifying patient admission outcomes in emergency departments among ambulatory patients. Using

unsupervised learning for data mining, four distinct clusters were identified. A four-cluster solution provided a balance between statistical robustness and clinical interpretability to identify meaningful distinctions in admission risks. Clustering ICD-10 codes revealed a wide range in admission risks, ranging from 4.7% in cluster 1 to 78.0% in cluster 4. Cluster 1 represents low-acuity cases that require minimal intervention or monitoring such as musculoskeletal conditions (e.g., dorsalgia, spondylosis) and skin conditions (e.g., urticaria), as shown by 84.0% of patients in in this cluster not requiring laboratory investigations as well. However, cluster 4 identified higher risk patients with more complex medical conditions, despite their stable and ambulatory classification at triage. Although this cluster consists of a minority of the total sample, its high admission risk underscores the need for a triage process that prioritises sensitivity for the higher admission risk clusters, even at the expense of some false positives. The gradient of risk also shows that admission risk patterns were non-linear in distribution, with cluster 2 increasing to nearly 20% admission risk, and cluster 3 characterised by nearly 50% admission risk. Logistic regression analysis identified key predictors of cluster membership, including CCI, vital sign abnormalities (hyperthermia, tachycardia), and recent surgical history. Patients with moderate and severe comorbidities were significantly more likely to belong to higher-risk clusters, which is consistent with prior studies that emphasise the predictive value of CCI for hospitalisation and poorer outcomes38-40. The study findings also align with other studies that show that vital sign abnormalities, such as hyperthermia and tachycardia, were associated with higher admission risks41. In contrast to other studies which have linked frequent ED visits with higher admission risk42,43, our analysis did not find a strong association between prior ED attendance and being in a higher-risk cluster. This may be due to the inclusion of lower acuity cases in our cohort, where frequent ED utilisation may not necessarily indicate severe or worsening health conditions. 132 unique ICD-10 blocks were identified within the sample, but this number may be too granular to screen for alternative care pathways. However, when coverage was reduced to 90% of patients, the number of ICD-10 blocks decreased to a more manageable 52, and further reduction to 80% coverage reduced it to 33 blocks. At the 80% cut-off, the lowest-risk cluster (Cluster 1) consisted of 27 ICD-10 blocks of any proportion, with 17 blocks representing ≥75% of the cases in this group, accounting for 43.8% of the total sample. These predominantly included conditions such as musculoskeletal/connective tissue disorders and injuries to the head and lower limbs. The large proportion of low-risk patients in Cluster 1, along with identifying patients with high-representation ICD-10 blocks in lower admission risk clusters, offers opportunities to redirect these patients to alternative care settings and efficient resource allocation20,44. The strength of this study lies in identifying categories of diagnoses that can be considered for alternative care settings, within a globally recognised and highly granular classification system. Understanding the distribution of lower-risk conditions enables optimising resource allocation such as establishing urgent care centres and primary care centres to be equipped to manage conditions like musculoskeletal conditions such as arthropathies and dorsopathies, and minor external injuries to the upper and lower limbs, which were predominantly grouped into cluster 1 with the lowest admission risk. It is important to distinguish identifying non-urgent cases from addressing ED overcrowding, which is influenced by more complex factors such as overburdened inpatient facilities, staffing shortages, and aging populations3,45. Rather, the findings suggest that certain conditions could be potentially treated outside the ED, which is intended for rapid, unscheduled care of urgent cases. Managing stable cases in primary care can also foster stronger patient-provider relationships46. This study has several limitations. Firstly, it is a single-centre study conducted at a tertiary academic centre, which may have received patients which are demographically different from the wider population. However, given Singapore's status as a small city-state, these differences may be minimal. Nonetheless, the results should be externally validated in diverse geographical regions for broader applicability. Secondly,

cluster analysis lacks a perfectly objective method for determining the number of clusters. While statistical criteria such as the gap statistic and elbow plot can guide the process, clusters must also have a balance between parsimony and clinical interpretability47. Additionally, admission risk was determined solely based on ICD-10 diagnoses, without accounting for other potential confounders such as patient comorbidities, access to outpatient specialty care, or social determinants of health such as deprivation index and population dependency ratio48. ICD-10 diagnoses may also not reflect precise clinical diagnoses, with conditions such as heart failure being inaccurately coded due to overlap in symptoms and variability in documentation practices49. Furthermore, due the lack of access to unstructured data, such as discharge summaries or case notes, it was not possible to further investigate the decision-making process in assigning diagnosis codes, or the decisions behind admitting patients50. Lastly, diagnosis codes were assigned based on their final disposition, suggesting that triaging of non-urgent cases suitable for alternative care needs to be identified earlier in the PEC pathway.

ChatGPT said:

Here are the suggested changes for spelling and grammar:

1. **"in in this cluster"** → remove the repeated word:
   *"...84.0% of patients **in this cluster** not requiring..."*
2. **"not requiring laboratory investigations as well"** → stylistically awkward; consider:
   "...not requiring laboratory investigations either." or *"...also not requiring laboratory investigations."*
3. **"Cluster 1 represents low-acuity cases that require minimal intervention or monitoring such as musculoskeletal conditions..."** → add a comma for clarity:
   "...monitoring,** such as musculoskeletal conditions..."*
4. **"Cluster 4 identified higher risk patients"** → hyphenate **"higher-risk"**:
   *"Cluster 4 identified **higher-risk** patients..."*
5. **"gradient of risk also shows that admission risk patterns were non-linear in distribution"** → rephrase slightly for conciseness:
   "The gradient of risk indicates a non-linear distribution of admission risk patterns..."
6. **"which is consistent with prior studies that emphasise..."** → match UK spelling:
   *"...studies that **emphasise**..."*
7. **"The study findings also align with other studies that show that vital sign abnormalities..."** → avoid repetition of **"that"**:
   *"...align with other studies **showing** that vital sign abnormalities..."*
8. **"such as hyperthermia and tachycardia, were associated"** → no comma needed:
   "...vital sign abnormalities such as hyperthermia and tachycardia were associated..."
9. **"with high-representation ICD-10 blocks in lower admission risk clusters"** → hyphenate **"lower-admission-risk"**:
   *"...blocks in **lower-admission-risk** clusters..."*
10. **"such as establishing urgent care centres and primary care centres to be equipped to manage"** → consider simplifying:
    "...such as equipping urgent care and primary care centres to manage..."

11. **"musculoskeletal conditions such as arthropathies and dorsopathies, and minor external injuries"** → consider removing the first **"such as"** to avoid repetition:
    "...conditions like arthropathies and dorsopathies, and minor external injuries..."
12. **"which were predominantly grouped into cluster 1"** → capitalise Cluster:
    *"...grouped into **Cluster 1**..."*
13. **"patients which are demographically different"** → "which" → **"who"**, and rephrase:
    *"...patients **who may differ demographically** from the wider population."*
14. **"due the lack of access"** → missing preposition:
    *"...**due to** the lack of access..."*
15. **"triaging of non-urgent cases suitable for alternative care needs to be identified earlier"** → rephrase for clarity:
    *"triaging of non-urgent cases suitable for alternative care **must occur earlier** in the PEC pathway."*

Would you like help proofreading the next section or with any other revisions?

4o

You said:

This study demonstrates the utility of clustering ICD-10 codes as a novel approach for stratifying patient admission outcomes in the ED among ambulatory patients. Admission risk patterns were non-linear, and applying thresholds to group ICD-10 blocks based on the proportion of patients they represent may provide a balance by maintaining adequate representation of admission risk groups without losing too much granularity. Future work should focus on accurately identifying these cases earlier in the PEC pathway for possible decanting for alternative care settings.

ChatGPT said:

Here are the suggested changes for spelling and grammar:

1. **"as a novel approach for stratifying patient admission outcomes in the ED among ambulatory patients"** → consider reordering for clarity and conciseness:
   "as a novel approach to stratify admission outcomes among ambulatory ED patients."
2. **"Admission risk patterns were non-linear"** → consider specifying "non-linear in distribution" for consistency with earlier usage:
   "Admission risk patterns were non-linear in distribution..."
3. **"may provide a balance by maintaining adequate representation of admission risk groups without losing too much granularity"** → consider refining for clarity:
   "...may strike a balance between preserving admission risk group representation and minimising loss of granularity."
4. **"for possible decanting for alternative care settings"** → revise to avoid repetition of "for":
   *"...for possible decanting **to** alternative care settings."*

Let me know if you'd like a full stylistic pass as well.

4o

You said:

Descriptive analysis was conducted for patient characteristics. Charlson Comorbidity Index (CCI) was categorized into none, mild (1–2), moderate (3–4), and severe (≥5)34; systolic blood pressure into hypotension (<90 mmHg), normal (90–130 mmHg), and hypertension (>130 mmHg); pulse rate into bradycardia (<60 bpm), normal (60–90 bpm), and tachycardia (>90 bpm); and temperature into hypothermia (<35.5°C), normal (35.5–37.5°C), and hyperthermia (>37.5°C). Categorical and binary variables were summarised as numbers and percentages, while continuous variables were reported as means with standard deviations. Missing data was assessed across variables to determine the need for imputation. To determine the association of patient characteristics with cluster membership, multivariable ordinal logistic regression was performed, using cluster assignment as the outcome variable. Variables in the regression model included age, sex, ethnicity, systolic blood pressure, pulse rate, temperature, Charlson Comorbidity Index (CCI), laboratory investigations ordered, past emergency department visits, inpatient admissions, surgeries, intensive care unit admissions, high dependency admissions, intermediate care area admissions, and infectious disease admissions within the last 6 months. CCI was determined based on 17 predefined comorbidities from the last five years of hospital discharge records35,36. ICD-10 codes were categorised based on their first three characters (e.g., A01), and grouped into standardised blocks based on the World Health Organization's classification system to create clinically meaningful categories37. Each block represented a predefined range of ICD-10 codes. For example, J00-J06 and L50-54 represented ‘acute respiratory infections’ and ‘urticaria and erythema’, respectively. Consequently, individual codes within the same block could belong to different clusters. To further reduce dimensionality, an iterative process was employed to identify the most frequently occurring blocks. Blocks were ranked by their frequency across the dataset to calculate the cumulative patient coverage achieved at different thresholds. Heatmaps were generated to visualise the proportional distribution of these blocks across clusters. Cumulative coverage thresholds (e.g., 60%, 70%, 80%, 90%, 100%) were applied, illustrating how block-level distributions evolved as lower-frequency categories were excluded (Figure 1). This approach allowed for identification of dominant ICD-10 blocks within clusters, offering a concise yet comprehensive representation of the data.

ChatGPT said:

Here are the suggested changes for spelling and grammar:

1. **"Charlson Comorbidity Index (CCI) was categorized"** → use British spelling for consistency:
   *"...CCI was **categorised**..."*

2. **"Missing data was assessed"** → subject-verb agreement:
   *"Missing data **were** assessed..."*

3. **"To determine the association of patient characteristics with cluster membership"** → consider rephrasing for conciseness:
   "To assess the association between patient characteristics and cluster membership..."

4. **"Variables in the regression model included..."** → consider replacing **"in"** with **"used in"** or **"entered into"**:
   *"Variables **used in** the regression model included..."*

5. **"intermediate care area admissions"** → consider hyphenating for clarity:
   "intermediate-care area admissions"

6. **"ICD-10 codes were categorised based on their first three characters (e.g., A01)"** → add comma for clarity:
   "...first three characters (e.g., A01), and grouped..."
7. **"to create clinically meaningful categories37"** → add a comma before the citation:
   "...categories,**"*
8. **"Heatmaps were generated to visualise"** → correct British spelling used; no change needed if UK spelling is intended.
9. **"offering a concise yet comprehensive representation of the data"** → consider replacing "representation of the data" with "overview of the data" for smoother phrasing (optional):
   *"...offering a concise yet comprehensive **overview** of the data."*

Let me know when you're ready to continue with the next chunk.

4o

You said:

The data that support the findings of this study are available from Pre-hospital & Emergency Research Centre, Duke-NUS Medical School. Restrictions apply to the availability of these data, which were used under license for this study. Data are available from the authors upon reasonable request, with the permission of Duke-NUS Medical School.

ChatGPT said:

Here are the suggested changes for spelling and grammar:

1. **"Pre-hospital & Emergency Research Centre"** → consider spelling out **"&"** in formal writing:
   *"Pre-hospital **and** Emergency Research Centre"*
2. **"used under license"** → use British spelling for consistency:
   *"used under **licence**..."*
3. **"with the permission of Duke-NUS Medical School"** → slightly more formal phrasing:
   "...subject to permission from Duke-NUS Medical School." (optional stylistic improvement)

Revised suggestion (with British spelling and formal tone):

"The data that support the findings of this study are available from the Pre-hospital and Emergency Research Centre, Duke-NUS Medical School. Restrictions apply to the availability of these data, which were used under licence for this study. Data are available from the authors upon reasonable request, subject to permission from Duke-NUS Medical School."

Let me know if you'd like help with any further edits.
